# Design and Synthesis of Bendamustine-Carbonic Anhydrase Inhibitors with Antiproliferative Effects in Clear Cell Renal Cell Carcinoma

**DOI:** 10.1021/acs.jmedchem.6c01549

**Published:** 2026-06-24

**Authors:** Gioele Renzi, Alessandro Tubita, Lorenzo Antonuzzo, Serena Pillozzi, Marta Ferraroni, Andrea Angeli, Claudiu T. Supuran

**Affiliations:** † Neurofarba Department, Sezione di Scienze Farmaceutiche, 9300 University of Florence, Via Ugo Schiff 6, Sesto Fiorentino, Florence 50019, Italy; ‡ Department of Experimental and Clinical Biomedical Sciences “Mario Serio”, University of Florence, Viale Morgagni, 50, Florence 50134, Italy; § Department of Chemistry “Ugo Schiff”, University of Florence, Via della Lastruccia 3-13, Sesto Fiorentino 50019, Italy

**Keywords:** Bendamustine, clear cell renal cell carcinoma, carbonic anhydrase, metalloenzyme, kidney cancer

## Abstract

Human carbonic anhydrase IX (hCA IX) is markedly overexpressed in clear cell renal cell carcinoma and plays a key role in establishing an acidic tumor microenvironment associated with intrinsic chemoresistance. Extracellular acidification limits the uptake and efficacy of several cytotoxic agents, including bendamustine, a bifunctional alkylating agent whose activity depends on intracellular accumulation and DNA cross-link formation. Herein, we report the design and synthesis of novel bendamustine-carbonic anhydrase inhibitor (CAI) hybrids aimed at combining CA IX targeting with DNA-damaging activity. Structural modification of the bendamustine butyric acid side chain enabled the introduction of CAI warheads while preserving the alkylating moiety. The resulting compounds were evaluated against hCA I, II, IX, and XII, displaying preferential inhibition of the tumor-associated isoforms. Selected derivatives (**13a** and **14c**) showed antiproliferative activity in 786-O and CAKI-1 cells, inducing cell-cycle arrest and reducing long-term proliferative capacity more effectively than the reference CA IX inhibitor SLC-0111.

## Graphical abstract

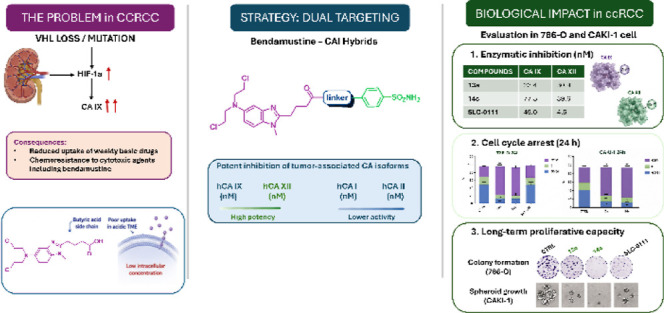

## Introduction

Globally, renal cancer represents the sixth in men and the ninth most common in women new cancer diagnosis and accounts for more than 430,000 new cases annually worldwide.^1,2^ Renal cell carcinoma (RCC) comprises a heterogeneous group of malignancies with clear cell renal cell carcinoma (ccRCC) representing the predominant histological subtype, accounting for approximately 75% of cases.^3^ Clinical stage at diagnosis is strongly correlated with prognosis. Patients with stage I disease have a 5-year survival rate of up to 93%, whereas those with stage IV disease have a markedly reduced survival rate of approximately 12%.^4^ Because early stage ccRCC is frequently asymptomatic, many patients present with advanced disease, contributing to persistently poor disease-specific survival. At the molecular level, ccRCC is characterized by frequent inactivation of the VHL (von Hippel-Lindau) tumor suppressor gene, occurring in approximately 80% of cases.^5,6^ Loss of VHL function results in stabilization of hypoxia-inducible factors (HIF-1α and HIF-2α), leading to constitutive activation of hypoxia-responsive pathways that promote tumor progression and immune evasion.^7–9^ Among HIF-regulated proteins, carbonic anhydrase IX (CA IX) plays a central role in the adaptation of tumor cells to hypoxic conditions. CA IX is a membrane-associated, zinc-dependent metalloenzyme that catalyzes the reversible hydration of carbon dioxide, thereby regulating intra- and extracellular pH.^10–12^ Its overexpression in more than 95% of ccRCC cases, coupled with minimal expression in normal tissue, makes CA IX an attractive target for selective therapeutic intervention.^13^ Functionally, CA IX contributes to extracellular acidification of the tumor microenvironment, which in turn limits the cellular uptake of weakly basic anticancer drugs through protonation-dependent mechanisms.^14,15^ This phenomenon is considered a key factor underlying the marked resistance of ccRCC to cytotoxic chemotherapy.^16,17^ This molecular background also helps explain why ccRCC is notably refractory to conventional cytotoxic chemotherapy and radiotherapy, for which clinical efficacy remains limited.^18,19^ However, clinical studies targeting CA IX with small molecules and monoclonal antibodies have shown limited effects on tumor growth when used as monotherapy.^20,21^ In contrast, several preclinical studies indicate that CA IX inhibition can partially restore tumor susceptibility to chemotherapeutic agents and combination strategies involving CAIX inhibition and cytotoxic drugs have demonstrated promising results.^22–24^

These findings support the rationale that simultaneous targeting of CA IX and additional vulnerabilities may represent an effective strategy to overcome the intrinsic chemoresistance of ccRCC. Bendamustine is a bifunctional alkylating agent combining a mechlorethamine moiety (bis­(2-chloroethyl)­amine) responsible for DNA cross-linking, a butyric acid side chain that enhances water solubility, and a benzimidazole ring that may confer antimetabolite-like properties.^25–27^ Unlike classical nitrogen mustards, the presence of the benzimidazole scaffold and the butyric acid side chain modulates the reactivity of the bis­(2-chloroethyl)­amine group, resulting in slower formation of reactive aziridinium intermediates and a more controlled alkylating profile. Consequently, bendamustine exhibits lower nonspecific macromolecular alkylation and a more favorable therapeutic index than highly reactive first-generation nitrogen mustards such as mechlorethamine.^25–27^ Moreover, bendamustine displays only partial cross-resistance with other alkylating agents, suggesting distinct interactions with cellular DNA damage response and repair pathways. These unique pharmacological properties have supported its successful clinical use in hematological malignancies and, to a lesser extent, in solid tumors.^27–30^ The combination of potent DNA-damaging activity and moderated electrophilic reactivity makes bendamustine an attractive scaffold for molecular hybridization strategies. Based on these considerations, we hypothesized that incorporation of carbonic anhydrase inhibitor (CAI) pharmacophores into the bendamustine scaffold could represent an effective approach to overcome pH-mediated drug resistance in clear cell renal cell carcinoma (ccRCC). By simultaneously targeting tumor-associated carbonic anhydrases and DNA integrity, such hybrids are expected to modulate the acidic tumor microenvironment, enhance intracellular drug accumulation, and preserve the cytotoxic activity of the alkylating agent.

## Results and Discussion

### Compounds Design and Synthesis

Bendamustine provides a suitable scaffold for derivatization toward multitarget compounds. The butyric acid side chain, which primarily contributes to aqueous solubility, represents an appropriate site for functional modification. Introduction of CAI warheads, such as sulfonamide or coumarin motifs known to selectively inhibit the tumor-associated isoforms CA IX and CA XII, can potentially confer high affinity toward these targets while preserving the mechlorethamine moiety responsible for DNA alkylation. Such bendamustine-CAI hybrids are hypothesized to operate through dual coordinated mechanisms. Integrating the inhibition of tumor-associated CAs with the induction of DNA damage within a single molecular framework aims to overcome microenvironment-driven chemoresistance and transform a classic alkylating agent into a tumor-targeted cytotoxic compound, particularly suited to hypoxic and CA IX-positive tumors such as ccRCC. Chemically, bendamustine hydrochloride (**1**) was functionalized exclusively at its carboxylic acid group, enabling amide bond formation through coupling reactions with intermediates bearing primary or secondary amino functionalities. With the exception of commercially available aminobenzenesulfonamides **2a**–**2c** and **5**, intermediates **3a**,**3b** and **4** were synthesized as previously described.^31,32^

CAI warheads containing primary amino groups were coupled to **1** using HATU as the coupling reagent and DIPEA in DMF, affording the corresponding amide derivatives (**6**–**9**) as outlined in Scheme 1.

**Scheme 1:**
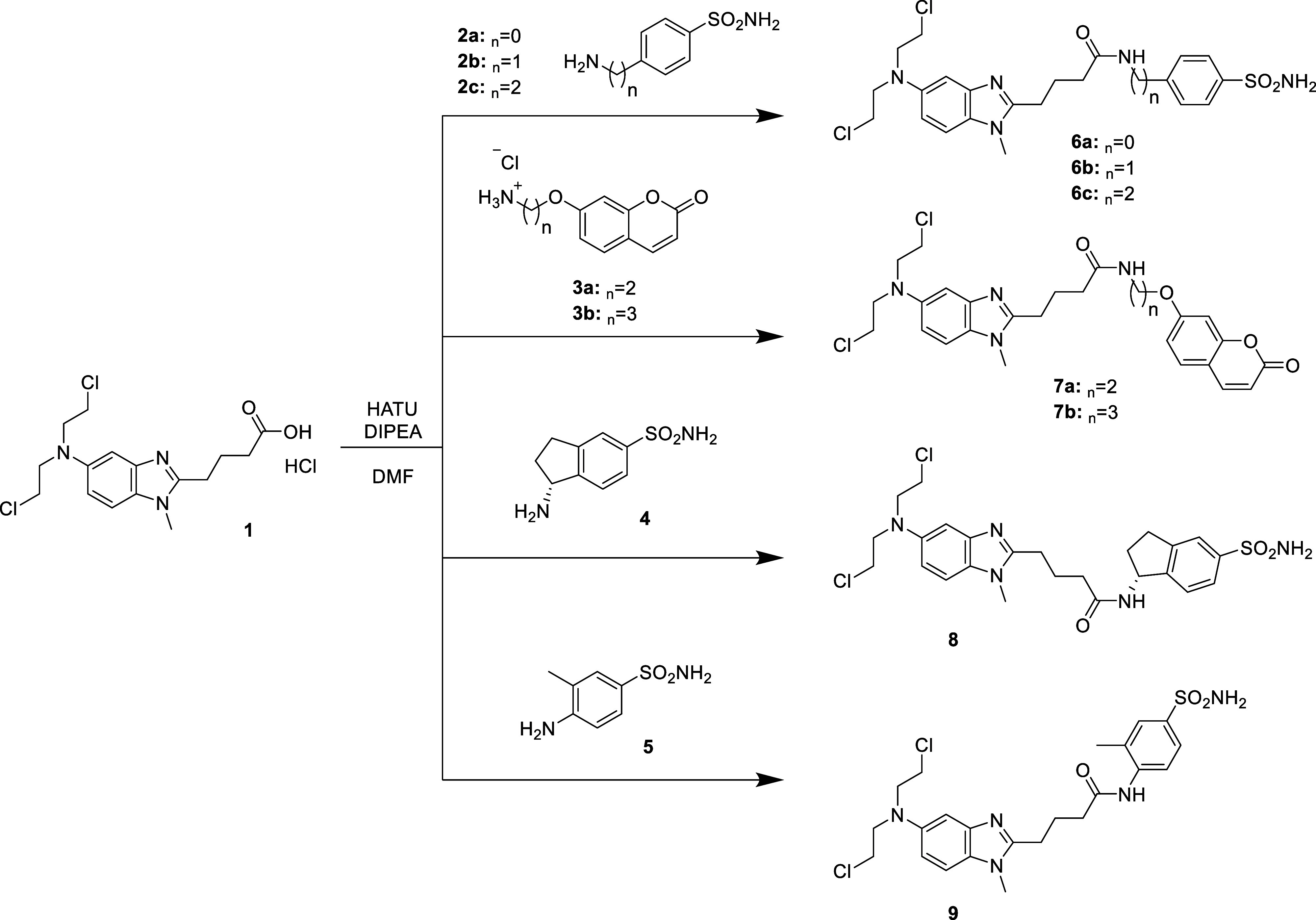
General Synthesis of Compounds **6**–**9**

Subsequently, to further explore structure–activity relationships, longer spacers between the two pharmacophores were introduced, incorporating piperazine or 1,4-diazepane rings. The corresponding secondary amine intermediates **10**–**12** were obtained through nucleophilic substitution reactions between appropriately substituted 4-fluorobenzenesulfonamides (**16a**–**h**) and piperazine (**17**) or 1,4-diazepane (**18**) in refluxing water, affording the desired products in high yields (Scheme 2), as previously reported by our group.^33^

**Scheme 2:**
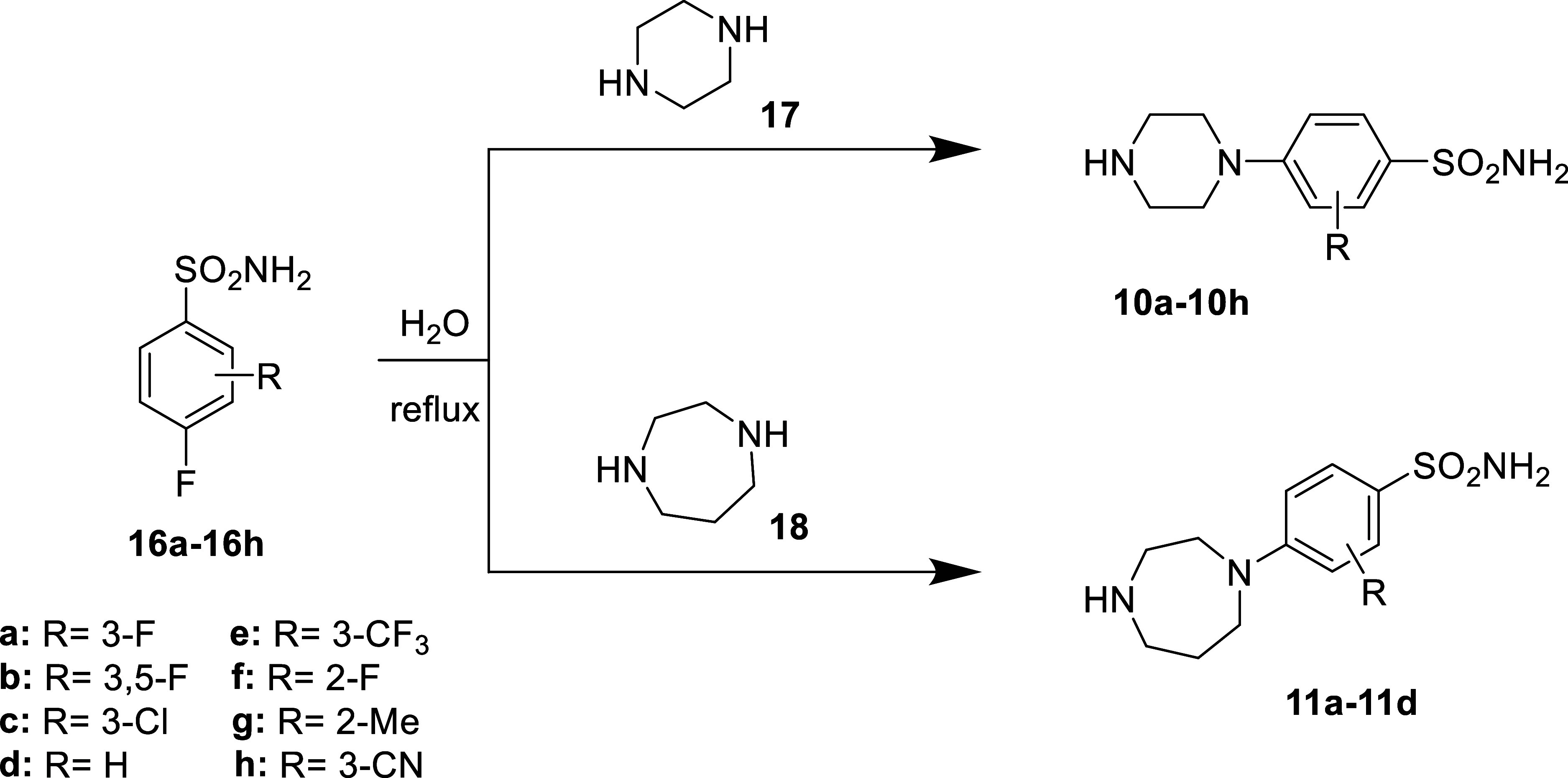
General Synthesis of Compounds **10a**–**h** and **11a**–**d**

Intermediate **12** was synthesized following the reported synthetic pathway outlined in Scheme 3. In the first step, commercially available 1-Boc-piperazine (**19**) was coupled with 3-(4-sulfamoylphenyl)­propanoic acid (**20**) using PyBOP as coupling agent and DIPEA in DMF, yielding compound **21** in quantitative yield. Subsequent deprotection with trifluoroacetic acid afforded the free secondary amine as its hydrochloride salt **12**.

**Scheme 3:**
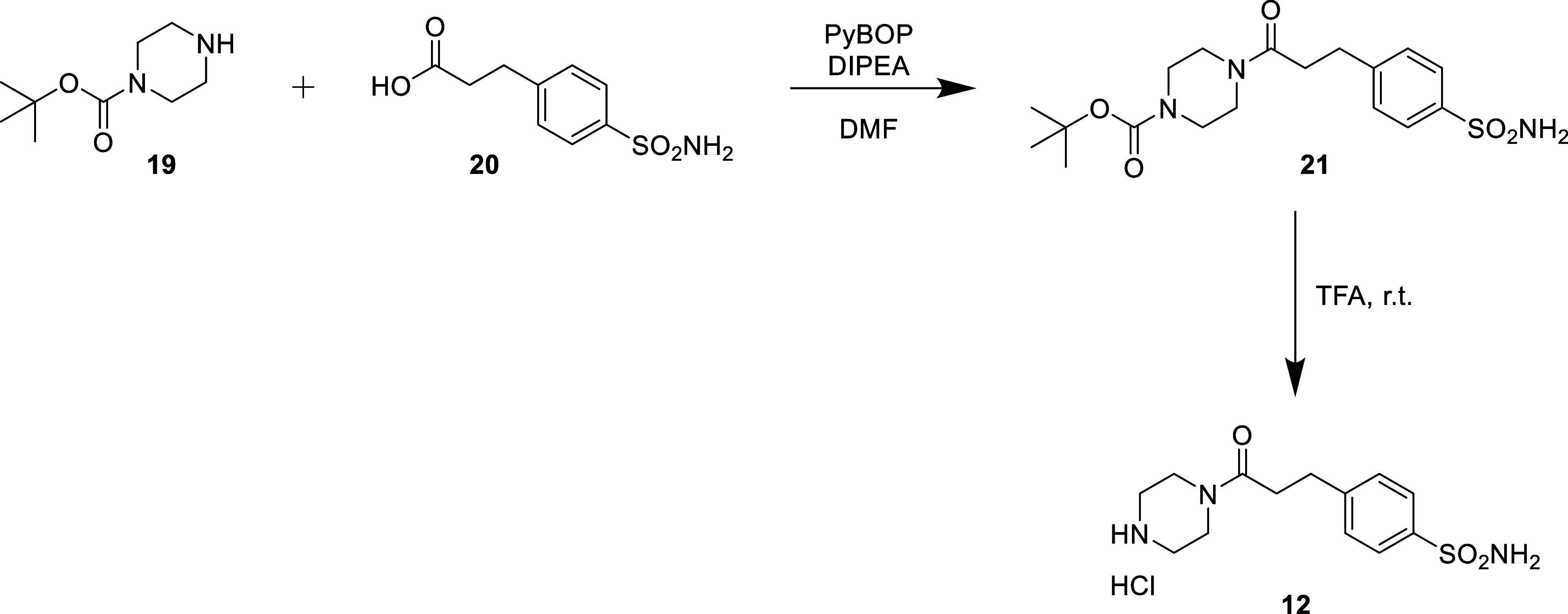
General Synthesis of Compound **12**

These intermediates were subsequently conjugated with bendamustine hydrochloride (**1**) following a procedure analogous to that previously described. Coupling reactions were performed using PyBOP as the activating agent and DIPEA in DMF, yielding the corresponding amide-functionalized derivatives **13**–**15** in good yields (Scheme 4).

**Scheme 4:**
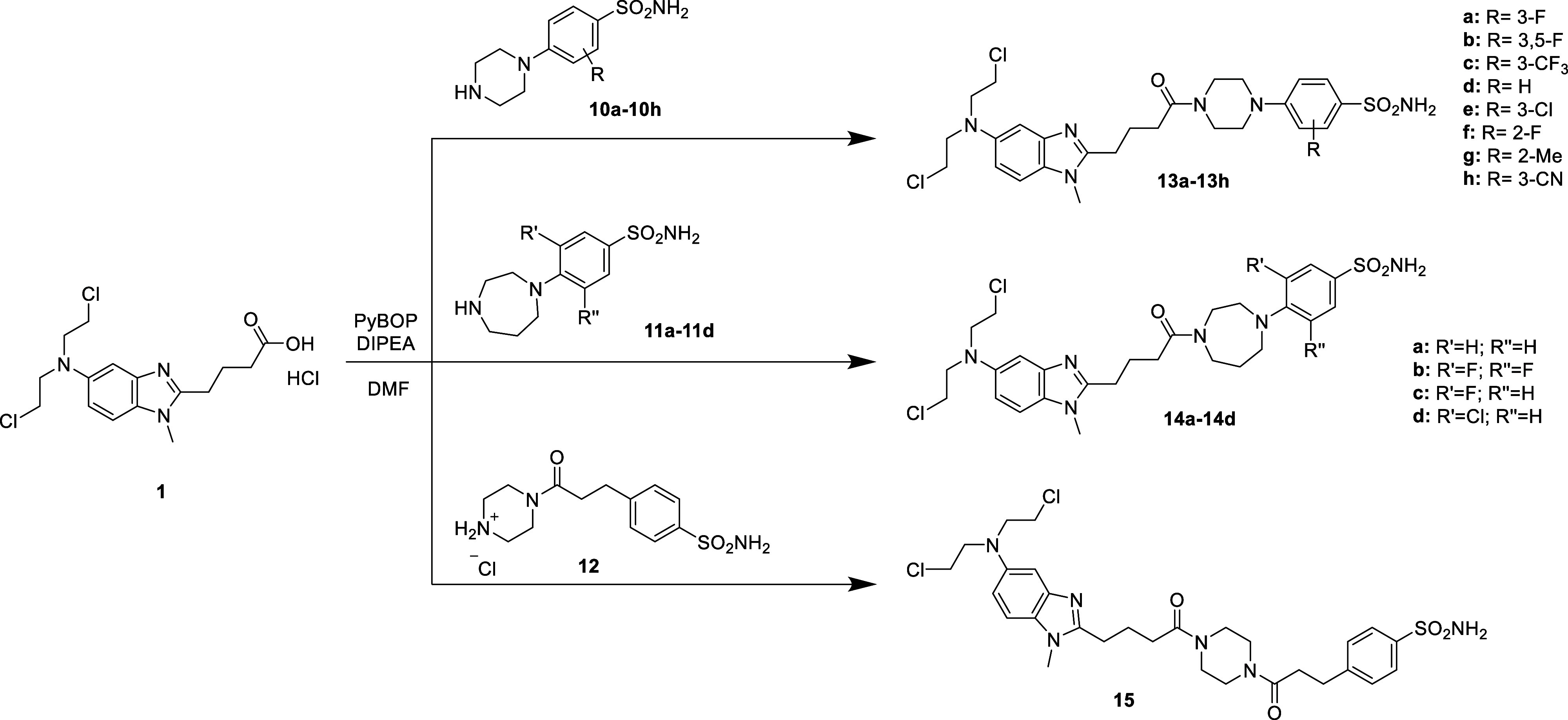
General Synthesis of Compounds **13**–**15**

### Carbonic Anhydrase Inhibition

All synthesized compounds were evaluated in vitro through the stopped-flow CO_2_ hydration assay to determine either inhibitory activity and selectivity profiles against the off-target CA isoforms hCA I, II, and the tumor-associate isoform CA IX and XII. Resulting inhibition constants (K_I_) are summarized in Table 1 and they are shown compared to the primary sulfonamide drug Acetazolamide (**AAZ**) and **SLC-0111** as reference compounds.

**Table 1:**
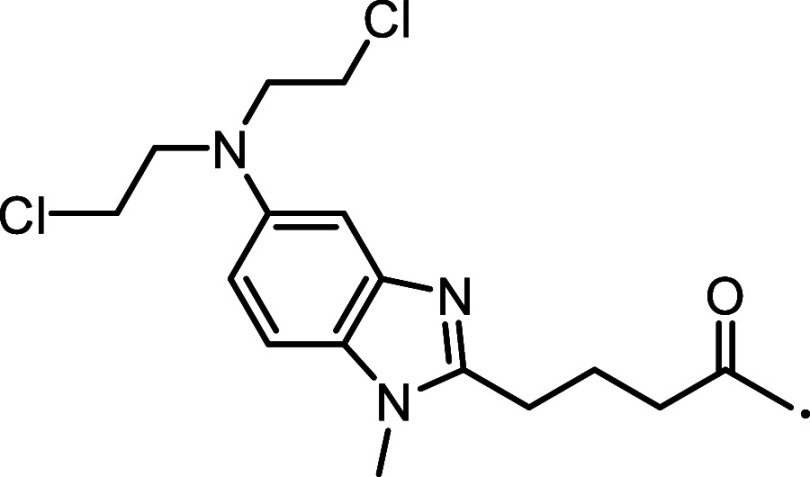

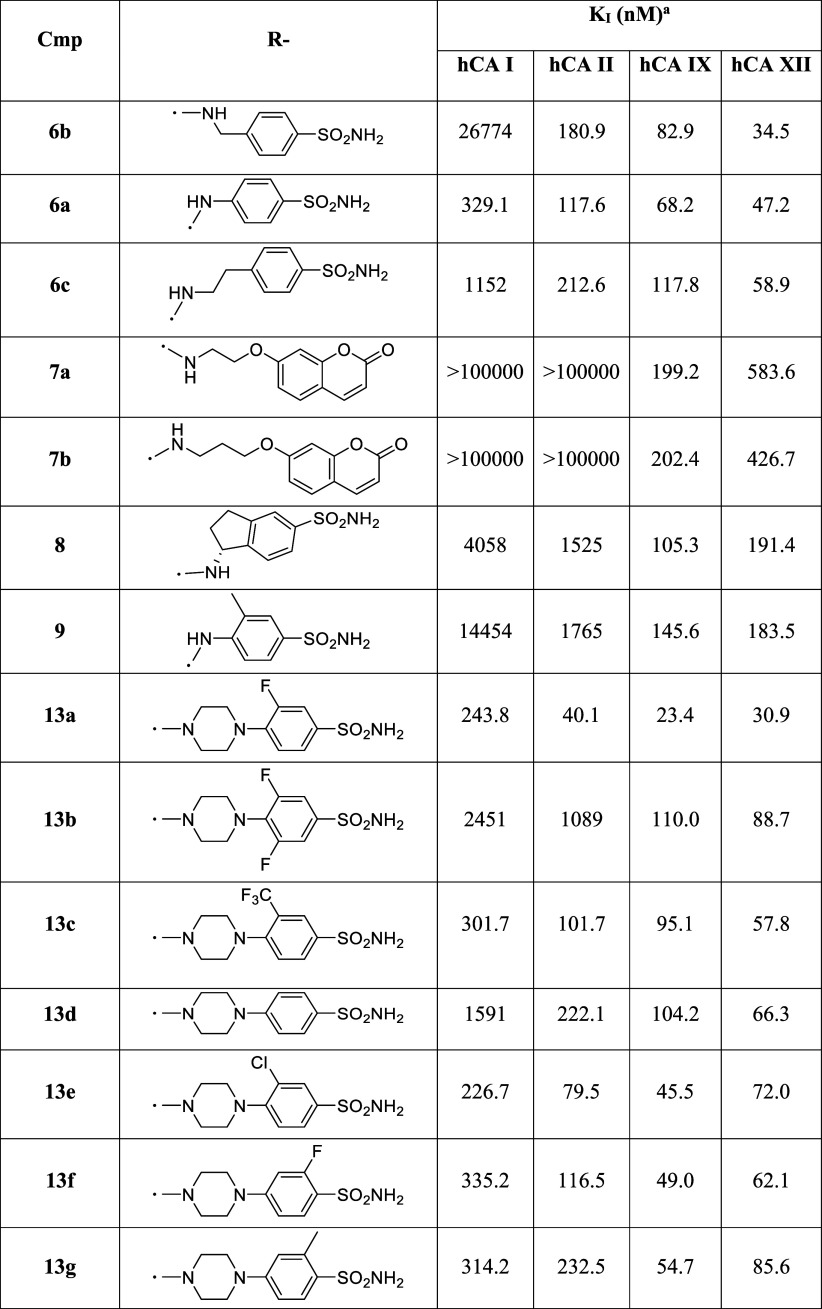

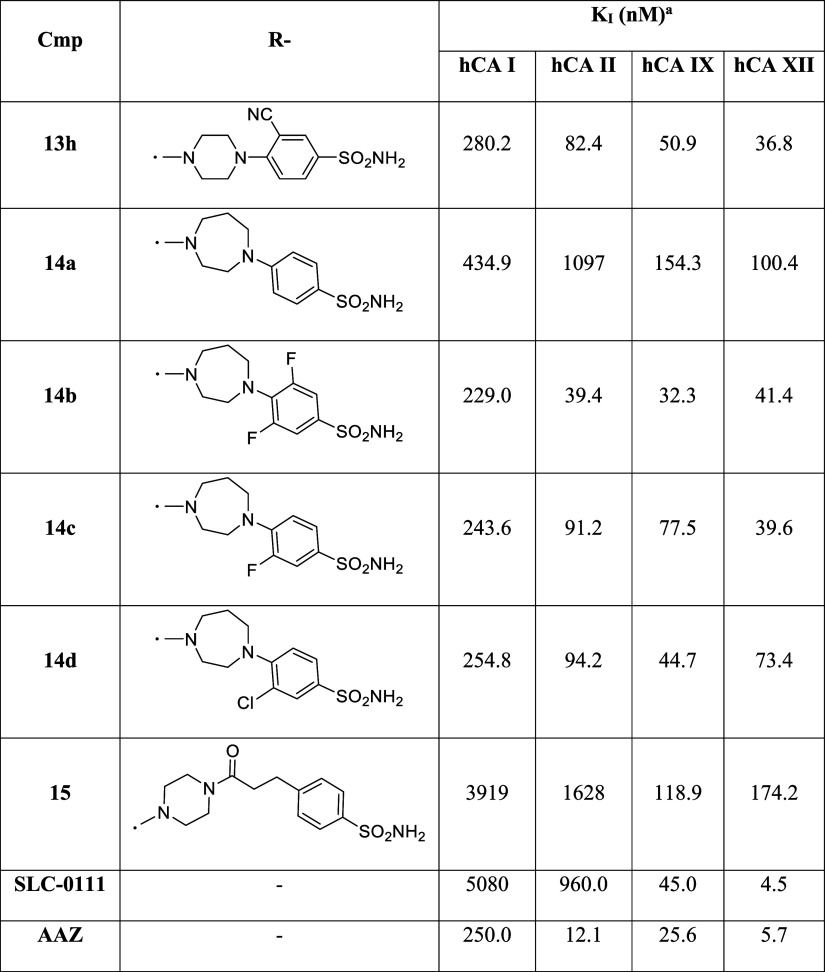
Inhibition of Human Isoforms hCA I, II, and Membrane-Associated IX and XII, by a CO_2_ Hydrase, Stopped-Flow Assay^34^ Using **AAZ** and **SLC-0111** as a Reference Drugs^a^

a Mean from 3 different assays, by a stopped-flow technique (errors were in the range of ±5–10% of the reported values).

The secondary amide derivatives **13**–**15** generally displayed weak inhibitory activity against the hCA I isoform, suggesting unfavorable interactions within the enzyme active site. Most compounds in this series exhibited inhibition constants in the micromolar range with K_I_ spanning from 1152 and 26774 nM. Within this subset **6a** showed moderate potency with a K_I_ value of 329.1 nM. In contrast, the introduction of an aliphatic spacer between the amide functionality and the aromatic ring was detrimental to activity. The homologous derivatives **6b** and **6c** displayed significantly reduced inhibitory potency, showing 81-fold and 3.5-fold losses in activity with K_I_ values of 26774 nM for **6b** and 1152 nM for **6c**. Interestingly, the introduction of a methyl substituent at the meta position of the aromatic ring of the CA-targeting warhead led to drastically reduction in potency reaching micromolar range (K_I_s of 329.1 and 14454 nM for **6a** and **9** respectively). In contrast, compounds bearing the coumarin moiety as CA-targeting warhead were essentially inactive toward hCA I (K_I_s > 100000 nM).^35^ Further elongation of the scaffold through the incorporation of a piperazine spacer directly linked to the benzenesulfonamide moiety resulted in a modest improvement in inhibitory potency. Within this series, most of the compounds showed high-nanomolar range activity (i.e., K_I_s between 226.7 and 335.2 nM). Nevertheless, three of them displayed lower affinity for hCA I, with inhibition constants in the low micromolar range (K_I_s of 2451, 1591, and 3919 nM for **13b**, **13d** and **15** respectively). Among these derivatives, **13e** emerged as the most effective inhibitor, showing a K_I_ value of 226.7 nM. Replacement of the chlorine atom with a fluorine one, as well as the introduction of a trifluoromethyl group, unaffected the inhibitory affinity. Conversely, introducing a second fluorine atom on the aromatic ring led to a 10-fold loss of activity compared to the corresponding monosubstituted compound (K_I_s of 2451 and 243.8 nM for **13b** and **13a** respectively), as well as the removal of any aromatic ring substituents (K_I_s of 1591 nM for **13d**). Interestingly, shifting of the fluorine substituent from meta to ortho position led to small effects (K_I_s of 243.8 and 335.2 nM for **13a** and **13f** respectively). Replacement of the piperazine linker with a bulkier 1,4-diazepane ring did not compromise the activity, despite a totally different orientation of the molecule due to steric hindrance. Compounds in this series exhibited good inhibition constants spanning from 229.0 to 434.9 nM. Notably **14b**, featuring a 3,5-difluoro-substituted benzenesulfonamide, emerged as the most potent, leading to a 1.9-fold increase in the activity compared to the nonsubstituted analogue (K_I_s of 434.9 and 229.0 nM for **14a** and **14b** respectively).

Regarding hCA II inhibition, the first series of compounds showed activity spanning from the high-nanomolar to low-micromolar range (i.e., K_I_s between 117.6 and 1765 nM). Consistent with the trend observed for hCA I, compound **6a** turned out to be the most potent with K_I_ value of 117.6 nM. Introduction of a methyl substituent of the aromatic ring on the pharmacophore resulted in a substantial 15-fold reduction in potency (K_I_s of 117.6 and 1765 nM for **6a** and **9** respectively). In contrast, elongation of the spacer between the amide group and the benzenesulfonamide moiety did not affect the activity, with K_I_ values ranging between 117.6 and 212.6 nM for compounds **6a** and **6c** respectively. As previously noted for hCA I, coumarin-based derivatives were ineffective with K_I_ values > 100000 nM.

Compounds incorporating a piperazine linker exhibited superimposable inhibition profile, albeit some interesting results were highlighted. Most derivatives displayed potent activity, with K_I_ values ranging between 40.1 and 232.5 nM although two derivatives showed low micromolar range inhibition constants (K_I_s of 1089 and 1765 nM for **13b** and **15** respectively). Within this set, **13a** was the most potent, achieving a 5.5-fold enhancement in potency compared to compound lacking substituents (K_I_s of 40.1 and 222.1 nM for **13a** and **13d** respectively). Moreover, electron-withdrawing substituents (EWG), such as chlorine (**13e**) or a cyano group (**13h**) maintained inhibitory potency (i.e., K_I_ of 79.5 and 82.4 nM respectively). In contrast, replacement of fluorine with a trifluoromethyl group caused only a modest decrease in activity (K_I_s of 40.1 and 101.7 nM for **13a** and **13c** respectively), whereas the addition of a second fluorine atom at the 5-position was detrimental for the activity, reducing activity by 27-fold (K_I_s of 40.1 and 1089 nM for **13a** and **13b** respectively). In the 1,4-diazepane-based series, compound **14a** showed poor affinity with inhibition constant value only reaching low micromolar range. However, introduction of aromatic substituents drastically enhanced inhibition by 2 orders of magnitude, with compound **14b** as the most effective one, showing 28-fold increased activity.

Evaluation of the tumor-associated isoform hCA IX showed a generally enhanced inhibitory features, lowering K_I_ values within mid- to high nanomolar range (i.e., K_I_s between 23.4 and 199.2 nM), highlighting a remarkable selectivity compared to previously reported CA isoforms. Within the secondary amide series, **6b** was the most potent inhibitor. Spacer elongation was tolerated only up to one methylene unit (K_I_s of 68.2 and 82.9 nM for **6a** and **6b** respectively), whereas further extension resulted in a 1.7-fold reduction in activity (K_I_s of 68.2 and 117.8 nM for **6a** and **6c** respectively). Noteworthy, **8** and **9**, which were weak inhibitors of hCA I and II, exhibited improved potency against hCA IX, reaching K_I_s in the high nanomolar range (K_I_s of 105.3 and 145.6 nM respectively). As expected, coumarin-based derivatives displayed good inhibition profile toward hCA IX, with both compounds showing high-nanomolar range activity (K_I_s of 199.2 and 202.4 nM for **7a** and **7b** respectively). In the second set of compounds, tertiary amide derivatives exhibited astonishing results, following the optimal trend observed for hCA II. Specifically, 3-halogen substitution on the benzenesulfonamide ring significantly enhanced potency, either with chlorine atom (i.e., K_I_ = 45.5 nM for **13e**) and fluorine atom (i.e., K_I_ = 23.4 nM for **13a**). In contrast, 3,5-difluoro substitution unaffected the activity (K_I_s of 110.0 and 104.2 nM for **13b** and **13d** respectively). Neither group switching nor shifting position of the aromatic substituent was detrimental for the activity, keeping inhibition constant values within 100 nM (K_I_s of 49.0, 54.7, and 50.9 nM for **13f**, **13g** and **13h** respectively). The 1,4-diazepane-containing compounds showed comparable inhibition profile, with compound **14a**, lacking aromatic ring substituents, being the least active. Introduction of aromatic substituents afforded notable effects, leading to respectively 4.8-, 2.0-, 3.5-fold gain of activity (K_I_s of 32.3, 77.5, and 44.7 nM for **14b**, **14c** and **14d** respectively).

Finally, inhibition data for hCA XII further confirmed the strong affinity of Bendamustine-CA hybrid molecules toward tumor-associated isoforms. No clear SAR trends emerged for compounds **6a**–**6c** as all derivatives displayed comparable potency (i.e., K_I_s between 34.5 and 58.9 nM). Compounds **8** and **9**, as well as coumarin-based analogues, showed inhibition results comparable with that observed for hCA IX, retaining good selectivity ratio despite higher K_I_ values between 183.5 and 583.6 nM. Concerning the set of piperazine-based derivatives, all tested compounds exhibited enhanced affinity toward hCA XII, with all compounds achieving midnanomolar range inhibition (i.e., K_I_s between 30.9 and 88.7 nM) confirming their affinity for tumor-associated isoforms. Finally, 1,4-diazepane-based derivatives showed comparable potency to that observed for hCA IX. Among them, **14c** emerged as the most potent compound, with K_I_ value of 39.6 nM. Concerning this isoform, neither addition of a fluorine atom nor chlorine substitution did affect the affinity, maintaining a remarkable affinity for this isoform, consistently ranging between 39.6 and 73.4 nM.

### Crystallographic Study

To elucidate the molecular basis of CA inhibition by sulfonamide-linked bendamustine derivatives, we determined the X-ray crystal structure of hCA II in complex with compound **14b**. Following initial refinement, a well-defined electron density map (Figure S1) within the active site clearly indicated that the benzenesulfonamide moiety directly coordinates the catalytic zinc ion via its deprotonated sulfonamide group, consistent with the canonical binding mode of this inhibitor class^36,37^ (Figure 1A).

**Figure 1:**
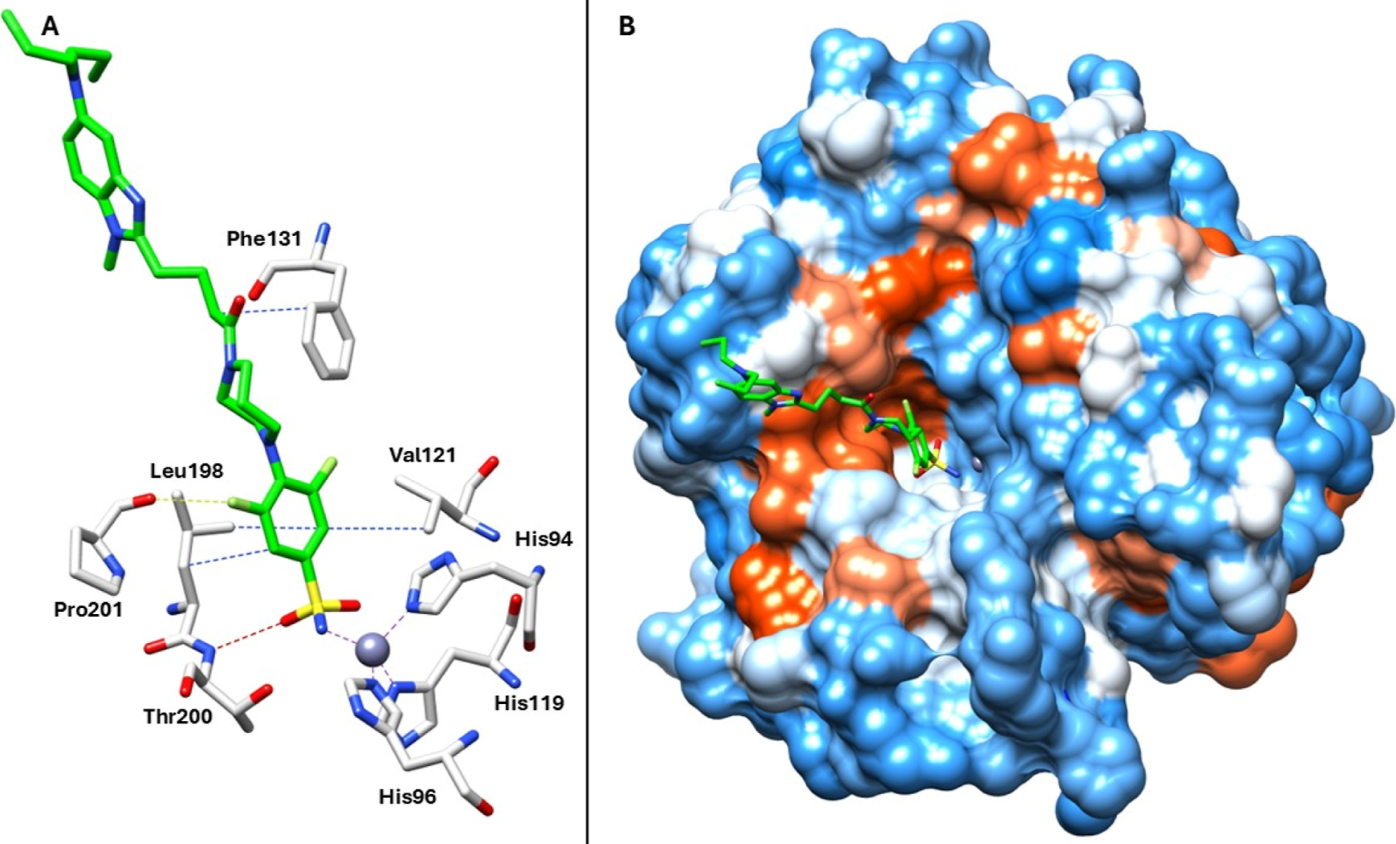
(A) X-ray crystal structures of hCA II bound with compound **14b** (PDB: 30NN). Residues involved in the binding of inhibitors are also shown; the gray sphere represents the zinc ion in the active site of the proteins. van der Waals interactions are shown in blue, hydrogen bond is shown in red and halogen bond interaction is shown in yellow. (B) **14b** inside the active site of hCA II. Hydrophobic (red) and hydrophilic (blue) residues are labeled.

In addition, a hydrogen bond was observed between the backbone amide of Thr199 and one of the sulfonamide oxygen atoms. This interaction, together with hydrophobic contacts involving Val121 and Leu198, contributes to stabilization of the benzenesulfonamide scaffold within the enzyme active site.^36,37^ Additionally, a halogen bond between one of the fluorine atoms of the inhibitor and Pro201 was also identified, a feature previously reported for aromatic fluorinated benzenesulfonamide derivatives.^38^ Notably, interactions involving the bendamustine moiety were limited; only a hydrophobic contact with Phe131 was observed (Figure 1A), which appears to orient this portion toward the hydrophobic region of the active site. Interestingly, the nitrogen mustard group adopts an orientation opposite to His64, suggesting it does not directly participate in enzyme inhibition (Figure 1B). These findings confirm that the sulfonamide moiety is the only pharmacophore responsible for CA inhibition, while the bendamustine tail likely plays a secondary role in molecular positioning rather than direct binding.

### Effects of CA IX Inhibitors on Cell Viability

Prior to evaluating the biological activity of the newly synthesized CA IX inhibitors, CA IX expression levels were assessed in RCC cell lines (Figure 2A). The selected models included 786-O cells, harboring VHL-mutations and therefore expected to exhibit elevated CA IX expression, and CAKI-1 cells, which are VHL wild-type (WT) and typically associated with lower CA IX levels. Unexpectedly, both cell lines expressed comparable levels of CA IX protein, indicating no detectable differences in CA IX abundance despite their distinct VHL status. To explore the potential translational relevance of these findings, the effects of the new CA IX inhibitors **13a** and **14c**, together with the reference CA IX inhibitor SLC-0111 were evaluated on cell viability in both 786-O and CAKI-1 cell lines. Dose–response analyses revealed a concentration-dependent reduction in cell viability for both **13a** and **14c** across the two cell models (Figure 2B,C).

**Figure 2:**
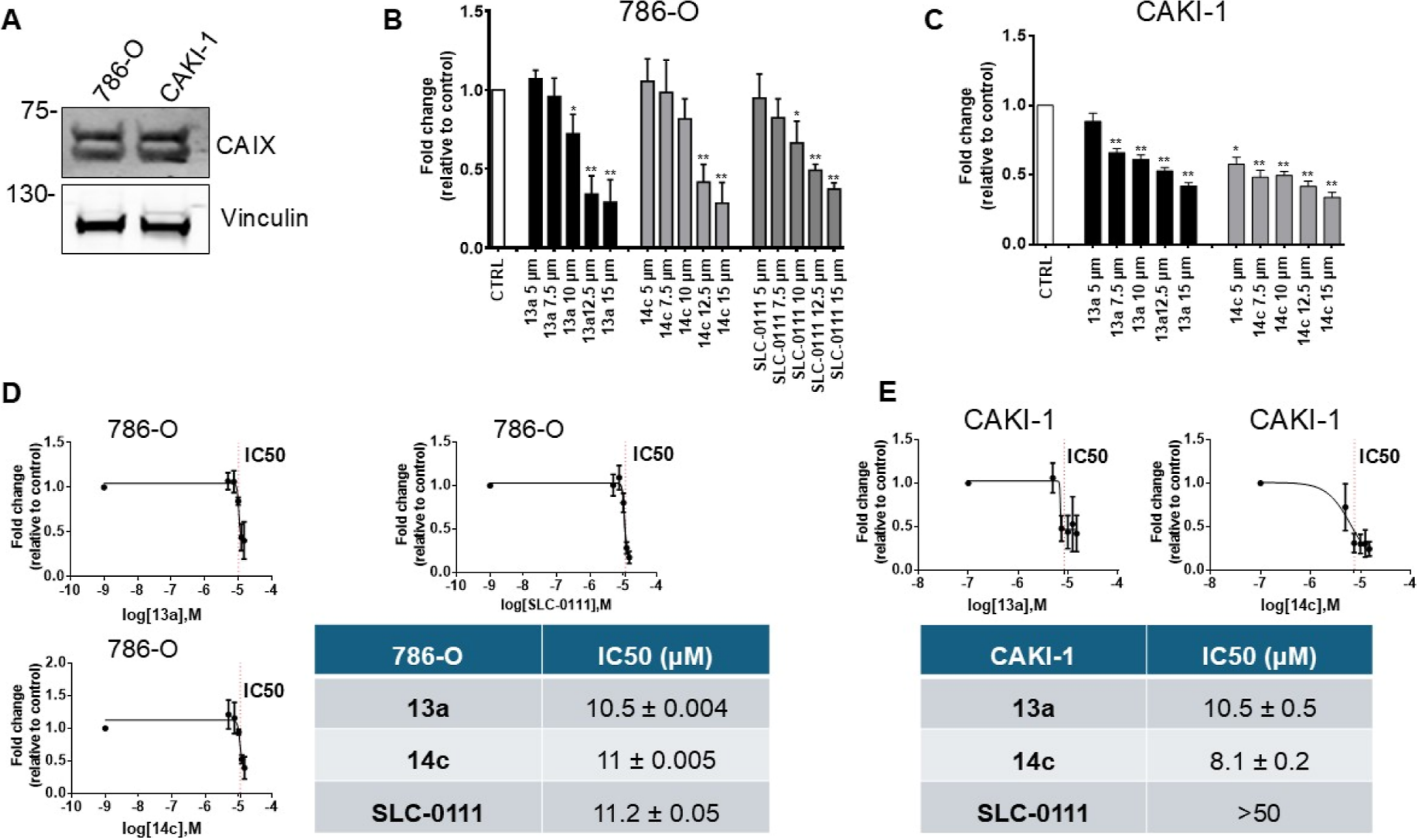
(A) CA IX expression levels in 786-O and CAKI-1 cell lines. Cell viability of 786-O (B) and CAKI-1 (C) RCC cell lines treated with **13a**, **14c**, and **SLC-0111** inhibitors for 72 h. IC_50_ values of **13a**, **14c** and **SLC-0111** in 786-O cells (D) and in CAKI-1 cells (E).

Chemosensitivity was quantified by IC_50_ values, defined as the concentration required to reduce cell viability by 50%. In 786-O cells, all three compounds exhibited comparable potency, with IC_50_ values of 10.5 ± 0.004 μM for **13a**, 11 ± 0.005 μM for **14c**, and 11.2 ± 0.05 μM for SLC-0111 (Figure 2D). In contrast, in CAKI-1 cells, **14c** demonstrated slightly improved potency (IC_50_ = 8.1 ± 0.2 μM) compared to **13a** (IC_50_ = 10.5 ± 0.5 μM), whereas SLC-0111 did not reach measurable inhibition within the tested concentration range (IC_50_ > 50 μM; Figure 2E). Consistent with these observations, treatment with **13a** and **14c** resulted in significant reductions in cell viability relative to control conditions, with statistically significant effects observed at multiple concentrations. Overall, these data indicate that the newly developed CA IX inhibitors retain antiproliferative activity comparable to or exceeding that of SLC-0111, particularly in CAKI-1 cells.

### Pharmacological Inhibition of CA IX Altered RCC Cell Cycle Distribution

Western blot analyses were performed to assess CA IX protein levels following 24h compound exposure. In both 786-O and CAKI-1 cells, treatment with **13a** and **14c** did not alter CA IX expression levels compared to control conditions (Figure 3A). Densitometric quantification (numbers below the bands) confirmed only minor variations in CA IX protein levels, indicating that the observed biological effects are not associated with modulation of CA IX expression. In parallel, the expression of p21 and cleaved caspase-3 was assessed. Indeed, p21 is a key downstream effector of the DNA damage response and acts as a cyclin-dependent kinase inhibitor, promoting cell-cycle arrest and preventing the proliferation of cells harboring damaged DNA. Therefore, p21 upregulation is indicative of activation of cellular checkpoint pathways following treatment. In contrast, caspase-3 is a central executioner caspase in the apoptotic cascade, and its activation represents a hallmark of irreversible commitment to programmed cell death. Treatment with **13a** and **14c** resulted in increased p21 levels in both cell lines, suggesting activation of cell cycle regulatory pathways. Consistently, elevated levels of cleaved caspase-3 were detected upon treatment, indicating induction of apoptotic signaling. As evidenced by densitometric analysis, cleaved caspase-3 expression was markedly increased in 786-O cells treated with compound **14c**, whereas a more modest increase was observed following treatment with compound **13a**. A similar, although less pronounced, increase was also detected in CAKI-1 cells treated with either **13a** or **14c**. In contrast, SLC-0111 did not induce any appreciable change in cleaved caspase-3 levels or p21 levels in either cell line. Vinculin expression remained unchanged across all experimental conditions, confirming equal protein loading (Figure 3A).

**Figure 3:**
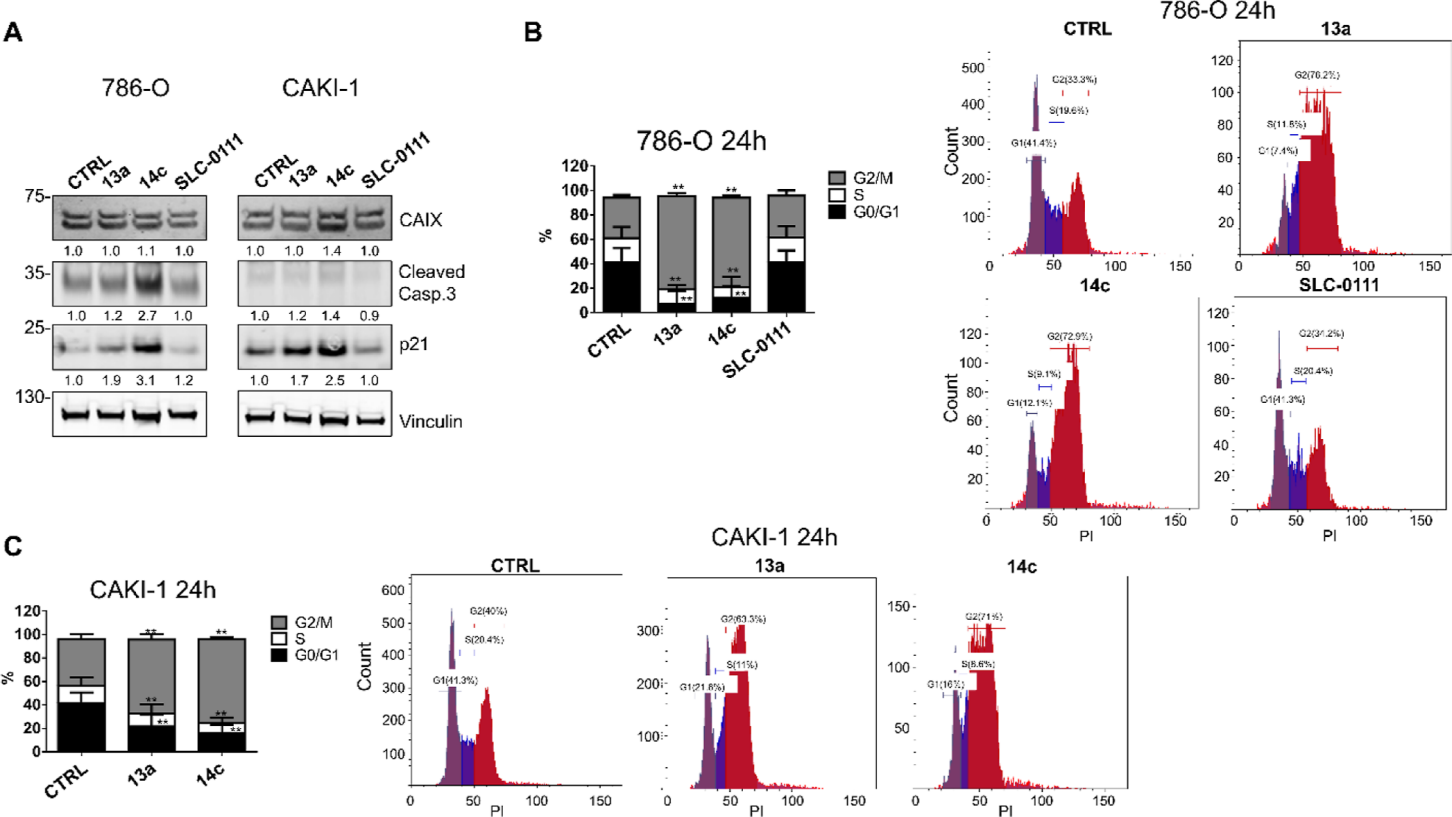
(A) CA IX, p21 and caspase-3 Western blot analyses after 24h compounds (**13a**, **14c** and **SLC-0111**) exposure at IC_50_ concentrations in both 786-O and CAKI-1 cells. The numbers below the bands indicate the relative densitometric values obtained with ImageJ. Target protein expression was normalized to Vinculin, and values are expressed relative to the control sample, which was set to 1.0. (**B**) Cell cycle distribution in 786-O cells at 24h after treatment with **13a**, **14c** and **SLC-0111**. (**C**) Cell cycle distribution in CAKI-1 cells after treatment with **13a**, **14c** and **SLC-0111**. **P < 0.01 refers to differences with respect to control (CTRL) as determined by Student *t* test.

Based on the observed modulation of p21, we next examined the cell cycle distribution of RCC cells following treatment with the different CA IX inhibitors. Cell cycle distribution was examined by propidium iodide (PI) staining. In 786-O cells, treatment with **13a** and **14c** for 24 h induced a marked accumulation of cells in the G2/M phase compared to control, accompanied by a reduction in the G0/G1 population (Figure 3B). In 786-O cells, SLC-0111 treatment for 24h did not produce appreciable changes in cell cycle distribution. In contrast, at 48h, a marked accumulation of cells in the G2/M phase was observed following treatment with **13a** and **14c**. Notably, cells treated with SLC-0111 also began to exhibit increased G2/M accumulation at this later time point, suggesting a delayed effect compared to the novel inhibitors (Figure S2). In CAKI-1 cells, compound **14c** produced a prominent increase in the G2/M fraction at 24 h, whereas **13a** elicited more moderate changes (Figure 3C). These findings indicate that the two novel CA IX inhibitors (**13a**, **14c**) exhibit greater efficacy respect to SLC-0111 in inducing cell cycle arrest in both the VHL mutant 786-O cell line and in the VHL-WT CAKI-1 cell line. Nevertheless, the elevated SLC-0111 IC_50_ in the CAKI-1 cell line prevented us from performing cell cycle analysis at the 24 h time point. The G2/M checkpoint is activated in response to DNA damage and serves to prevent entry into mitosis until genomic lesions have been repaired.^39^ The rapid induction of G2/M arrest observed after 24 h in 786-O cells, and particularly after treatment with **14c** in CAKI-1 cells, suggests that the bendamustine moiety retains its ability to induce DNA damage, triggering activation of cell-cycle checkpoint pathways. In contrast, SLC-0111 did not significantly alter cell-cycle progression at 24 h and only induced G2/M accumulation after prolonged exposure, indicating a delayed antiproliferative effect likely associated with disruption of pH homeostasis^40^ rather than direct genotoxic stress. The earlier and more pronounced G2/M arrest induced by **13a** and **14c** therefore supports the hypothesis that incorporation of the bendamustine scaffold provides an additional mechanism of action beyond CA IX inhibition alone. The persistence of G2/M arrest may ultimately lead to apoptotic cell death, as evidenced by the increased expression of cleaved caspase-3, and contribute to the reduced clonogenic potential observed in both ccRCC cell lines.

### 13a, 14c, and SLC-0111 Differentially Inhibit Clonogenic Growth and Spheroid Expansion in RCC Cell Lines

Clonogenic and 3D spheroid assays were performed to further characterize the antiproliferative effects of **13a**, **14c**, and SLC-0111 in RCC cell lines 786-O and CAKI-1. In colony formation assays, all compounds significantly reduced colony numbers compared with control conditions, indicating impaired long-term proliferative capacity. Notably, **13a** and **14c** exhibited a more pronounced inhibitory effect than SLC-0111 (Figure 4A).

**Figure 4:**
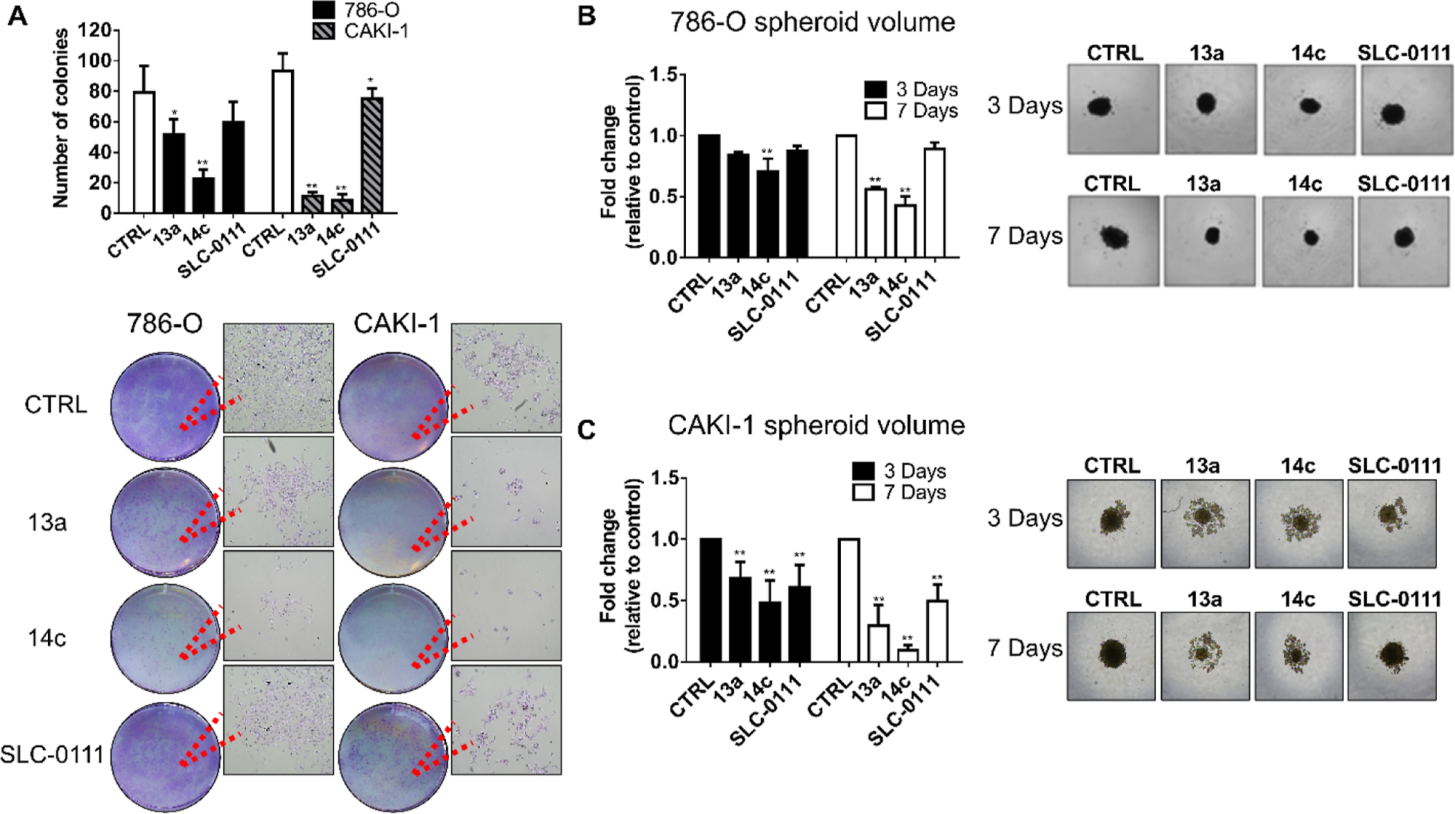
(**A**) Colony formation assays after treatment at IC_50_ concentrations with **13a**, **14c** and **SLC-0111** in both cell lines. Colonies were counted following crystal violet staining after 7 (786-O) or 10 (CAKI-1) days. Representative images of entire wells from 6-well plates are shown on the left. Enlarged views of representative individual colonies from the corresponding wells are shown on the right. The region displayed at higher magnification is indicated by the red dashed lines. 786-O (B) and CAKI-1­(C) spheroids were treated with **13a**, **14c** and **SLC-0111** at their IC_50_ for 3 or 7 days. Representative images of spheroids taken at day 3 and 7 days are shown. **P < 0.01 refers to differences with respect to control (CTRL) as determined by Student *t* test.

Consistently with previous results, spheroid growth analysis at 3 and 7 days demonstrated a significant reduction in spheroid volume in both models following treatments (Figure 4B,C). This effect was sustained or further amplified over time, supporting a time-dependent response. Overall, these data indicate that **13a** and, especially, **14c** effectively suppress clonogenic survival and 3D tumor growth in RCC models.

## Conclusions

In this work, a series of CAIs incorporating the bendamustine scaffold were successfully designed and synthesized to address microenvironment-driven chemoresistance in ccRCC. Hybridization of the two pharmacophores was achieved through amide bond formation at the butyric acid side chain of bendamustine, preserving the mechlorethamine moiety responsible for DNA alkylation while enabling the introduction of CAI warheads, including sulfonamide and coumarin motifs. The inhibitory activity of the synthesized compounds was evaluated against four hCA isoforms: the off-target cytosolic isoforms hCA I and hCA II, and the tumor-associated isoforms hCA IX and hCA XII. All compounds displayed good inhibition of hCA IX and XII in the low-to-medium nanomolar range, with K_I_ values for hCA IX spanning from 23.4 nM to 154.3 nM. Crystallographic analysis of compound **14b** in complex with hCA II revealed a distinct orientation of the tail within the enzyme active site and demonstrated that inhibition was exclusively mediated by the sulfonamide group. Importantly, the mechlorethamine moiety of bendamustine did not alkylate any amino acid residue, including the catalytically His64, confirming that the alkylating function remains chemically intact and does not interfere with CA binding. Functional studies in ccRCC cell lines showed that selected derivatives (**13a** and **14c**) reduce cell viability, induce G2/M cell cycle arrest, and activate apoptotic pathways. Furthermore, these compounds significantly impair clonogenic growth and spheroid expansion, outperforming the reference inhibitor SLC-0111 ^41^. Notably, differential responses were observed between CAKI-1 and 786-O spheroids, likely reflecting intrinsic differences in their molecular background and sensitivity to hypoxia-associated pathways. Given that 3D spheroid cultures develop hypoxic regions that promote CA IX expression, these findings further support the relevance of CA IX targeting in tumor microenvironments and suggest that the efficacy of these hybrids may be influenced by the extent of hypoxia-driven CA IX induction. Overall, this study demonstrates that bendamustine can serve as a versatile platform for the development of CA IX-targeted alkylating agents. The combination of CA inhibition and DNA-damaging activity within a single molecular framework provides a rational medicinal chemistry strategy to overcome pH-dependent drug resistance in CA IX-expressing tumors.

## Experimental Section

### General

Anhydrous solvents and all reagents were purchased from Sigma-Aldrich, VWR and TCI. All reactions involving air- or moisture-sensitive compounds were performed under a nitrogen atmosphere. Nuclear magnetic resonance (^1^H NMR, ^13^C NMR, ^19^F NMR) spectra were recorded using a Bruker Advance III 400 MHz spectrometer in DMSO-*d*_6_ or CDCl_3_. Chemical shifts are reported in parts per million (ppm) and the coupling constants (*J*) are expressed in Hertz (Hz). Splitting patterns are designated as follows: s, singlet; d, doublet; t, triplet; m, multiplet; brs, broad singlet; dd, double of doubles. The assignment of exchangeable protons (NH) was confirmed by the addition of D_2_O. Analytical thin-layer chromatography (TLC) was carried out on Merck silica gel F-254 plates. Flash chromatography purifications were performed on Merck silica gel 60 (230–400 mesh ASTM) as the stationary phase, and ethyl acetate, *n*-hexane, acetonitrile and methanol were used as eluents. The solvents used in MS measurements were acetone, acetonitrile (Chromasolv grade), purchased from Sigma-Aldrich (Milan, Italy), and mQ water 18 MΩ, obtained from Millipore’s Simplicity system (Milan, Italy). The mass spectra were obtained using a Varian 1200 L triple quadrupole system (Palo Alto, CA, USA) equipped with electrospray source (ESI) operating in both positive and negative ions. Stock solutions of analytes were prepared in acetone at 1.0 mg mL^–1^ and stored at 4 °C. Working solutions of each analyte were freshly prepared by diluting stock solutions in a mixture of mQ H_2_O/ACN 1/1 (*v/v*) up to a concentration of 1.0 μg mL^–1^ The mass spectra of each analyte were acquired by introducing, via syringe pump at 10 L min^–1^, the working solution. Raw data were collected and processed by Varian Workstation, version 6.8, software. Elemental analyses were performed with a Thermoscientific FlashSmart Elemental Analyzer CHN. All compounds reported here are >95% of purity.

#### General Procedure for the Synthesis of Compounds 6–9

Under an inert atmosphere (N_2_), Bendamustine hydrochloride **1** (1 equiv) was added to a solution of appropriate primary amine (1 equiv), HATU (1.1 equiv), and DIPEA (2 equiv) in DMF dry (2 mL). The reaction mixture was stirred at room temperature overnight. A control via TLC was performed to ensure the complete consumption of the starting materials. The reaction was quenched with saturated solution of NH_4_Cl, extracted with EtOAc three times. The combined organic layer was dried over Na_2_SO_4_ and concentrated under reduced pressure. The crude product was purified by flash chromatography (MeOH/DCM 5:95) to afford compounds **6**–**9**.

##### 4-(5-(bis­(2-Chloroethyl)­amino)-1-methyl-1*H*-benzo­[*d*]­imidazole-2-yl)-*N*-(4-sulfamoylphenyl)­butanamide (6a)

Following the General Procedure, Bendamustine hydrochloride **1** (51 mg, 0.12 mmol) and **2a** (22 mg, 0.12 mmol) gave **6a** as a light yellow solid. Yield 50%. ^1^H NMR (400 MHz, DMSO-*d*_6_): δ (ppm): 8.71 (1H, m, NH, exchange with D_2_O), 7.67 (2H, d, *J* = 8.90 Hz), 7.36 (1H, d, *J* = 8.77 Hz), 7.10 (2H, s, SO_2_NH_2_, exchange with D_2_O), 7.07 (2H, d, *J* = 9.00 Hz), 6.95 (1H, s), 6.82 (1H, dd, *J* = 8.74 Hz; *J* = 2.17 Hz), 3.74 (8H, m), 3.71 (3H, s), 3.30 (2H, m), 2.89 (2H, t, *J* = 7.42 Hz), 2.04 (2H, m); ^13^C NMR (100 MHz, DMSO-*d*_6_): δ (ppm): 171.4, 155.7, 153.4, 144.4, 143.1, 134.0, 130.3, 128.0, 114.8, 111.0, 110.8, 103.3, 55.4, 42.4, 32.5, 30.3, 26.9, 23.4; MS (ESI positive) *m*/*z*: 512.12 [M + H]^+^. Elemental Analysis: C, 51.37; H, 5.28; N, 13.76.

##### 4-(5-(bis­(2-Chloroethyl)­amino)-1-methyl-1*H*-benzo­[*d*]­imidazole-2-yl)-*N*-(4-sulfamoylbenzyl)­butanamide (6b)

Following the General Procedure, Bendamustine hydrochloride **1** (51 mg, 0.12 mmol) and **2b** (28 mg, 0.12 mmol) gave **6b** as a white solid. Yield 78%. ^1^H NMR (400 MHz, DMSO-*d*_6_): δ (ppm): 8.49 (1H, t, *J* = 5.89 Hz, NH, exchange with D_2_O), 7.81 (2H, d, *J* = 8.25 Hz), 7.46 (2H, d, *J* = 8.24 Hz), 7.37 (1H, d, *J* = 8.87 Hz), 7.34 (2H, s, SO_2_NH_2_, exchange with D_2_O), 6.96 (1H, d, *J* = 2.04 Hz), 6.83 (1H, dd, *J* = 8.78 Hz; *J* = 2.21 Hz), 4.37 (2H, d, *J* = 5.87 Hz), 3.75 (8H, m), 3.69 (3H, s), 2.85 (2H, t, *J* = 7.37 Hz), 2.34 (2H, t, *J* = 7.29 Hz), 2.05 (2H, m); ^13^C NMR (100 MHz, DMSO-*d*_6_): δ (ppm): 172.8, 155.6, 144.8, 144.3, 143.5, 143.2, 130.2, 128.4, 126.6, 111.1, 110.8, 103.2, 54.4, 42.6, 42.4, 35.4, 30.3, 26.9, 23.9; MS (ESI positive) *m*/*z*: 526.14 [M + H]^+^. Elemental Analysis: C, 52.52; H, 5.55; N, 13.38.

##### 4-(5-(bis­(2-Chloroethyl)­amino)-1-methyl-1*H*-benzo­[*d*]­imidazole-2-yl)-*N*-(4-sulfamoylphenethyl)­butanamide (6c)

Following the General Procedure, Bendamustine hydrochloride **1** (47 mg, 0.11 mmol) and **2c** (23 mg, 0.11 mmol) gave **6c** as a light brown solid. Yield 88%. ^1^H NMR (400 MHz, DMSO-*d*_6_): δ (ppm): 7.99 (1H, t, NH, exchange with D_2_O, *J* = 4.75 Hz), 7.77 (2H, d, *J* = 7.47 Hz), 7.42 (2H, d, *J* = 7.52 Hz), 7.37 (1H, s), 7.35 (2H, s, SO_2_N*H*_2_, exchange with D_2_O), 6.95 (1H, s), 6.82 (1H, d, *J* = 8.75 Hz), 3.74 (8H, m), 3.68 (3H, s), 3.33 (2H, m), 2.80 (4H, dd, *J* = 12.23 Hz; *J* = 6.01 Hz), 2.21 (2H, t, *J* = 7.07 Hz), 1.98 (2H, m); ^13^C NMR (100 MHz, DMSO-*d*_6_): δ (ppm): 172.7, 155.6, 144.7, 144.2, 143.3, 143.0, 130.2, 130.1, 126.6, 111.2, 110.9, 103.2, 54.5, 46.8, 42.4, 35.8, 35.5, 30.4, 26.9, 23.9; MS (ESI positive) *m*/*z*: 540.15 [M + H]^+^. Elemental Analysis: C, 53.41; H, 5.80; N, 13.00.

##### 4-(5-(bis­(2-chloroethyl)­amino)-1-methyl-1*H*-benzo­[*d*]­imidazole-2-yl)-*N*-(2-((2-oxo-2*H*-chromen-7-yl)­oxy)­ethyl)­butanamide (7a)

Following the General Procedure, Bendamustine hydrochloride **1** (53 mg, 0.13 mmol) and **3a** (40 mg, 0.13 mmol) gave **7a** as a white solid. Yield 41%. ^1^H NMR (400 MHz, DMSO-*d*_6_): δ (ppm): 8.18 (1H, t, *J* = 5.18 Hz), 8.01 (1H, d, *J* = 9.46 Hz), 7.65 (1H, d, *J* = 8.62 Hz), 7.36 (1H, d, *J* = 8.54 Hz), 7.03 (1H, s), 6.98 (1H, d, *J* = 8.66 Hz), 6.94 (1H, s), 6.83 (1H, d, *J* = 8.78 Hz), 6.31 (1H, dd, *J* = 9.48 Hz; *J* = 1.04 Hz), 4.13 (2H, t, *J* = 5.17 Hz), 3.74 (8H, s), 3.68 (3H, s), 3.49 (2H, m), 2.84 (2H, t, *J* = 7.25 Hz), 2.28 (2H, t, *J* = 7.27 Hz), 2.02 (2H, m); ^13^C NMR (100 MHz, DMSO-*d*_6_): δ (ppm): 173.0, 172.4, 162.5, 161.2, 156.3, 155.5, 145.2, 143.3, 130.4, 130.1, 113.6, 113.5, 113.4, 111.2, 110.9, 102.9, 102.2, 68.0, 54.4, 46.8, 42.3, 38.8, 35.4, 30.4, 26.8; MS (ESI positive) *m*/*z*: 545.16 [M + H]^+^. Elemental Analysis: C, 59.57; H, 5.58; N, 10.32.

##### 4-(5-(bis­(2-chloroethyl)­amino)-1-methyl-1*H*-benzo­[*d*]­imidazole-2-yl)-*N*-(3-((2-oxo-2*H*-chromen-7-yl)­oxy)­propyl)­butanamide (7b)

Following the General Procedure, Bendamustine hydrochloride **1** (52 mg, 0.13 mmol) and **3b** (42 mg, 0.13 mmol) gave **7b** as a white solid. Yield 43%. ^1^H NMR (400 MHz, DMSO-*d*_6_): δ (ppm): 8.01 (1H, d, *J* = 9.50 Hz), 7.96 (1H, t, *J* = 5.45 Hz), 7.65 (1H, d, *J* = 8.51 Hz), 7.36 (1H, d, *J* = 8.77 Hz), 7.00 (1H, d, *J* = 2.16 Hz), 6.97 (1H, dd, *J* = 8.53 Hz; *J* = 2.35 Hz), 6.94 (1H, d, *J* = 2.06 Hz), 6.82 (1H, dd, *J* = 8.78 Hz; *J* = 2.21 Hz), 6.31 (1H, d, *J* = 9.48 Hz), 4.13 (2H, t, *J* = 6.20 Hz), 3.74 (8H, m), 3.69 (3H, s), 3.25 (2H, dd, *J* = 12.47 Hz; *J* = 6.51 Hz), 2.83 (2H, t, *J* = 7.36 Hz), 2.25 (2H, t, *J* = 7.25 Hz), 2.01 (2H, m), 1.91 (2H, m); ^13^C NMR (100 MHz, DMSO-*d*_6_): δ (ppm): 172.6, 162.7, 161.2, 156.3, 155.5, 145.2, 144.0, 143.2, 130.4, 130.1, 113.6, 113.3, 113.2, 111.1, 110.9, 103.1, 102.0, 67.0, 54.4, 46.8, 42.3, 36.3, 35.5, 30.3, 29.6, 26.9; MS (ESI positive) *m*/*z*: 559.18 [M + H]^+^. Elemental Analysis: C, 60.11; H, 5.77; N, 10.01.

##### (*R*)-4-(5-(bis­(2-Chloroethyl)­amino)-1-methyl-1*H*-benzo­[*d*]­imidazole-2-yl)-*N*-(5-sulfamoyl-2,3-dihydro-1*H*-inden-1-yl)­butanamide (8)

Following the General Procedure, Bendamustine hydrochloride **1** (43 mg, 0.10 mmol) and **4** (22 mg, 0.10 mmol) gave **8** as a white solid. Yield 46%. ^1^H NMR (400 MHz, DMSO-*d*_6_): δ (ppm): 8.37 (1H, d, *J* = 8.20 Hz), 7.69 (1H, s), 7.44 (1H, d, *J* = 7.80 Hz), 7.37 (1H, d, *J* = 8.81 Hz), 7.35 (2H, s, SO_2_N*H*_2_, exchange with D_2_O), 6.96 (1H, d, *J* = 2.12 Hz), 6.83 (1H, dd, *J* = 8.73 Hz; *J* = 2.21 Hz), 5.40 (1H, q, N*H*, exchange with D_2_O, *J* = 7.74 Hz), 3.74 (8H, s), 3.71 (3H, s), 3.43 (2H, m), 3.01 (1H, m), 2.88 (2H, m), 2.45 (1H, m), 2.34 (2H, t, *J* = 7.16 Hz), 2.08 (2H, m), 1.88 (1H, m); ^13^C NMR (100 MHz, DMSO-*d*_6_): δ (ppm): 172.6, 155.6, 148.0, 146.2, 144.3, 143.6, 143.2, 130.3, 125.9, 125.8, 122.2, 111.1, 110.9, 103.2, 54.4, 54.1, 42.4, 35.5, 33.7, 30.6, 30.4, 26.9, 23.9; MS (ESI positive) *m*/*z*: 552.15 [M + H]^+^. Elemental Analysis: C, 54.38; H, 5.67; N, 12.70.

##### 4-(5-(bis­(2-chloroethyl)­amino)-1-methyl-1*H*-benzo­[*d*]­imidazole-2-yl)-*N*-(2-methyl-4-sulfamoylphenyl)­butanamide (9)

Following the General Procedure, Bendamustine hydrochloride **1** (60 mg, 0.14 mmol) and **5** (27 mg, 0.14 mmol) gave **9** as a light brown solid. Yield 23%. ^1^H NMR (400 MHz, DMSO-*d*_6_): δ (ppm): 7.48 (2H, m), 7.37 (1H, d, *J* = 8.80 Hz), 6.96 (1H, d, *J* = 2.11 Hz), 6.84 (1H, d, *J* = 8.75 Hz), 6.68 (1H, d, *J* = 9.13 Hz), 5.92 (2H, s), 3.75 (8H, s), 3.64 (3H, s), 2.76 (2H, t, *J* = 6.49 Hz), 2.34 (2H, t, *J* = 7.21 Hz), 2.11 (3H, s), 1.92 (2H, m); ^13^C NMR (100 MHz, DMSO-*d*_6_): δ (ppm): 171.8, 155.0, 152.7, 150.9, 143.4, 130.6, 129.8, 128.3, 125.0, 120.8, 113.1, 111.3, 111.0, 102.6, 54.3, 42.3, 35.4, 30.3, 26.2, 22.5, 18.3; MS (ESI positive) *m*/*z*: 526.14 [M + H]^+^. Elemental Analysis: C, 52.50; H, 5.57; N, 13.33.

#### General Procedure for the Synthesis of Compounds 13–15

Under an inert atmosphere (N_2_), Bendamustine hydrochloride **1** (1 equiv) was added to a solution of appropriate secondary amine (1 equiv), PyBOP (1.1 equiv), and DIPEA (2 equiv) in DMF dry (2 mL). The reaction mixture was stirred at room temperature overnight. A control via TLC was performed to ensure the complete consumption of the starting materials. The reaction was quenched with saturated solution of NH_4_Cl, extracted with EtOAc three times. The combined organic layer was dried over Na_2_SO_4_ and concentrated under reduced pressure. The crude product was purified by flash chromatography (MeOH/DCM 5:95) to afford compounds **13**–**15**.

##### 4-(4-(4-(5-(bis­(2-chloroethyl)­amino)-1-methyl-1*H*-benzo­[*d*]­imidazole-2-yl)­butanoyl)­piperazin-1-yl)-3-fluorobenzenesulfonamide (13a)

Following the General Procedure, Bendamustine hydrochloride **1** (52 mg, 0.13 mmol) and **10a** (33 mg, 0.13 mmol) gave **13a** as a white solid. Yield 33%. ^1^H NMR (400 MHz, DMSO-*d*_6_): δ (ppm): 7.58 (2H, ap t, *J* = 9.11 Hz), 7.37 (1H, d, *J* = 8.82 Hz), 7.35 (2H, s, SO_2_N*H*_2_, exchange with D_2_O), 7.20 (1H, t, *J* = 8.71 Hz), 6.95 (1H, d, *J* = 2.01 Hz), 6.83 (1H, dd, *J* = 8.76 Hz; *J* = 2.15 Hz), 3.74 (8H, m), 3.72 (3H, s), 3.66 (4H, m), 3.16 (2H, m), 3.10 (2H, m), 2.90 (2H, t, *J* = 7.33 Hz), 2.56 (2H, m), 2.05 (2H, m); ^13^C NMR (100 MHz, DMSO-*d*_6_): δ (ppm): 171.3, 155.6, 153.0, 144.1, 143.2, 138.1, 130.2, 123.6, 120.1, 114.8, 114.5, 111.1, 110.9, 103.1, 54.4, 50.8, 45.6, 42.3, 32.4, 30.4, 26.9, 23.4; ^19^F NMR (376 MHz, DMSO-*d*_6_): δ (ppm): −120.47; MS (ESI positive) *m*/*z*: 599.17 [M + H]^+^. Elemental Analysis: C, 52.09; H, 5.55; N, 14.02.

##### 4-(4-(4-(5-(bis­(2-chloroethyl)­amino)-1-methyl-1*H*-benzo­[*d*]­imidazole-2-yl)­butanoyl)­piperazin-1-yl)-3,5-difluorobenzenesulfonamide (13b)

Following the General Procedure, Bendamustine hydrochloride **1** (52 mg, 0.13 mmol) and **10b** (35 mg, 0.13 mmol) gave **13b** as a white solid. Yield 42%. ^1^H NMR (400 MHz, DMSO-*d*_6_): δ (ppm): 7.52 (2H, s, SO_2_N*H*_2_, exchange with D_2_O), 7.48 (2H, d, *J* = 8.94 Hz), 7.36 (1H, d, *J* = 8.78 Hz), 6.96 (1H, d, *J* = 2.07 Hz), 6.82 (1H, dd, *J* = 8.76 Hz; *J* = 2.23 Hz), 3.74 (8H, m), 3.72 (3H, s), 3.61 (4H, m), 3.25 (2H, m), 3.18 (2H, m), 2.89 (2H, t, *J* = 7.36 Hz), 2.55 (2H, m), 2.04 (2H, m); ^13^C NMR (100 MHz, DMSO-*d*_6_): δ (ppm): 171.4, 158.2, 155.7, 144.2, 143.2, 139.1, 131.5, 130.2, 111.4, 111.1, 110.8, 103.2, 54.4, 46.8, 42.5, 42.3, 32.5, 30.4, 26.9, 23.4; ^19^F NMR (376 MHz, DMSO-*d*_6_): δ −117.63; MS (ESI positive) *m*/*z*: 617.16 [M + H]^+^. Elemental Analysis: C, 50.57; H, 5.22; N, 13.61.

##### 4-(4-(4-(5-(bis­(2-chloroethyl)­amino)-1-methyl-1*H*-benzo­[*d*]­imidazole-2-yl)­butanoyl)­piperazin-1-yl)-3-(trifluoromethyl)­benzenesulfonamide (13c)

Following the General Procedure, Bendamustine hydrochloride **1** (50 mg, 0.12 mmol) and **10c** (37 mg, 0.12 mmol) gave **13c** as a white solid. Yield 37%. ^1^H NMR (400 MHz, DMSO-*d*_6_): δ (ppm): 8.12 (1H, d, *J* = 1.90 Hz), 8.08 (1H, dd, *J* = 8.51 Hz; *J* = 1.82 Hz), 7.72 (1H, d, *J* = 8.48 Hz), 7.53 (2H, s, SO_2_N*H*_2_, exchange with D_2_O), 7.37 (1H, d, *J* = 8.78 Hz), 6.96 (1H, d, *J* = 2.02 Hz), 6.83 (1H, dd, *J* = 8.79 Hz; *J* = 2.18 Hz), 3.74 (8H, m), 3.72 (3H, s), 3.63 (4H, m), 2.98 (2H, m), 2.93 (2H, m), 2.90 (2H, m), 2.56 (2H, m), 2.05 (2H, m); ^13^C NMR (100 MHz, DMSO-*d*_6_): δ (ppm): 171.4, 155.7, 155.4, 144.2, 143.2, 141.1, 131.8, 130.2, 125.9, 125.7, 123.0, 111.1, 110.8, 103.2, 54.4, 54.0, 53.5, 46.1, 42.3, 32.5, 30.4, 26.9, 23.4; ^19^F NMR (376 MHz, DMSO-*d*_6_): δ −59.01; MS (ESI positive) *m*/*z*: 649.17 [M + H]^+^. Elemental Analysis: C, 49.95; H, 5.13; N, 12.96.

##### 4-(4-(4-(5-(bis­(2-chloroethyl)­amino)-1-methyl-1*H*-benzo­[*d*]­imidazole-2-yl)­butanoyl)­piperazin-1-yl)­benzenesulfonamide (13d)

Following the General Procedure, Bendamustine hydrochloride **1** (55 mg, 0.13 mmol) and **10d** (32 mg, 0.13 mmol) gave **13d** as a white solid. Yield 68%. ^1^H NMR (400 MHz, DMSO-*d*_6_): δ (ppm): 7.66 (2H, d, *J* = 8.85 Hz), 7.36 (1H, d, *J* = 8.78 Hz), 7.10 (2H, s, SO_2_N*H*_2_, exchange with D_2_O), 7.05 (2H, d, *J* = 8.96 Hz), 6.94 (1H, d, *J* = 1.88 Hz), 6.82 (1H, dd, *J* = 8.79 Hz; *J* = 2.10 Hz), 3.73 (8H, m), 3.70 (3H, s), 3.62 (4H, m), 3.28 (2H, m), 3.04 (1H, m), 2.88 (2H, t, *J* = 7.33 Hz), 2.56 (2H, m), 2.03 (2H, m), 1.75 (1H, m); ^13^C NMR (100 MHz, DMSO-*d*_6_): δ (ppm): 171.4, 155.7, 153.4, 144.1, 143.2, 134.0, 130.2, 128.0, 114.8, 111.1, 110.8, 103.1, 54.4, 48.1, 45.2, 42.4, 32.4, 30.4, 26.9, 23.3; MS (ESI positive) *m*/*z*: 581.18 [M + H]^+^. Elemental Analysis: C, 53.73; H, 5.91; N, 14.49.

##### 4-(4-(4-(5-(bis­(2-Chloroethyl)­amino)-1-methyl-1*H*-benzo­[*d*]­imidazole-2-yl)­butanoyl)­piperazin-1-yl)-3-chlorobenzenesulfonamide (13e)

Following the General Procedure, Bendamustine hydrochloride **1** (49 mg, 0.12 mmol) and **10e** (33 mg, 0.12 mmol) gave **13e** as a white solid. Yield 92%. ^1^H NMR (400 MHz, DMSO-*d*_6_): δ (ppm): 7.85 (1H, d, *J* = 2.13 Hz), 7.75 (1H, dd, *J* = 8.47 Hz; *J* = 2.10 Hz), 7.41 (2H, s, SO_2_N*H*_2_, exchange with D_2_O), 7.37 (1H, d, *J* = 8.77 Hz), 7.32 (1H, d, *J* = 8.54 Hz), 6.96 (1H, d, *J* = 2.13 Hz), 6.83 (1H, dd, *J* = 8.76 Hz; *J* = 2.23 Hz), 3.74 (8H, m), 3.72 (3H, s), 3.67 (4H, m), 3.09 (2H, m), 3.05 (4H, m), 2.90 (2H, t, *J* = 7.36 Hz), 2.56 (2H, m), 2.05 (2H, m), 1.77 (2H, m); ^13^C NMR (100 MHz, DMSO-*d*_6_): δ (ppm): 171.4, 155.6, 152.3, 144.1, 143.2, 139.9, 130.2, 128.7, 128.0, 126.6, 122.0, 111.1, 110.9, 103.1, 54.4, 51.7, 51.3, 42.4, 32.5, 30.4, 26.9, 23.4; MS (ESI positive) *m*/*z*: 615.14 [M + H]^+^. Elemental Analysis: C, 50.71; H, 5.41; N, 13.66.

##### 4-(4-(4-(5-(bis­(2-Chloroethyl)­amino)-1-methyl-1*H*-benzo­[*d*]­imidazole-2-yl)­butanoyl)­piperazin-1-yl)-2-fluorobenzenesulfonamide (13f)

Following the General Procedure, Bendamustine hydrochloride **1** (49 mg, 0.12 mmol) and **10f** (31 mg, 0.12 mmol) gave **13f** as a white solid. Yield 61%. ^1^H NMR (400 MHz, DMSO-*d*_6_): δ (ppm): 7.58 (1H, t, *J* = 8.90 Hz), 7.37 (1H, s), 7.36 (2H, s, SO_2_N*H*_2_, exchange with D_2_O), 6.94 (1H, d, *J* = 2.14 Hz), 6.90 (1H, dd, *J* = 14.29 Hz; *J* = 2.15 Hz), 6.82 (2H, dd, *J* = 8.66 Hz; *J* = 1.76 Hz), 3.73 (8H, s), 3.71 (3H, s), 3.61 (4H, m), 3.39 (4H, m), 2.89 (2H, t, *J* = 7.31 Hz), 2.53 (2H, m), 2.04 (2H, m); ^13^C NMR (100 MHz, DMSO-*d*_6_): δ (ppm): 171.4, 159.2, 155.6, 155.4, 155.3, 143.2, 130.3, 130.2, 120.8, 111.1, 110.9, 109.6, 103.1, 102.4, 54.4, 47.6, 47.4, 42.4, 32.4, 30.4, 26.9, 23.3; ^19^F NMR (376 MHz, DMSO-*d*_6_): δ −109.59; MS (ESI positive) *m*/*z*: 599.17 [M + H]^+^. Elemental Analysis: C, 52.11; H, 5.56; N, 14.04.

##### 4-(4-(4-(5-(bis­(2-Chloroethyl)­amino)-1-methyl-1*H*-benzo­[*d*]­imidazole-2-yl)­butanoyl)­piperazin-1-yl)-2-methylbenzenesulfonamide (13g)

Following the General Procedure, Bendamustine hydrochloride **1** (51 mg, 0.12 mmol) and **10g** (32 mg, 0.12 mmol) gave **13g** as a white solid. Yield 55%. ^1^H NMR (400 MHz, DMSO-*d*_6_): δ (ppm): 7.70 (1H, d, *J* = 8.82 Hz), 7.36 (1H, d, *J* = 8.78 Hz), 7.10 (1H, s, SO_2_N*H*_2_, exchange with D_2_O), 6.95 (1H, d, *J* = 2.00 Hz), 6.91 (1H, m), 6.84 (2H, m), 3.74 (8H, s), 3.71 (3H, s), 3.63 (4H, m), 3.28 (4H, m), 2.89 (2H, t, *J* = 7.34 Hz), 2.56 (3H, s), 2.05 (2H, m); ^13^C NMR (100 MHz, DMSO-*d*_6_): δ (ppm): 171.4, 155.7, 153.3, 144.3, 143.2, 138.1, 132.4, 130.3, 129.8, 118.1, 111.8, 111.1, 110.8, 103.2, 54.4, 48.1, 47.8, 42.4, 32.4, 30.3, 26.9, 23.3, 21.2; MS (ESI positive) *m*/*z*: 595.19 [M + H]^+^. Elemental Analysis: C, 54.47; H, 6.11; N, 14.12.

##### 4-(4-(4-(5-(bis­(2-Chloroethyl)­amino)-1-methyl-1H-benzo­[*d*]­imidazole-2-yl)­butanoyl)­piperazin-1-yl)-3-cyanobenzenesulfonamide (13h)

Following the General Procedure, Bendamustine hydrochloride **1** (51 mg, 0.12 mmol) and **10h** (34 mg, 0.12 mmol) gave **13h** as a yellow solid. Yield 36%. ^1^H NMR (400 MHz, DMSO-*d*_6_): δ (ppm): 8.07 (1H, d, *J* = 2.27 Hz), 7.97 (1H, dd, *J* = 8.86 Hz; *J* = 2.27 Hz), 7.44 (2H, s, SO_2_N*H*_2_, exchange with D_2_O), 7.38 (1H, d, *J* = 8.80 Hz), 7.32 (1H, d, *J* = 8.93 Hz), 6.96 (1H, d, *J* = 2.10 Hz), 6.83 (1H, dd, *J* = 8.81 Hz; *J* = 2.26 Hz), 3.74 (8H, s), 3.72 (3H, s), 3.69 (4H, m), 3.40 (2H, m), 3.32 (2H, m), 2.90 (2H, t, *J* = 7.35 Hz), 2.57 (2H, m), 2.05 (2H, m); ^13^C NMR (100 MHz, DMSO-*d*_6_): δ (ppm): 171.5, 157.3, 155.6, 144.0, 143.2, 137.2, 133.0, 132.3, 130.1, 120.0, 118.4, 111.1, 110.9, 103.3, 103.0, 54.4, 51.5, 51.0, 42.3, 32.5, 30.4, 26.9, 23.3; MS (ESI positive) *m*/*z*: 606.17 [M + H]^+^. Elemental Analysis: C, 53.46; H, 5.48; N, 16.16.

##### 4-(4-(4-(5-(bis­(2-Chloroethyl)­amino)-1-methyl-1*H*-benzo­[*d*]­imidazole-2-yl)­butanoyl)-1,4-diazepan-1-yl)­benzenesulfonamide (14a)

Following the General Procedure, Bendamustine hydrochloride **1** (50 mg, 0.12 mmol) and **11a** (31 mg, 0.12 mmol) gave **14a** as a white solid. Yield 47%. ^1^H NMR (400 MHz, DMSO-*d*_6_): δ (ppm): 7.61 (2H, dd, *J* = 8.95 Hz; *J* = 1.60 Hz), 7.36 (1H, dd, *J* = 8.78 Hz; *J* = 3.72 Hz), 7.10 (1H, s), 7.01 (1H, s), 6.95 (1H, dd, *J* = 5.42 Hz; *J* = 2.11 Hz), 6.87 (2H, dd, *J* = 8.93 Hz; *J* = 6.36 Hz), 6.83 (1H, dt, *J* = 8.80 Hz; *J* = 2.47 Hz), 3.74 (8H, s), 3.67 (3H, d, *J* = 9.45 Hz), 3.63 (4H, m), 3.37 (4H, m), 2.81 (1H, t, *J* = 7.35 Hz), 2.72 (1H, t, *J* = 7.42 Hz), 2.48 (1H, t, *J* = 6.95 Hz), 2.36 (1H, t, *J* = 6.94 Hz), 1.98 (1H, m), 1.86 (3H, m); ^13^C NMR (100 MHz, DMSO-*d*_6_): δ (ppm): 172.2, 171.9, 155.7, 150.3, 143.2, 131.1, 130.2, 128.5, 111.5, 111.1, 110.8, 103.1, 54.4, 50.5, 49.2, 48.1, 45.3, 45.0, 42.4, 32.2, 30.3, 26.8, 23.4; MS (ESI positive) *m*/*z*: 595.19 [M + H]^+^. Elemental Analysis: C, 54.47; H, 6.10; N, 14.11.

##### 4-(4-(4-(5-(bis­(2-Chloroethyl)­amino)-1-methyl-1*H*-benzo­[*d*]­imidazole-2-yl)­butanoyl)-1,4-diazepan-1-yl)-3,5-difluorobenzenesulfonamide (14b)

Following the General Procedure, Bendamustine hydrochloride **1** (51 mg, 0.13 mmol) and **11b** (37 mg, 0.13 mmol) gave **14b** as a white solid. Yield 57%. ^1^H NMR (400 MHz, DMSO-*d*_6_): δ (ppm): 7.49 (2H, d, *J* = 5.30 Hz), 7.44 (2H, d), 7.37 (1H, dd, *J* = 8.74 Hz; *J* = 4.21 Hz), 6.95 (1H, t, *J* = 2.31 Hz), 6.84 (1H, d, *J* = 8.42 Hz), 3.75 (8H, m), 3.71 (3H, d, *J* = 2.35 Hz), 3.63 (4H, m), 3.51 (2H, m), 3.46 (2H, m), 2.88 (2H, m), 2.51 (2H, m), 2.03 (2H, m), 1.86 (2H, m); ^13^C NMR (100 MHz, DMSO-*d*_6_): δ (ppm): 172.2, 172.1, 157.9, 155.6, 155.5, 143.3, 130.1, 111.4, 111.2, 111.1, 110.9, 103.0, 54.4, 47.4, 47.1, 46.8, 46.7, 44.9, 42.3, 32.3, 30.4, 26.8, 23.4; ^19^F NMR (376 MHz, DMSO-*d*_6_): δ −116.70; MS (ESI positive) *m*/*z*: 631.18 [M + H]^+^. Elemental Analysis: C, 51.35; H, 5.43; N, 13.31.

##### 4-(4-(4-(5-(bis­(2-Chloroethyl)­amino)-1-methyl-1*H*-benzo­[*d*]­imidazole-2-yl)­butanoyl)-1,4-diazepan-1-yl)-3-fluorobenzenesulfonamide (14c)

Following the General Procedure, Bendamustine hydrochloride **1** (51 mg, 0.12 mmol) and **11c** (34 mg, 0.12 mmol) gave **14c** as a white solid. Yield 68%. ^1^H NMR (400 MHz, DMSO-*d*_6_): δ (ppm): 7.47 (2H, d, *J* = 13.08 Hz), 7.35 (1H, d, *J* = 8.75 Hz), 7.30 (1H, s), 7.23 (1H, s), 7.08 (1H, t, *J* = 8.82 Hz), 6.95 (1H, s), 6.82 (1H, d, *J* = 8.81 Hz), 3.74 (8H, m), 3.68 (3H, s), 3.66 (2H, m), 3.55 (4H, m), 3.05 (1H, m), 2.88 (2H, m), 2.51 (2H, m), 2.03 (2H, m), 1.86 (2H, m); ^13^C NMR (100 MHz, DMSO-*d*_6_): δ (ppm): 172.2, 171.9, 155.7, 143.2, 143.1, 130.2, 123.8, 117.5, 115.5, 115.4, 115.1, 111.0, 110.8, 103.2, 54.4, 47.1, 46.8, 46.7, 46.1, 44.7, 42.3, 32.2, 30.3, 26.8, 23.4; ^19^F NMR (376 MHz, DMSO-*d*_6_): δ −123.41; MS (ESI positive) *m*/*z*: 613.19 [M + H]^+^. Elemental Analysis: C, 52.85; H, 5.75; N, 13.70.

##### 4-(4-(4-(5-(bis­(2-Chloroethyl)­amino)-1-methyl-1*H*-benzo­[*d*]­imidazole-2-yl)­butanoyl)-1,4-diazepan-1-yl)-3-chlorobenzenesulfonamide (14d)

Following the General Procedure, Bendamustine hydrochloride **1** (50 mg, 0.12 mmol) and **11d** (35 mg, 0.12 mmol) gave **14d** as a white solid. Yield 63%. ^1^H NMR (400 MHz, DMSO-*d*_6_): δ (ppm): 7.80 (1H, d, *J* = 2.13 Hz), 7.67 (1H, dt, *J* = 8.64 Hz; *J* = 1.52 Hz), 7.38 (1H, d, *J* = 6.16 Hz), 7.36 (2H, s, SO_2_N*H*_2_, exchange with D_2_O), 7.33 (1H, dd, *J* = 13.24 Hz; *J* = 4.95 Hz), 6.95 (1H, d, *J* = 2.04 Hz), 6.82 (1H, dd, *J* = 8.76 Hz; *J* = 1.99 Hz), 3.74 (8H, m), 3.70 (3H, s), 3.63 (2H, m), 3.40 (2H, m), 3.31 (4H, m), 2.87 (2H, dd, *J* = 16.98 Hz; *J* = 7.51 Hz), 2.55 (2H, m), 2.04 (2H, m); ^13^C NMR (100 MHz, DMSO-*d*_6_): δ (ppm): 172.3, 172.1, 155.7, 153.5, 143.2, 138.4, 130.2, 129.0, 126.5, 126.3, 122.1, 111.1, 110.8, 103.2, 56.8, 54.4, 53.6, 47.0, 42.3, 32.3, 30.3, 29.5, 28.2, 26.9, 23.4; MS (ESI positive) *m*/*z*: 629.16 [M + H]^+^. Elemental Analysis: C, 51.49; H, 5.61; N, 13.36.

##### 4-(3-(4-(4-(5-(bis­(2-Chloroethyl)­amino)-1-methyl-1*H*-benzo­[*d*]­imidazole-2-yl)­butanoyl)­piperazin-1-yl)-3-oxopropyl)­benzenesulfonamide (15)

Following the General Procedure, Bendamustine hydrochloride **1** (61 mg, 0.15 mmol) and **12** (50 mg, 0.15 mmol) gave **15** as a white solid. Yield 37%. ^1^H NMR (400 MHz, DMSO-*d*_6_): δ (ppm): 7.76 (2H, d, *J* = 8.17 Hz), 7.47 (2H, d, *J* = 8.20 Hz), 7.39 (1H, d, *J* = 8.73 Hz), 7.32 (2H, s, SO_2_N*H*_2_, exchange with D_2_O), 6.95 (1H, d, *J* = 1.87 Hz), 6.85 (1H, d, *J* = 9.40 Hz), 3.75 (8H, s), 3.72 (3H, s), 3.46 (10H, m), 2.91 (4H, m), 2.73 (2H, t, *J* = 7.06 Hz), 2.51 (2H, m); ^13^C NMR (100 MHz, DMSO-*d*_6_): δ (ppm): 171.4, 170.7, 155.5, 146.9, 146.6, 143.4, 142.8, 133.4, 129.8, 126.5, 111.3, 111.0, 102.6, 54.3, 45.7, 45.4, 42.3, 34.4, 32.4, 31.2, 30.4, 26.8, 23.2; MS (ESI positive) *m*/*z*: 637.21 [M + H]^+^. Elemental Analysis: C, 54.63; H, 6.01; N, 13.18.

#### General Procedure for the Synthesis of Compounds 10a–h and 11a–d

In a flask, the appropriate 4-fluorobenzenesulfonamide (**16a**–**h**) (1.0 equiv) and piperazine (**17**) or homopiperazine (**18**) (3.0 equiv) were suspended in H_2_O (50 mL). The reaction mixture was stirred at reflux temperature overnight. A control via TLC was performed to ensure the complete consumption of the starting materials. The reaction was cooled to room temperature and a precipitate was formed, filtered off under vacuum, washed with H_2_O and Et_2_O and dried on air. No further purification was needed. Obtained intermediates were in agreement as previously reported.^33,42^

#### 2-Fluoro-4-(piperazin-1-yl)­benzenesulfonamide (10f)

Following the General Procedure, 2,4-difluorobenzenesulfonamide **16f** (500 mg, 2.6 mmol) and piperazine **17** (670 mg, 7.8 mmol) gave as a white solid. Yield 68%. ^1^H NMR (400 MHz, DMSO-*d*_6_): δ (ppm): 7.56 (1H, t, *J* = 8.94 Hz) 7.32 (2H, s, SO_2_N*H*_2_, exchange with D_2_O), 6.86 (1H, d, *J* = 14.62 Hz), 6.80 (1H, d, *J* = 8.82 Hz), 3.24 (4H, m), 2.82 (4H, m); MS (ESI positive) *m*/*z*: 260.08 [M + H]^+^.

#### 2-Methyl-4-(piperazin-1-yl)­benzenesulfonamide (10g)

Following the General Procedure, 4-fluoro-2-methylbenzenesulfonamide **16g** (500 mg, 2.6 mmol) and piperazine **17** (670 mg, 7.8 mmol) gave as a white solid. Yield 72%. ^1^H NMR (400 MHz, DMSO-*d*_6_): δ (ppm): 7.67 (1H, dd, *J* = 8.69 Hz; *J* = 4.83 Hz), 7.07 (2H, s, SO_2_N*H*_2_, exchange with D_2_O), 6.85 (1H, s), 6.80 (1H, d, *J* = 8.85 Hz), 3.17 (4H, m), 2.82 (4H, m), 2.55 (3H, s), 2.39 (1H, m, N*H*, exchange with D_2_O); ^13^C NMR (100 MHz, DMSO-*d*_6_): δ (ppm): 154.2, 138.0, 131.8, 129.8, 117.8, 111.4, 49.0, 46.3, 21.3; MS (ESI positive) *m*/*z*: 256.10 [M + H]^+^.

#### 3-Cyano-4-(piperazin-1-yl)­benzenesulfonamide (10h)

Following the General Procedure, 3-cyano-4-fluorobenzenesulfonamide **16h** (600 mg, 3.0 mmol) and piperazine **17** (775 mg, 9.0 mmol) gave as a white solid. Yield 81%. ^1^H NMR (400 MHz, DMSO-*d*_6_): δ (ppm): 8.03 (1H, m, N*H*, exchange with D_2_O), 7.94 (1H, dd, *J* = 8.86 Hz; *J* = 2.14 Hz), 7.40 (2H, s, SO_2_N*H*_2_, exchange with D_2_O), 7.29 (2H, d, *J* = 8.90 Hz), 3.27 (4H, m), 2.89 (4H, m); ^13^C NMR (100 MHz, DMSO-*d*_6_): δ (ppm): 158.1, 136.6, 133.1, 132.3, 119.8, 118.6, 103.1, 52.8, 46.4; MS (ESI positive) *m*/*z*: 267.08 [M + H]^+^.

#### Synthesis of Compound *tert*-butyl 4-(3-(4-Sulfamoylphenyl)­propanoyl)­piperazine-1-carboxylate (21)

Under an inert atmosphere (N_2_), 1-Boc piperazine **19** (879 mg, 4.72 mmol, 1.1 equiv) was added to a solution of 3-(4-sulfamoylphenyl)­propanoic acid **20** (984 mg, 4.29 mmol, 1 equiv), PyBOP (1.5 equiv), and DIPEA (2 equiv) in DMF dry (2 mL). The reaction mixture was stirred at room temperature overnight. A control via TLC was performed to ensure the complete consumption of the starting materials. The reaction was quenched with saturated solution of NH_4_Cl and a white precipitate was formed, filtered off under vacuum, washed with H_2_O and Et_2_O and dried on air. No further purification was needed. White solid. Yield 84%. ^1^H NMR (400 MHz, DMSO-*d*_6_): δ (ppm): 7.76 (2H, d, *J* = 8.22 Hz), 7.47 (2H, d, *J* = 8.20 Hz), 7.29 (2H, s, SO_2_N*H*_2_, exchange with D_2_O), 3.46 (4H, m), 3.33 (4H, m), 2.92 (2H, d, *J* = 7.40 Hz), 2.71 (2H, t, *J* = 7.56 Hz), 1.44 (9H, s); MS (ESI positive) *m*/*z*: 398.17 [M + H]^+^.

#### Synthesis of Compound 4-(3-oxo-3-(Piperazin-1-yl)­propyl)­benzenesulfonamide Hydrochloride (12)

Under an inert atmosphere (N_2_), 3-(4-sulfamoylphenyl)­propanoic acid **20** (850 mg, 2.14 mmol, 1 equiv) was dissolved in trifluoroacetic acid (2.5 mL, 10 equiv). The reaction mixture was stirred at room temperature overnight. A control via TLC was performed to ensure the complete consumption of the starting materials. The reaction was quenched removing the solvent under reduced pressure. The afforded product **12** was pure as it is, no further purification was needed. White solid. Yield 88%. ^1^H NMR (400 MHz, DMSO-*d*_6_): δ (ppm): 9.65 (2H, s, N*H*_2_, exchange with D_2_O), 7.97 (2H, s, SO_2_N*H*_2_, exchange with D_2_O), 7.76 (2H, d, *J* = 8.24 Hz), 7.46 (2H, d, *J* = 8.22 Hz), 3.72 (4H, m), 3.03 (4H, m), 2.89 (2H, s), 2.75 (2H, t, *J* = 7.56 Hz); MS (ESI negative) *m*/*z*: 332.09 [M-H]^−^.

### Carbonic Anhydrase Inhibition

An Applied Photophysics stopped-flow instrument was used to assay the CA catalyzed CO_2_ hydration activity.^34^ Phenol red (at a concentration of 0.2 mM) was used as an indicator, working at the absorbance maximum of 557 nm, with 20 mM Hepes (pH 7.4) as a buffer, and 20 mM Na_2_SO_4_ (to maintain constant ionic strength), following the initial rates of the CA-catalyzed CO_2_ hydration reaction for a period of 10–100 s. The CO_2_ concentrations ranged from 1.7 to 17 mM for the determination of the kinetic parameters and inhibition constants.^43^ Enzyme concentrations ranged between 5 and 12 nM. For each inhibitor, at least six traces of the initial 5–10% of the reaction were used to determine the initial velocity. The uncatalyzed rates were determined in the same manner and subtracted from the total observed rates. Stock solutions of the inhibitor (0.1 mM) were prepared in distilled–deionized water and dilutions up to 0.01 nM were done thereafter with the assay buffer. Inhibitor and enzyme solutions were preincubated together for 15 min at room temperature prior to the assay, to allow for the formation of the E–I complex. The inhibition constants were obtained by nonlinear least-squares methods using PRISM 3 and the Cheng-Prusoff equation as reported earlier and represent the mean from at least three different determinations. All CA isoforms were recombinant proteins obtained in house, as reported earlier.^44–46^

### Crystallization and X-ray Data Collection

Crystals of hCAII were obtained using the hanging drop vapor diffusion method using 24 well Linbro plate. Two μL of 10 mg/mL solution of hCA II in Tris–HCl 20 mM pH 8.0 were mixed with 2 μL of a solution of 1.5 M sodium citrate, 0.1 M Tris pH 8.0 and were equilibrated against the same solution at 296 K. The complexes were prepared by soaking the hCA II native crystals in the mother liquor solution containing the inhibitors at concentration of 10 mM for 2 days. All crystals were flash-frozen at 100 K using a solution obtained by adding 15% (v/v) glycerol to the mother liquor solution as cryoprotectant. Data on crystals of the complexes were collected using synchrotron radiation at the XRD2 beamline at Elettra Synchrotron (Trieste, Italy) with a wavelength of 1.000 Å and a DECTRIS Pilatus 6 M detector. Data were integrated and scaled using the program XDS.^47^ Data processing statistics are shown in Supporting Information.

#### Structure Determination

The crystal structure of hCA II (PDB accession code: 4FIK) without solvent molecules and other heteroatoms was used to obtain initial phases using Refmac5.^48^ 5% of the unique reflections were selected randomly and excluded from the refinement data set for the purpose of Rfree calculations. The initial |Fo - Fc| difference electron density maps unambiguously showed the inhibitor molecules. The inhibitor was introduced in the model with 1.0 occupancy. Refinements proceeded using normal protocols of positional, isotropic atomic displacement parameters alternating with manual building of the models using COOT.^49^ The quality of the final models was assessed with COOT and RAMPAGE.^50^ Crystal parameters and refinement data are summarized in Electronic Supporting Information (ESI). Atomic coordinates were deposited in the Protein Data Bank (PDB accession code: 30NN). Graphical representations were generated with Chimera.^51^

#### Cell Lines and Culture

RCC cell lines 786-O (VHL-mutated) and CAKI-1 (VHL Wild-Type) were obtained from ATCC (Manassas, VA, USA; www.lgcstandards-atcc.org). 786-O and CAKI-1 cell lines were cultured, respectively, in Roswell Park Memorial Institute (RPMI)-1640 or McCoy’s 5A medium (Life technologies, Carlsbad, CA, USA) supplemented with 10% fetal bovine serum, l-glutamine (2 mM), penicillin/streptomycin (50 U/ml) (Euroclone, Milan, Italy) at 37 °C and 5% CO_2_. Mycoplasma was periodically tested by 4′,6-diamidino-2-phenylindole (DAPI) inspection and PCR upon thawing of a new batch of cells and once a month. Cultures are renewed every 2 months.

#### Cell Viability Assay

Cell viability was measured using Prestoblue Cell Viability reagent (Invitrogen, Waltham, MA, USA according to the manufacturer’s protocol. The optical density (OD) was measured using a 560 nm excitation filter and 590 nm emission filter using the BioTek Synergy H1 hybrid multimode microplate reader (Agilent, CA, USA). Half-maximal inhibitory concentration (IC_50_) values were derived by a sigmoidal dose-response (variable slope) curve fitted using a four-parameter logistic regression model (log­(inhibitor) vs normalized response Variable slope (four parameters) as described in the software documentation of Graph Pad Prism v6.0.

### Cell Lysis and Western Blotting

Total cell lysates were obtained using Laemmli buffer (62.5 mM Tris–HCl pH 6.8, 10% glycerol, 0.005% bromophenol blue, SDS 2%). Culture plates were placed on ice and cell monolayers were rapidly washed three times with ice-cold PBS containing 100 mM orthovanadate (Merck Millipore, Billerica, MA, USA). Cells were lysed by scraping in Laemmli buffer and incubating at 95 °C for 10 min. Lysates were then clarified by centrifugation (13000 rpm for 10 min at room temperature). Proteins were separated on Bolt BisTris Plus gels 4–12% precast polyacrylamide gels (Life Technologies, Monza, Italy). Then, proteins were transferred from the gel to 0.2 μm nitrocellulose membranes using the Trans-Blot Turbo Transfer Starter System (BioRad, Hercules, CA, USA). Blots were blocked for 5 min, at room temperature, with the EveryBlot Blocking Buffer (BioRad, Hercules, CA, USA). Subsequently, the membrane was probed at 4 °C overnight with primary antibodies diluted in a solution of 1:1 Immobilon Block-FL/T-PBS buffer (Merck Millipore, Billerica, MA, USA). The primary antibodies were as follows: mouse anti-Vinculin, Rabbit anti-p21Waf1/Cip1, rabbit anti-CA9 (1:1000, Cell Signaling Technology, Danvers, MA, USA) and rabbit anticleaved caspase 3 (MedChemExpress, Monmouth Junction, NJ, USA).

### Analysis of Cell Cycle

A total of 200 000 cells/well were seeded in 6-multiwell plates. After medium removal, 500 μL of solution containing 50 μg/mL propidium iodide, 0.1% w/v trisodium citrate and 0.1% NP40 was added (Sigma-Aldrich, St. Louis, MO, USA). Samples were then incubated for 1 h at 4 °C in the dark and nuclei analyzed with FACSCanto flow cytometer (Becton Dickinson, Franklin Lakes, NJ, USA).

#### 2D Clonogenic Assay

For colony formation assay, 2000 (786-O) or 2500 (CAKI-1) cells were seeded in 6-multiwell plates in the presence of drugs or vehicle (DMSO). Colonies (with more than 50 cells, i.e., 8 cell diameter) were counted following crystal violet staining after 7 (786-O) or 10 (CAKI-1) days.

#### Spheroid Formation Assay

786-O and CAKI-1 cells were seeded, respectively, in RPMI and McCoy’s 5A 10% FBS in 96-well plate (2000 cells/well) precoated with 1.5% agarose (Condalab, Madrid, Spain) in water. After 72 h, photos of time 0 were taken and spheroids were left untreated (CTRL) or treated with drugs. Photos were taken after 3 and 7 days of treatment by using Leica DM1 Inverted Microscope (Leica, Wetzlar, Germany) and the volume of SW1573-PR/GR or H23-PR/GR spheroids was quantified with ImageJ [Volume = 0.5*L*W2, *L* = length (major axis) *W* = width (minor axis)].

#### Statistical Analysis

Data represent mean or ±SD values calculated on at least three independent experiments. P-values were calculated using the appropriate statistical test based on the distribution of the data and multiple testing corrections were applied when necessary, using the Bonferroni method.

## Supplementary Material





## Notes

^1^H, ^13^C, ^19^F NMR spectra of compounds. Summary of Data Collection and Atomic Model Refinement Statistics for hCA II. Figure S1, Figure S2 (PDF)
Molecular Formula Strings file (CSV)

## Abbreviations

hCA: human carbonic anhydrase
ccRCC: Clear cell renal cell carcinoma
VHL: von Hippel-Lindau
HIF: hypoxia-inducible factor 1-alpha
CAI: carbonic anhydrase inhibitor
AAZ: acetazolamide
TLC: thin layer chromatography
