# Supplementary material for: Design and Synthesis of Bendamustine-Carbonic Anhydrase Inhibitors with Antiproliferative Effects in Clear Cell Renal Cell Carcinoma
Source: J Med Chem. 2026 Jun 24;69(13):16082–97. doi: 10.1021/acs.jmedchem.6c01549 (PMC13370868; doi:10.1021/acs.jmedchem.6c01549)
Supplement: Supplementary file 1 [file jm6c01549_si_001.pdf]

Supplementary Material for

Design and Synthesis of Bendamustine-Carbonic Anhydrase Inhibitors with Antiproliferative Effects in Clear Cell Renal Cell Carcinoma

Gioele Renzi[1], Alessandro Tubita[2], Lorenzo Antonuzzo[2], Serena Pillozzi[2], Marta Ferraroni[4], Andrea Angeli[1], Claudiu T. Supuran[1]*

[1] NEUROFARBA Department, Sezione di Scienze Farmaceutiche, University of Florence, Via Ugo Schiff 6, 50019, Sesto Fiorentino, Florence, Italy

[2] Department of Experimental and Clinical Biomedical Sciences "Mario Serio", University of Florence, Viale Morgagni, 50, 50134, Florence, Italy

[3] Department of Chemistry "Ugo Schiff", University of Florence, Via della Lastruccia 3-13, 50019, Sesto Fiorentino, Italy

KEYWORDS Carbonic anhydrase, bendamustine, metalloenzyme, tumor, Renal cell carcinoma

*Corresponding authors: (A.A), mail: andrea.angeli@unifi.it

Index

^{1}H, ^{13}C, ^{19}F Spectra of final compounds *S2-32*

Figure S1-S3 *S-33-34*

Summary of Data Collection and Atomic Model Refinement Statistics for hCA II *S35*

Copies of NMR Spectra of final compounds

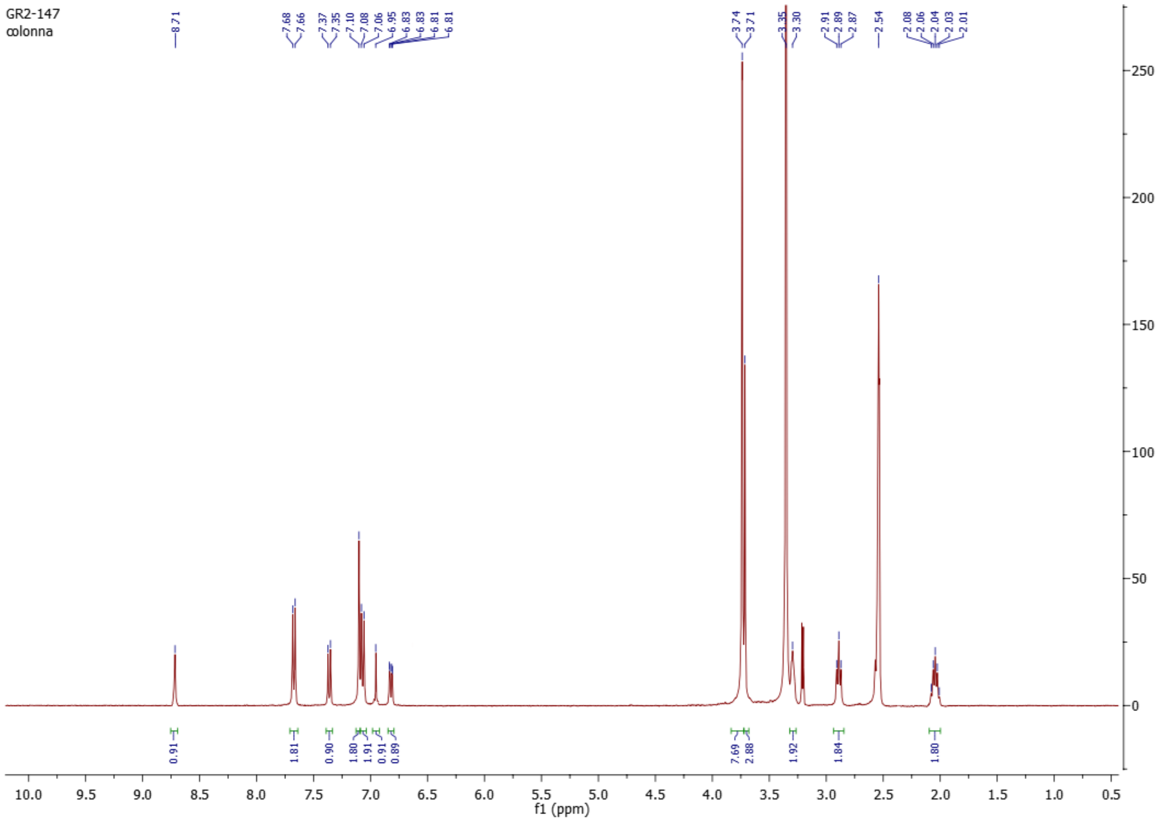

^{1}H NMR spectrum of compound **6a** (400 MHz, DMSO-d_6)

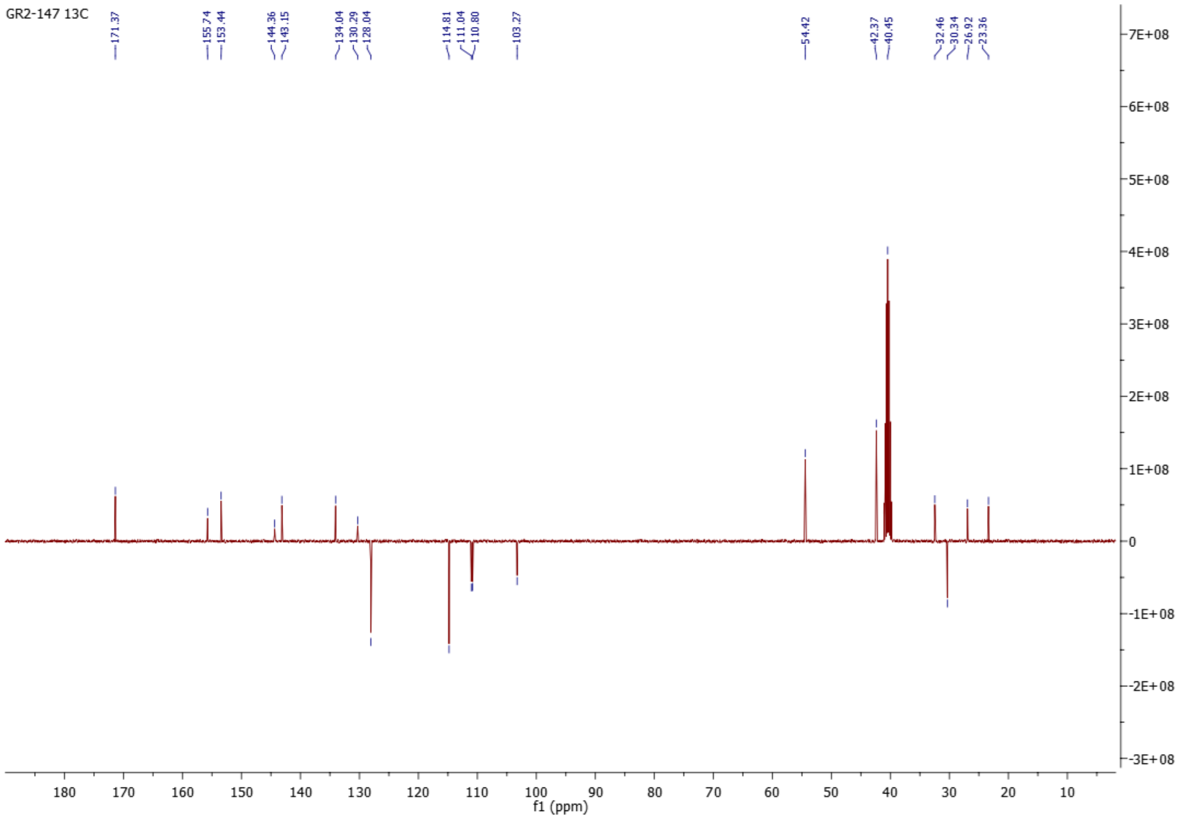

^{13}C NMR spectrum of compound **6a** (100 MHz, DMSO-d_6)

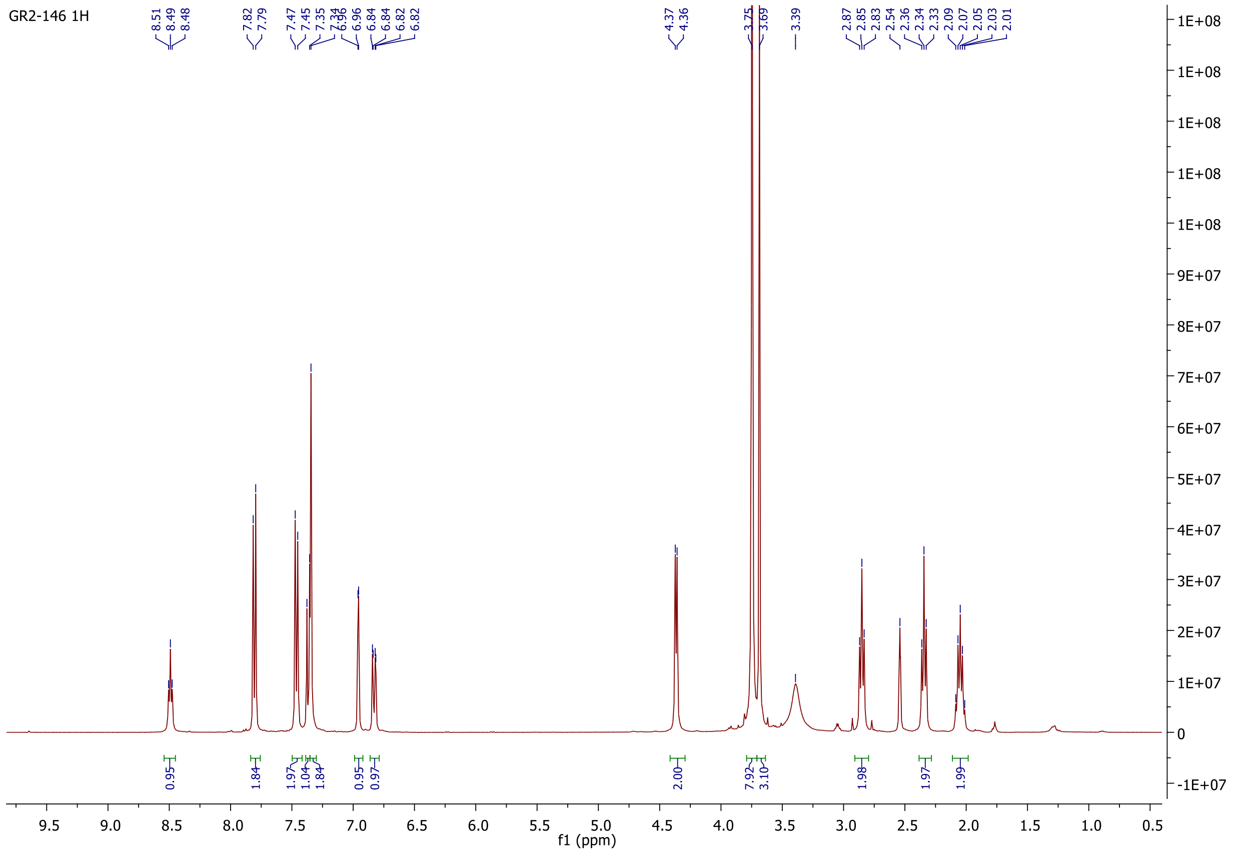

^{1}H NMR spectrum of compound **6b** (400 MHz, DMSO-d_6)

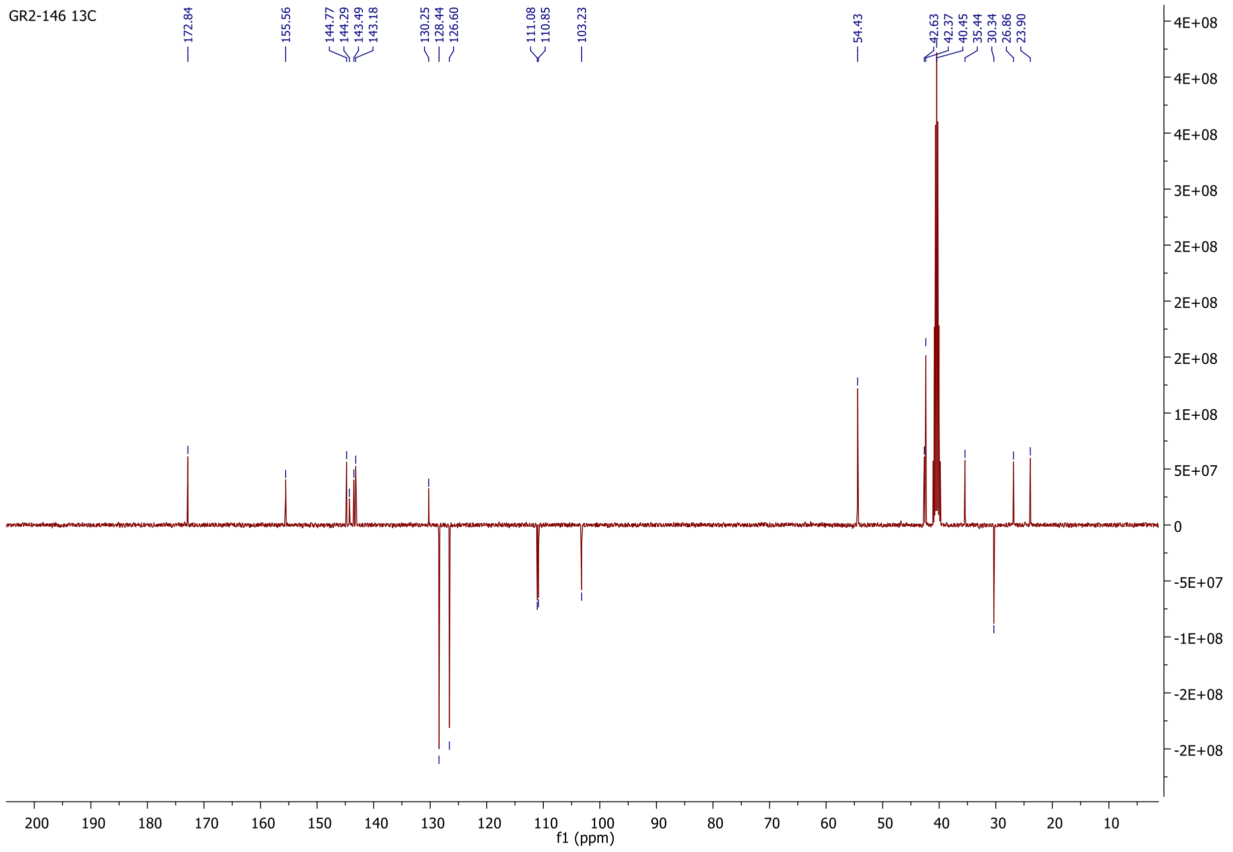

^{13}C NMR spectrum of compound **6b** (100 MHz, DMSO-d_6)

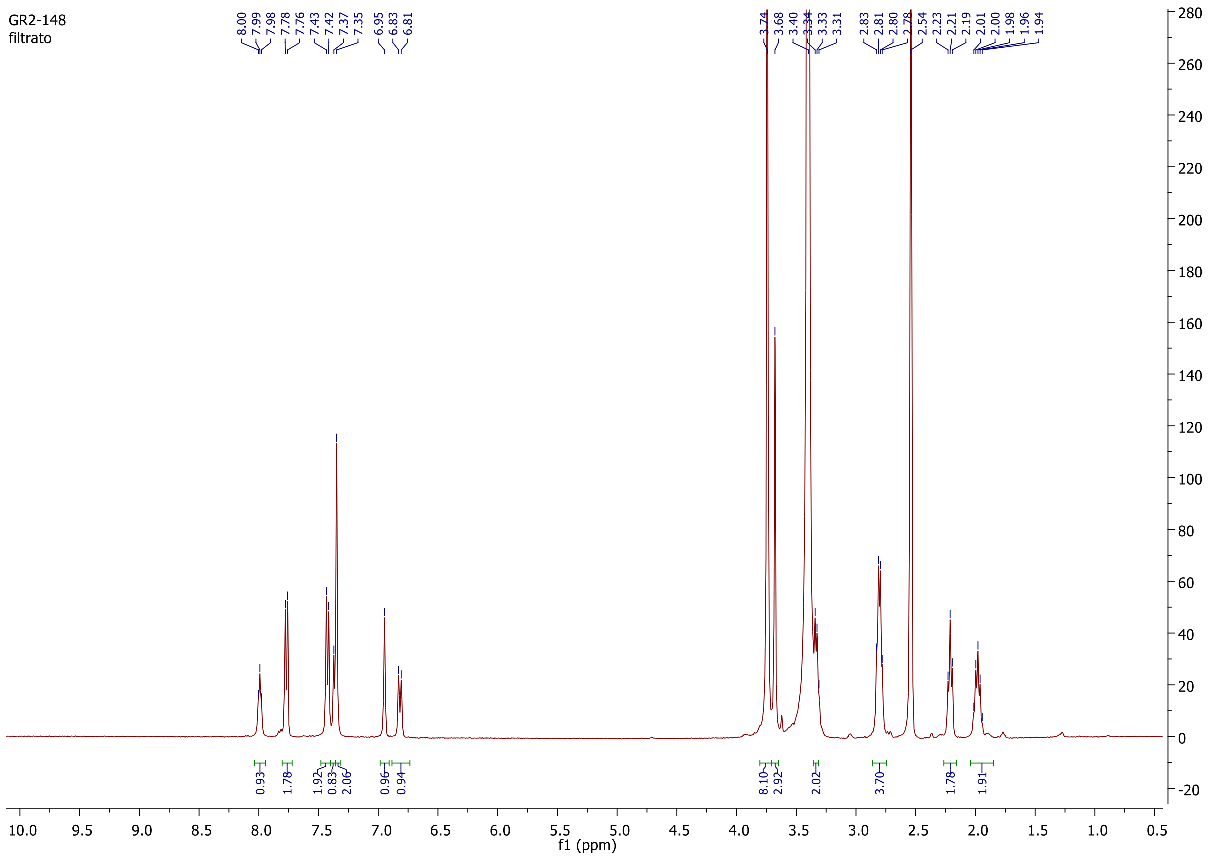

^{1}H NMR spectrum of compound **6c** (400 MHz, DMSO-d_6)

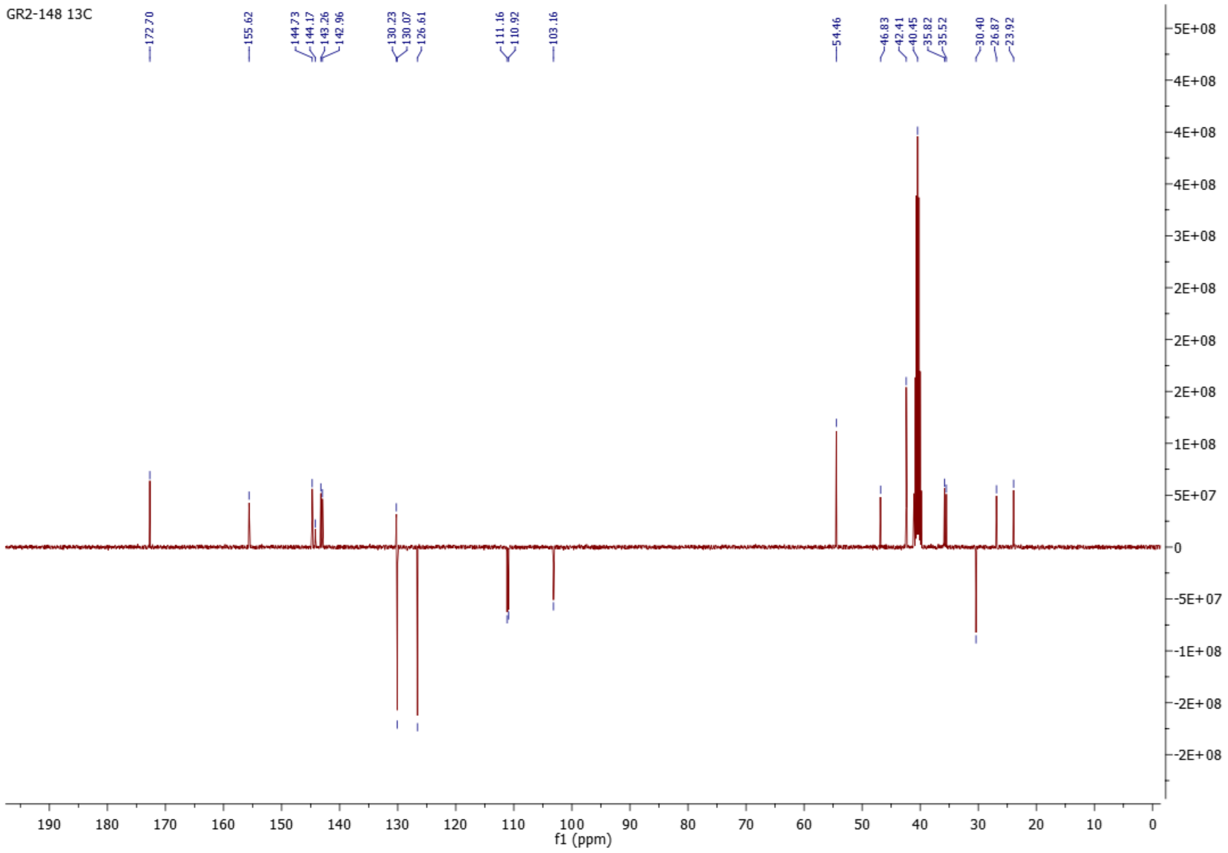

^{13}C NMR spectrum of compound **6c** (100 MHz, DMSO-d_6)

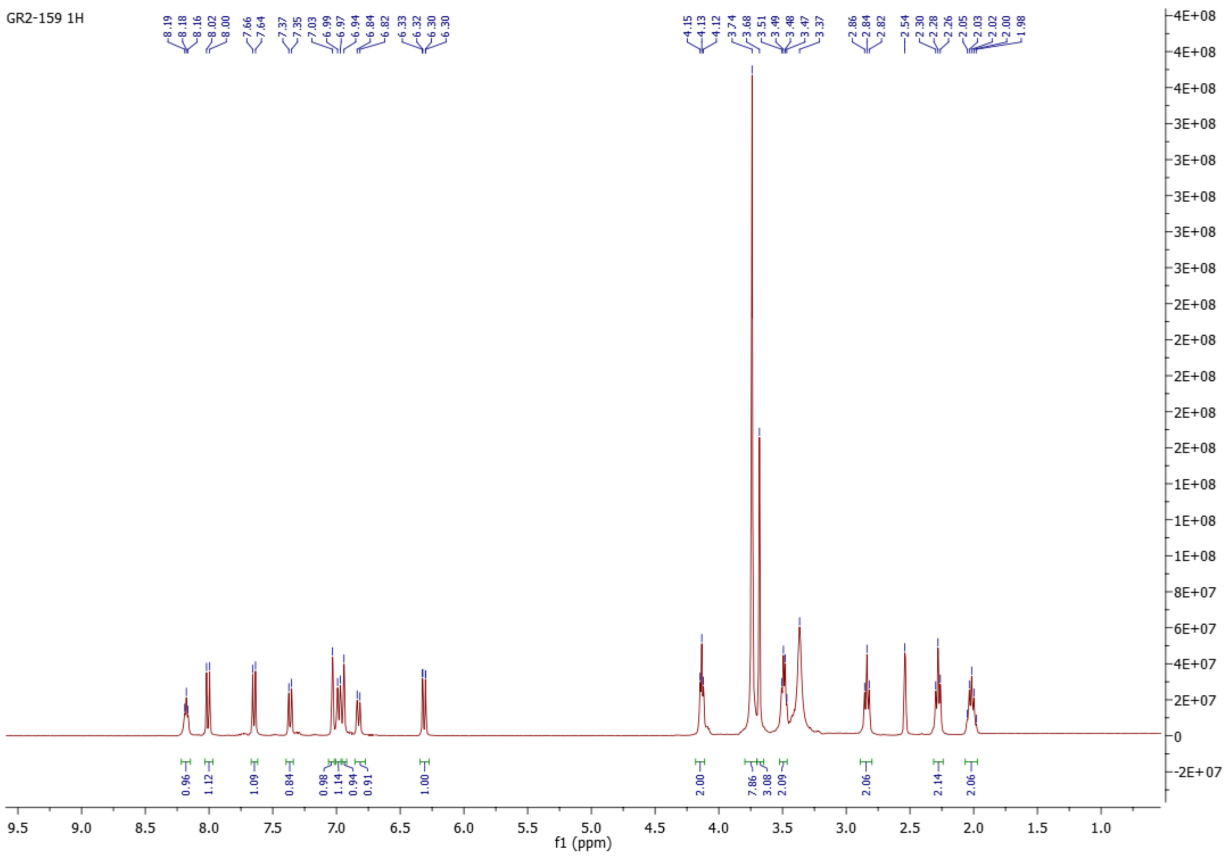

^{1}H NMR spectrum of compound **7a** (400 MHz, DMSO-d_6)

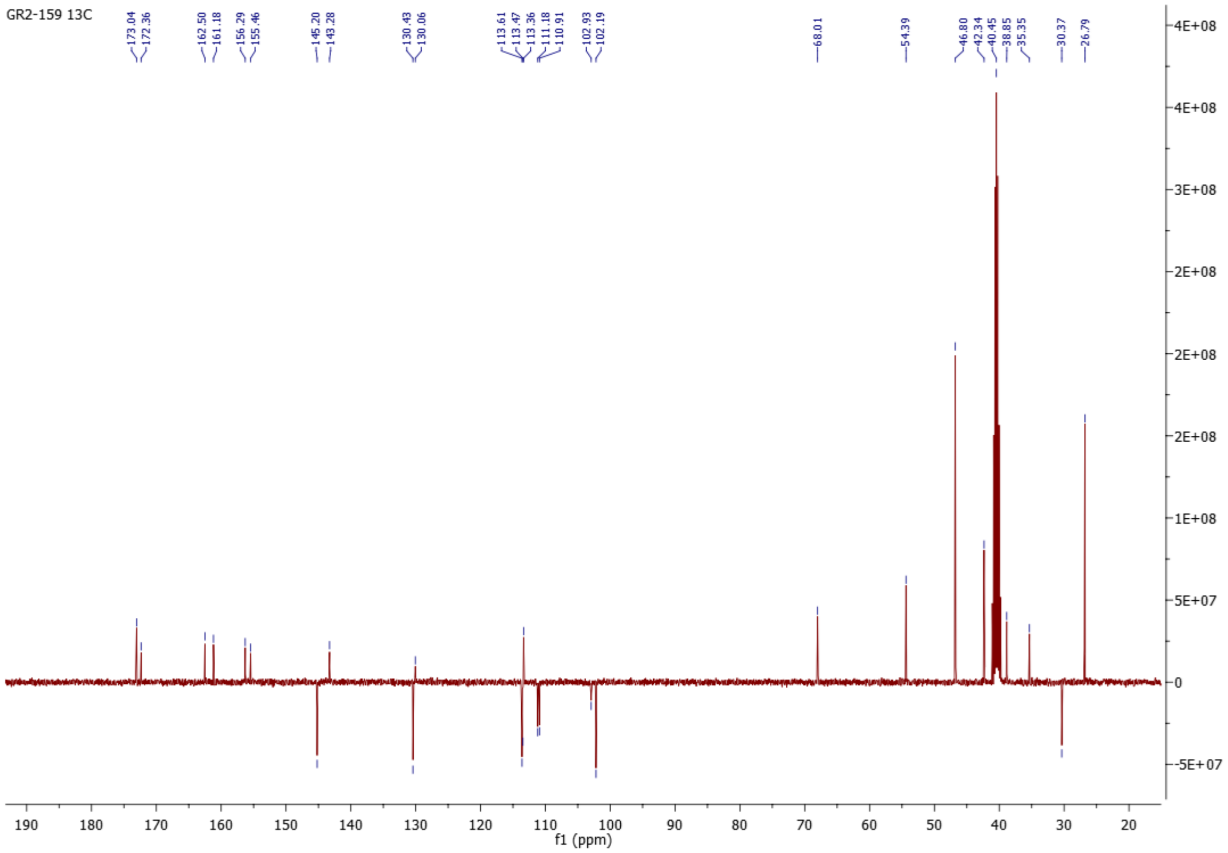

^{13}C NMR spectrum of compound **7a** (100 MHz, DMSO-d_6)

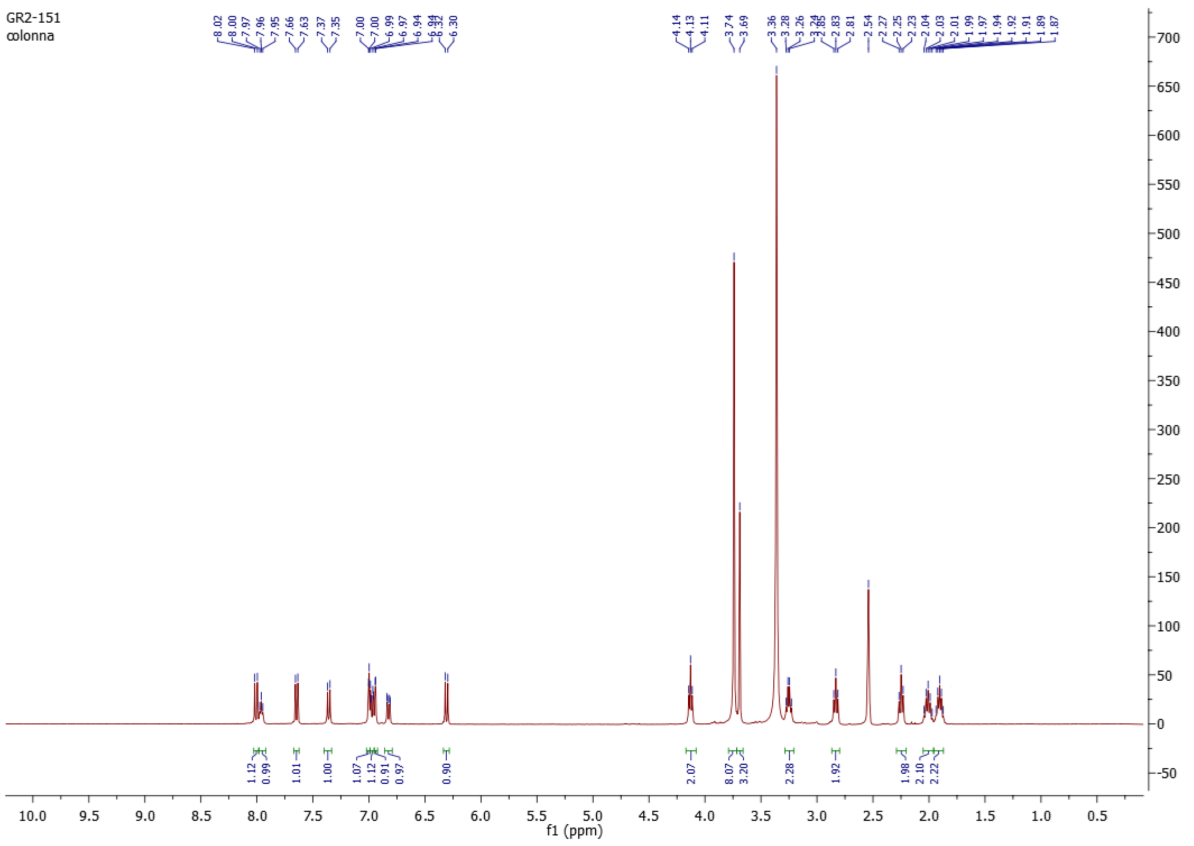

^{1}H NMR spectrum of compound **7b** (400 MHz, DMSO-d_6)

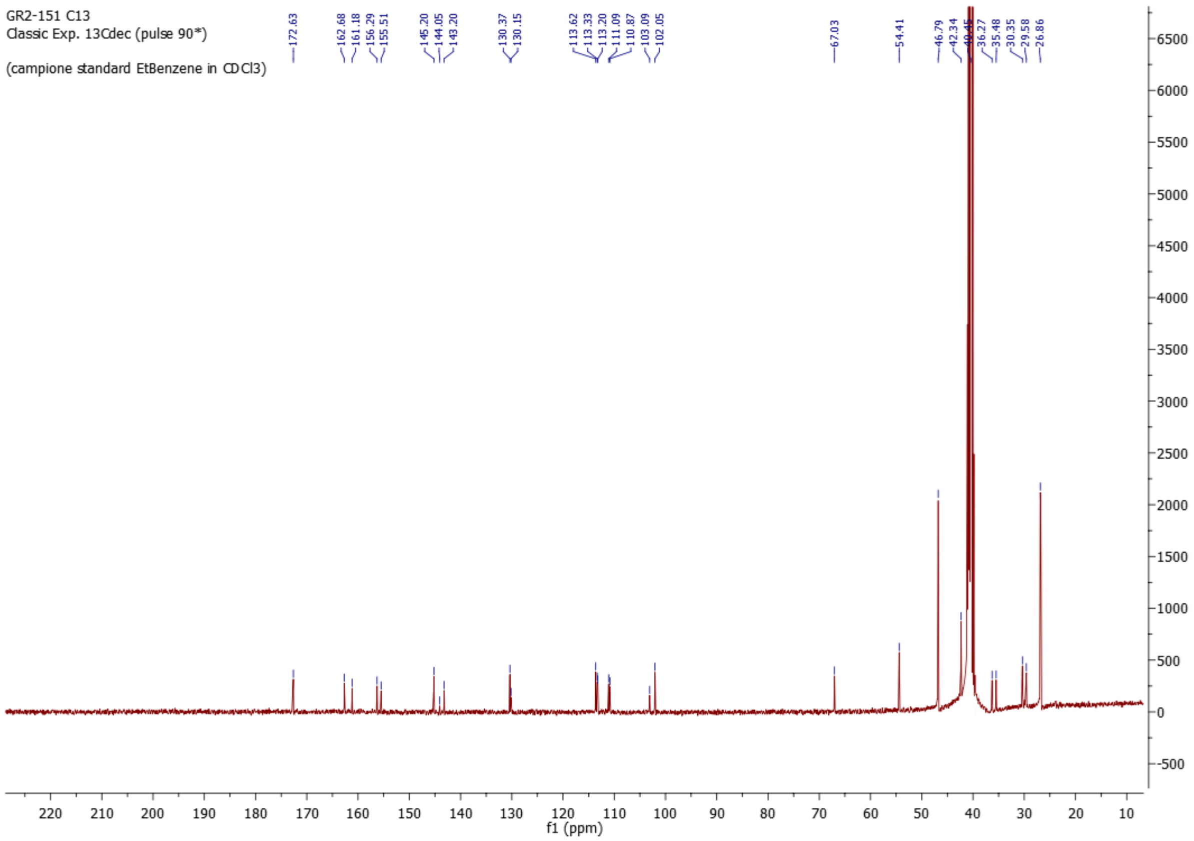

^{13}C NMR spectrum of compound **7b** (100 MHz, DMSO-d_6)

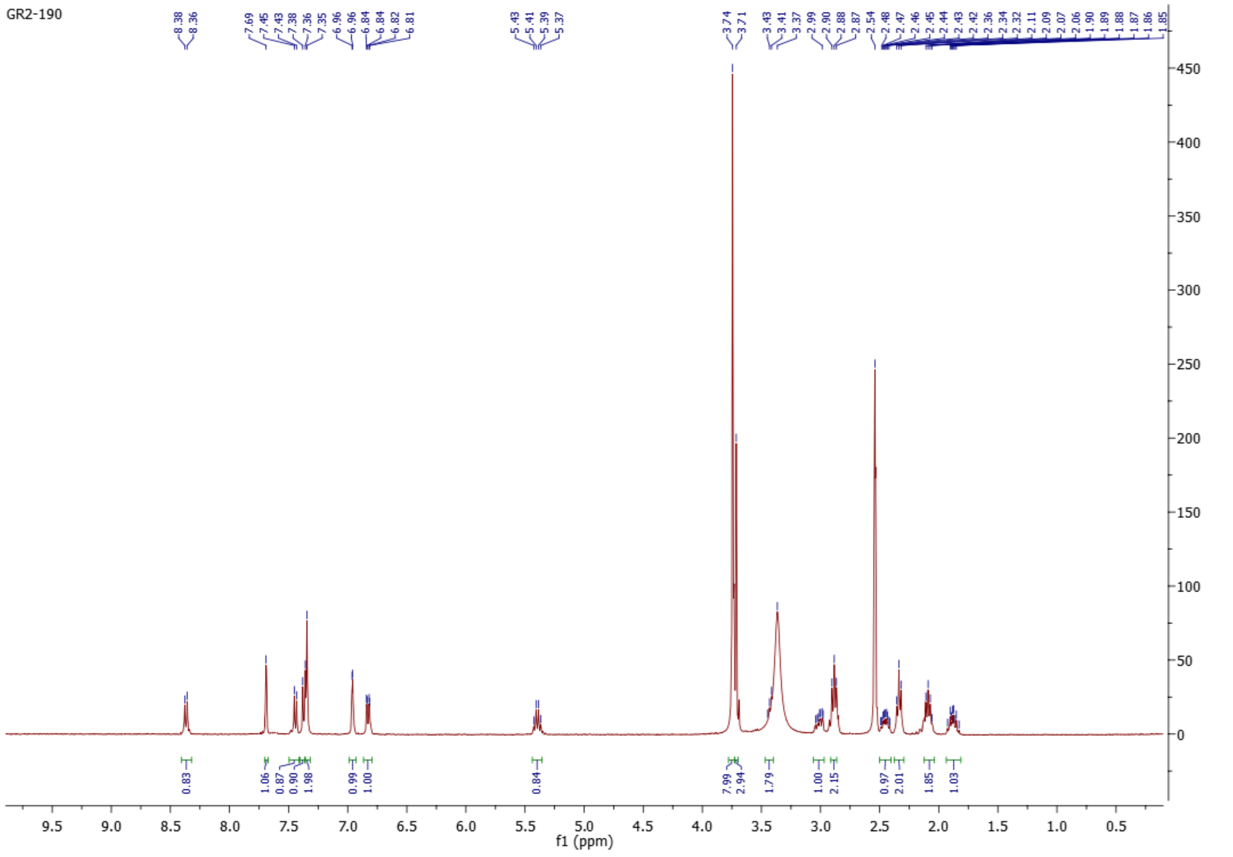

^{1}H NMR spectrum of compound **8** (400 MHz, DMSO-d_6)

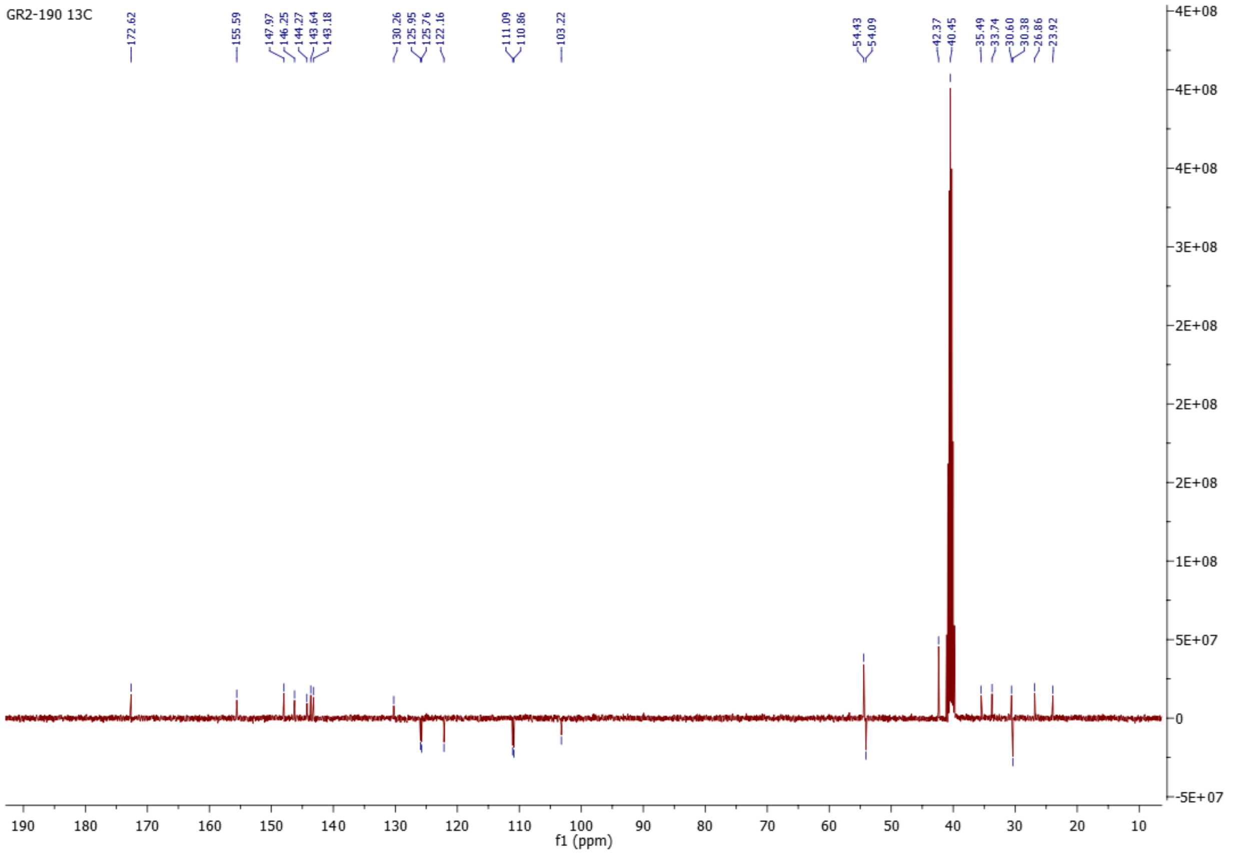

^{13}C NMR spectrum of compound **8** (100 MHz, DMSO-d_6)

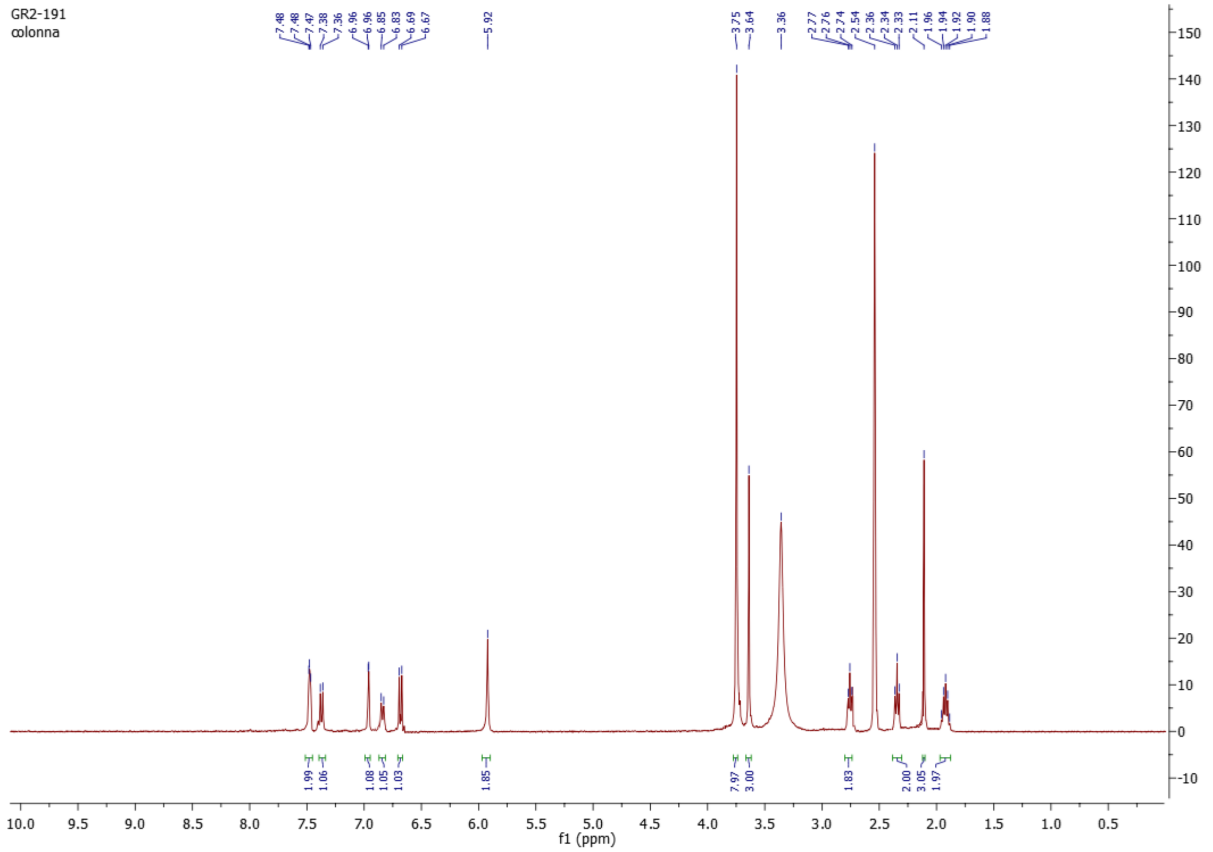

^{1}H NMR spectrum of compound **9** (400 MHz, DMSO-d_6)

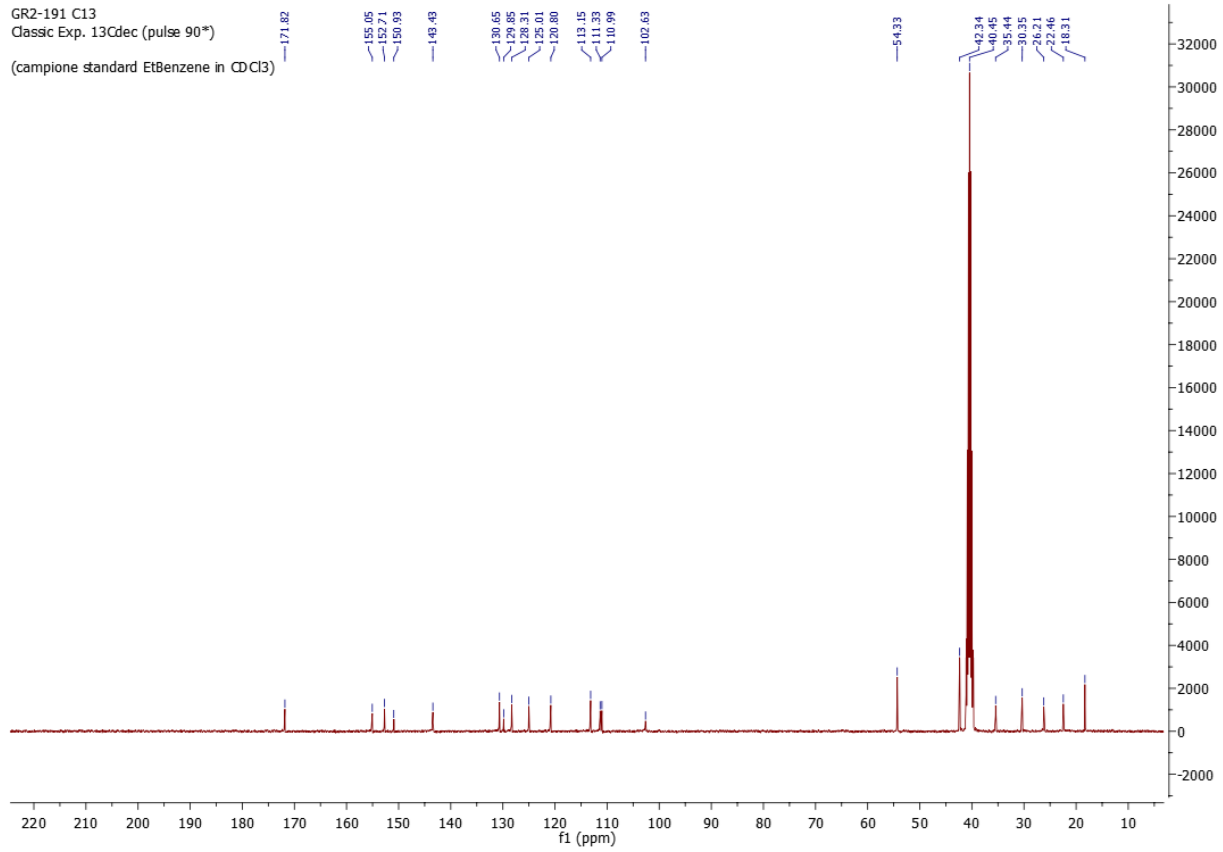

^{13}C NMR spectrum of compound **9** (100 MHz, DMSO-d_6)

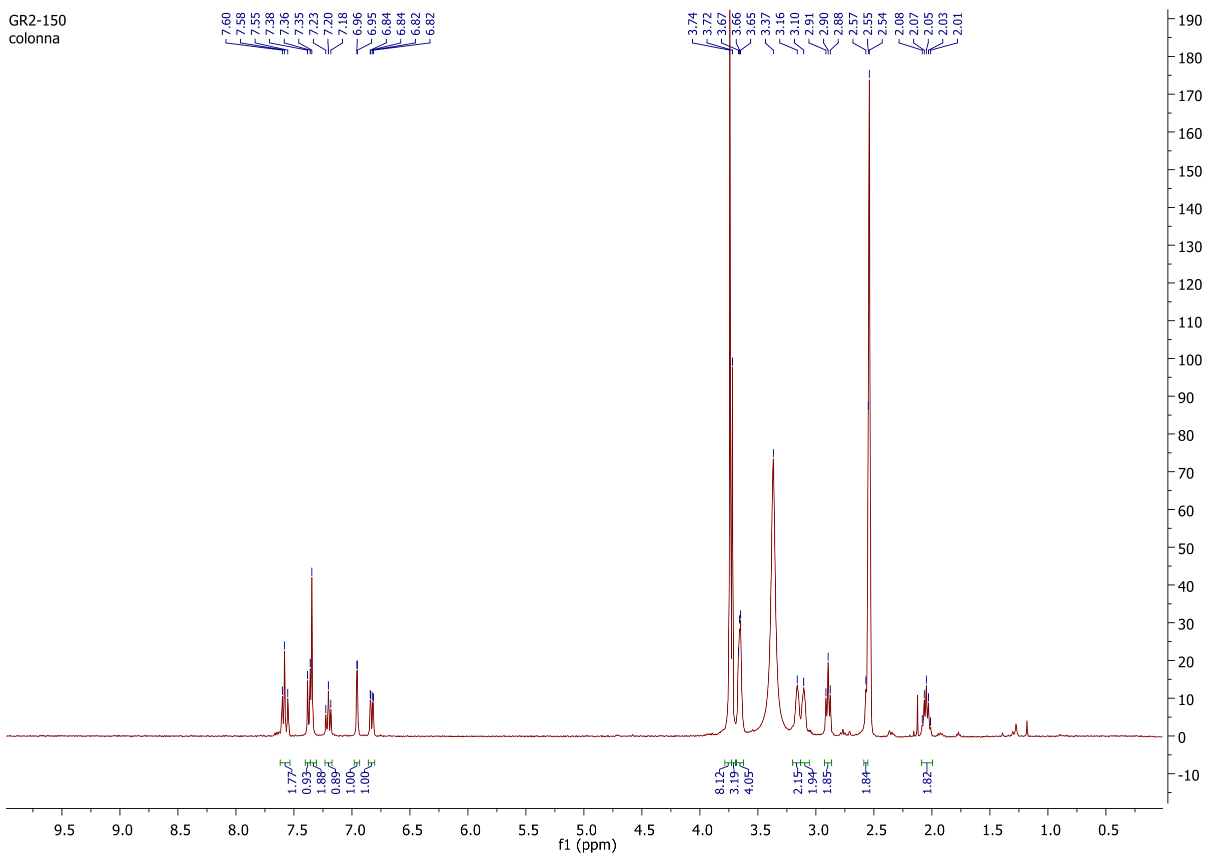

^{1}H NMR spectrum of compound **13a** (400 MHz, DMSO-d_6)

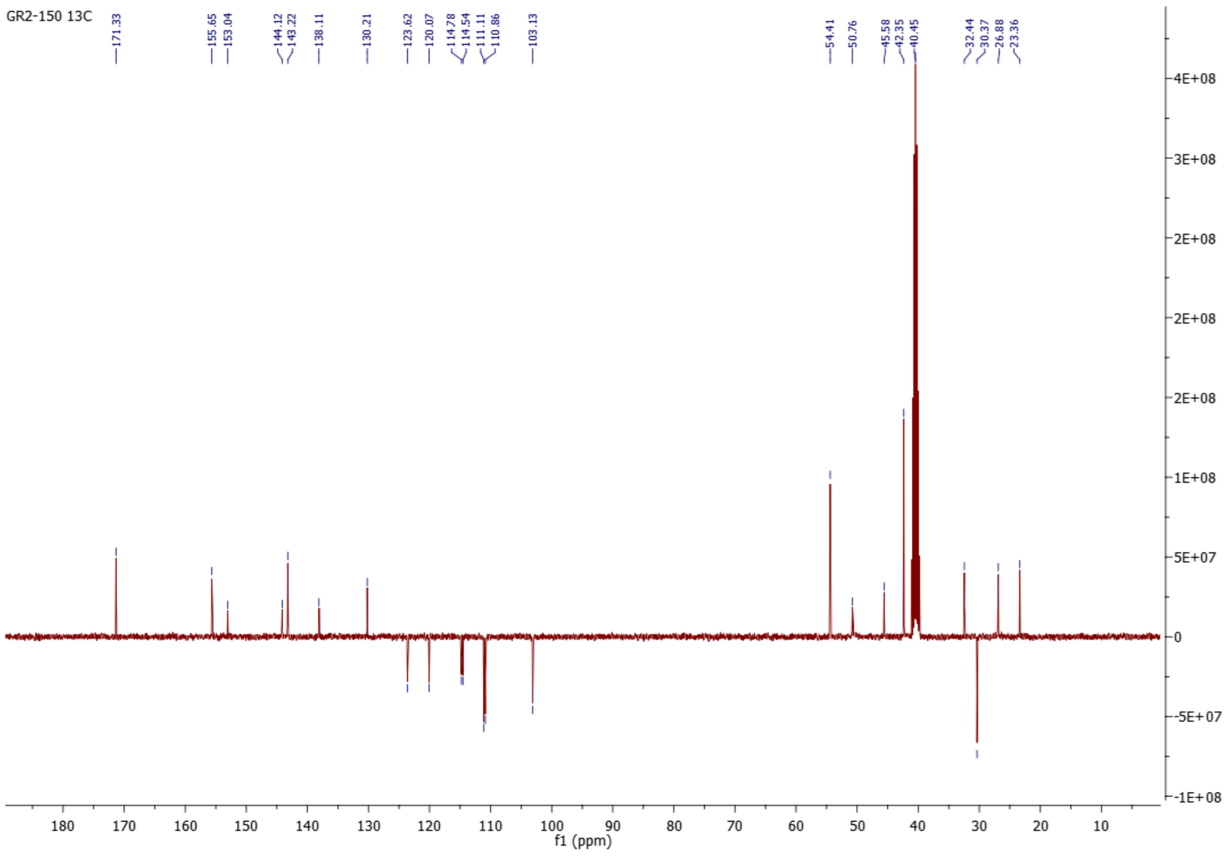

^{13}C NMR spectrum of compound **13a** (100 MHz, DMSO-d_6)

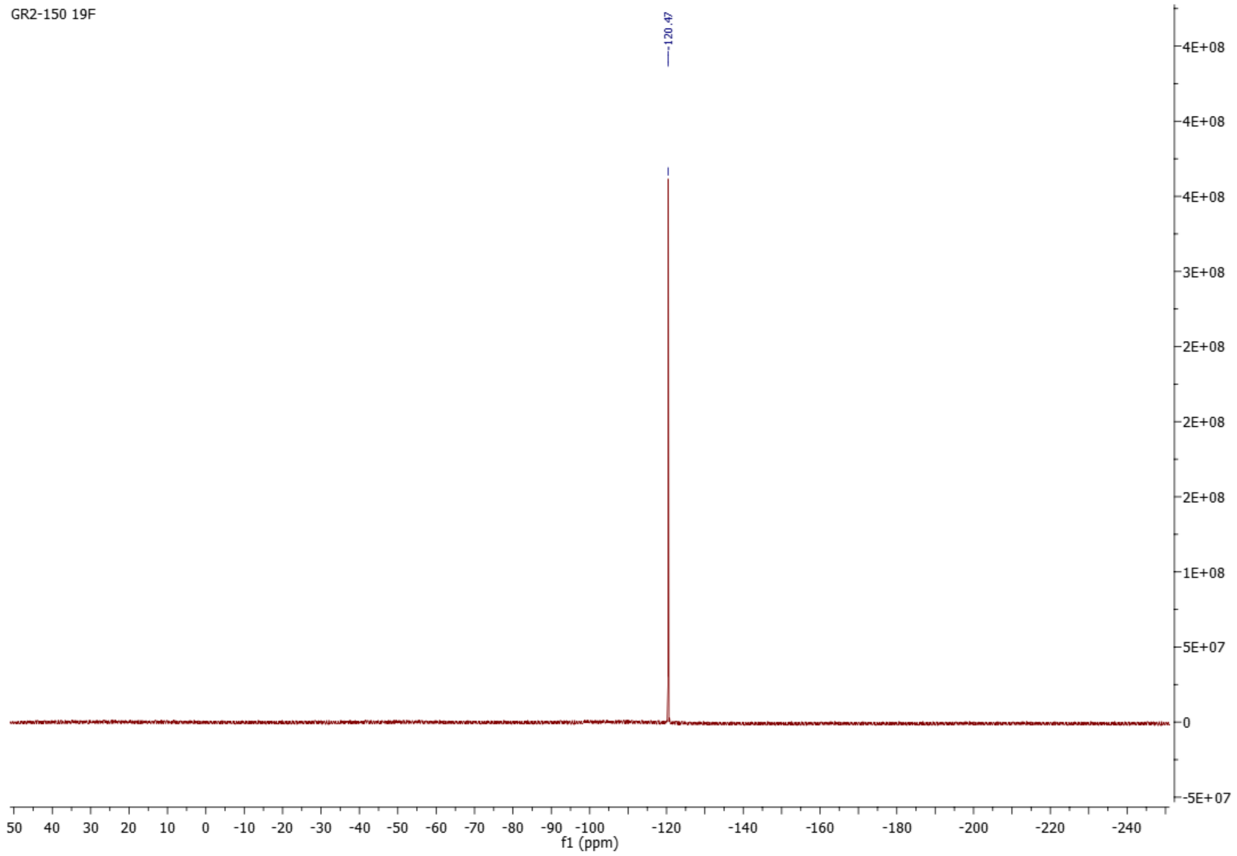

^{19}F NMR spectrum of compound **13a** (376 MHz, DMSO-d_6)

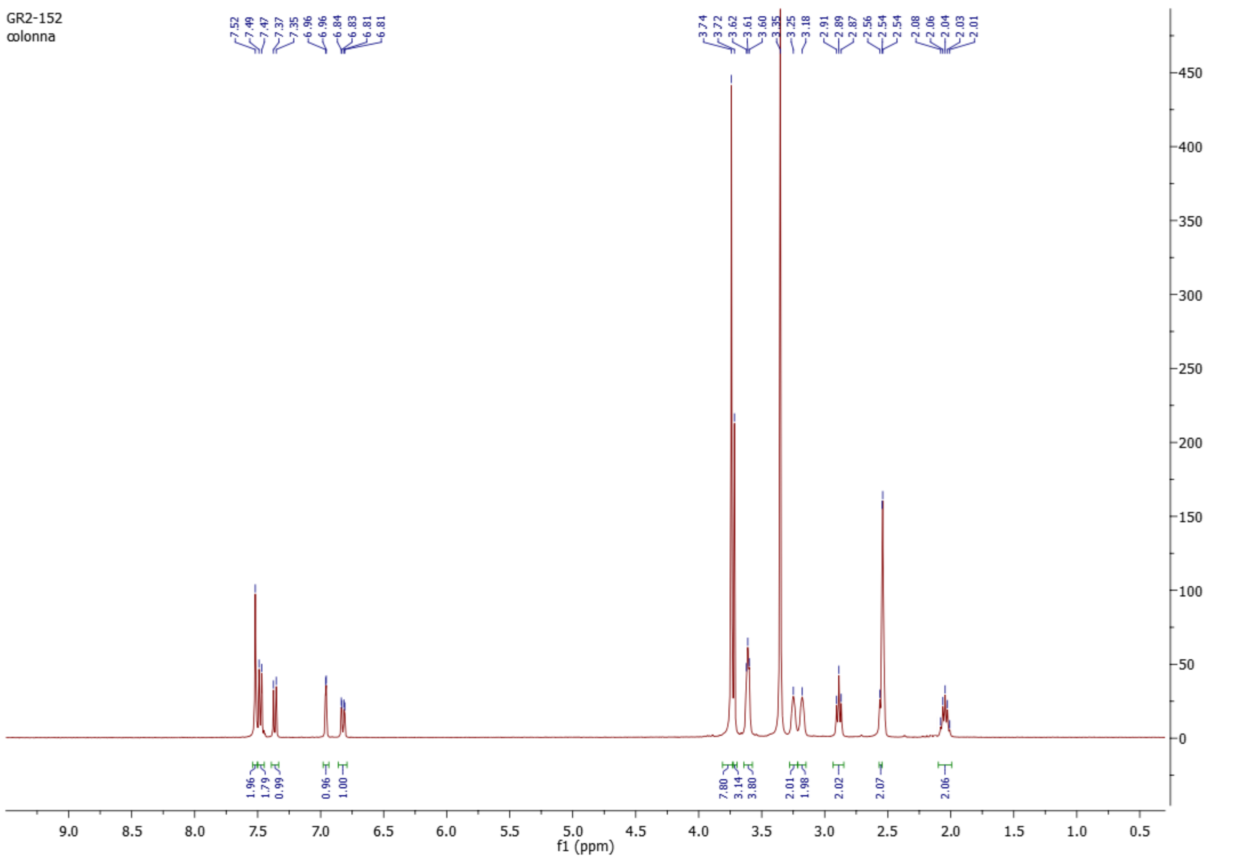

^{1}H NMR spectrum of compound **13b** (400 MHz, DMSO-d_6)

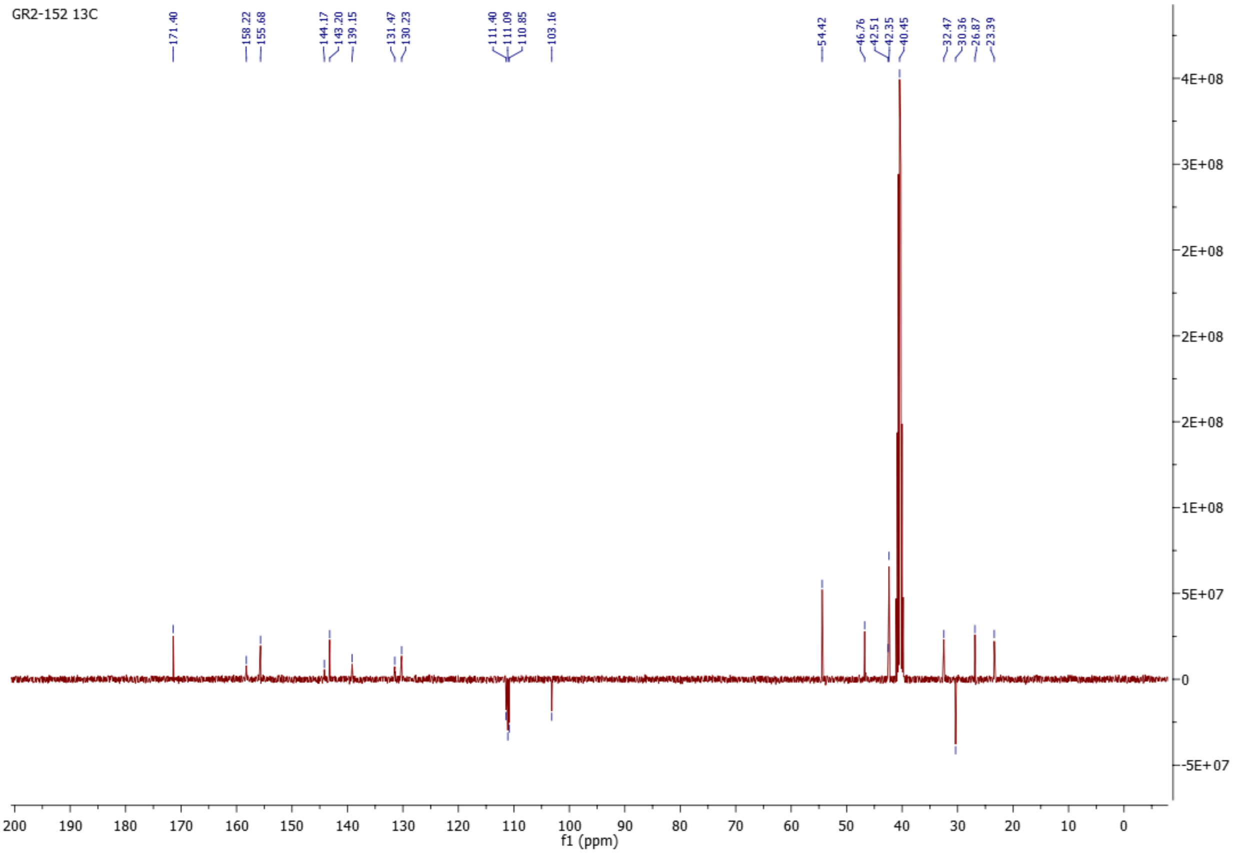

^{13}C NMR spectrum of compound **13b** (100 MHz, DMSO-d_6)

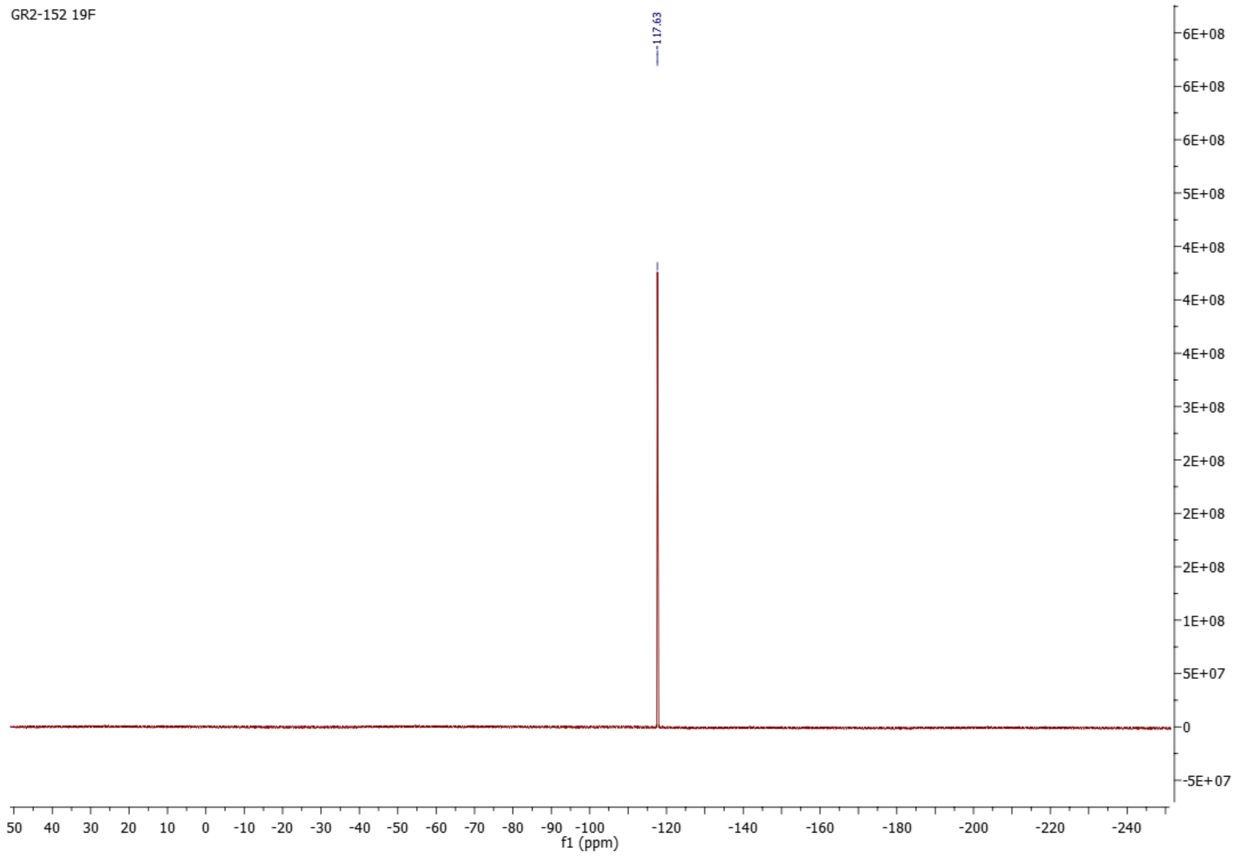

^{19}F NMR spectrum of compound **13b** (376 MHz, DMSO-d_6)

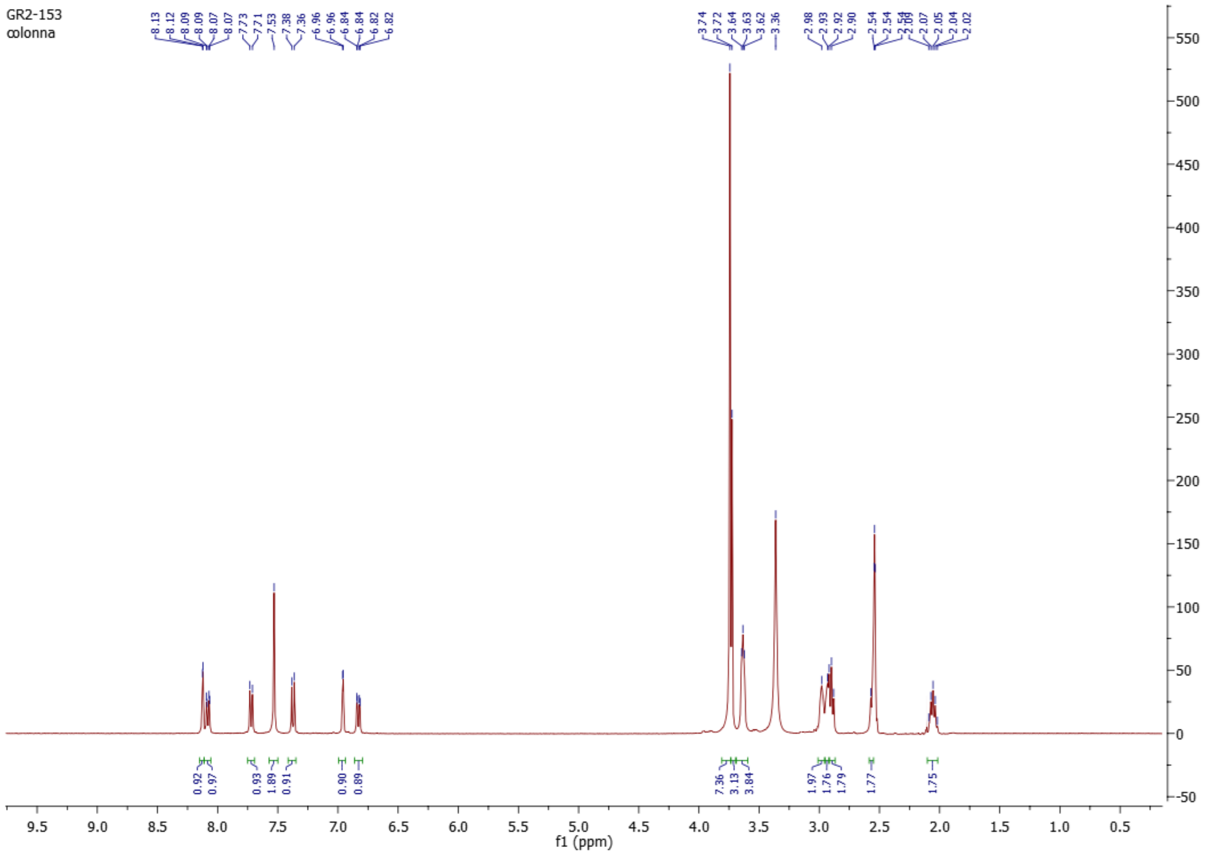

^{1}H NMR spectrum of compound **13c** (400 MHz, DMSO-d_6)

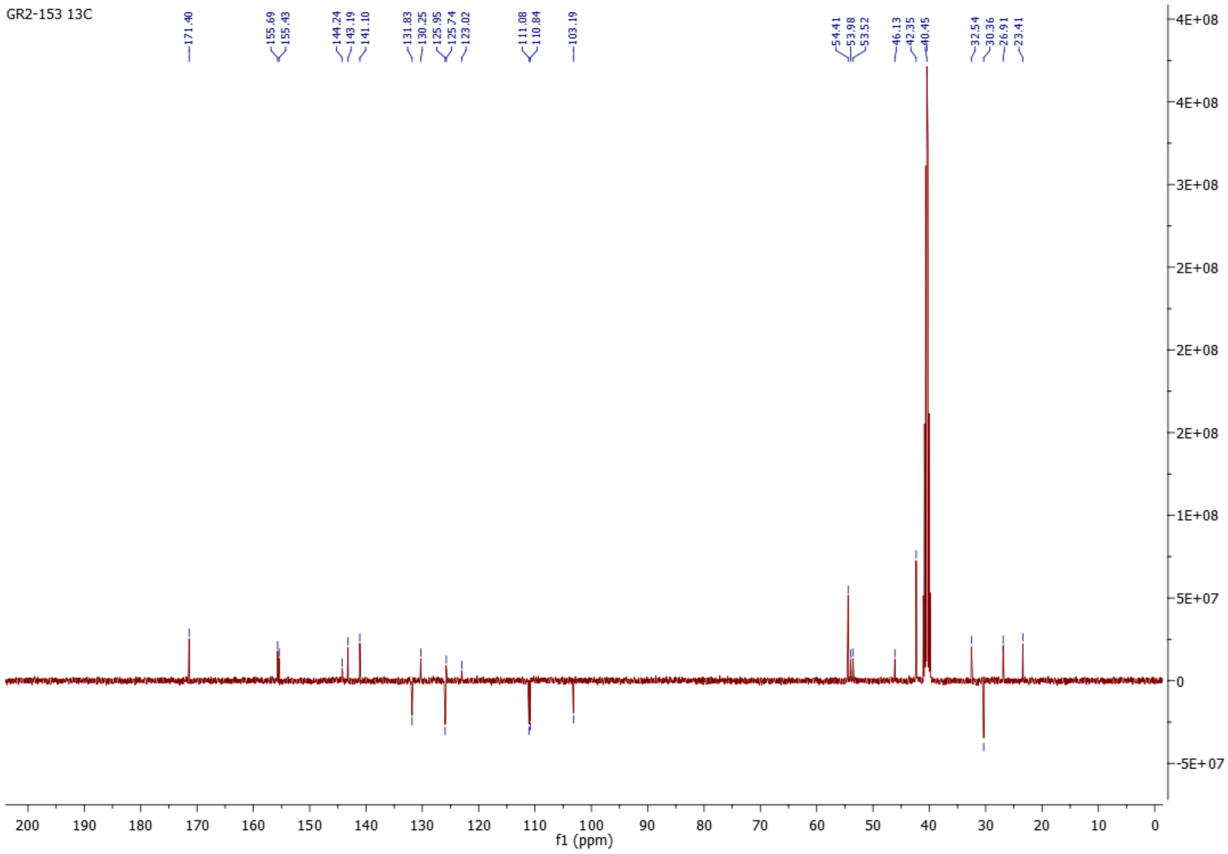

^{13}C NMR spectrum of compound **13c** (100 MHz, DMSO-d_6)

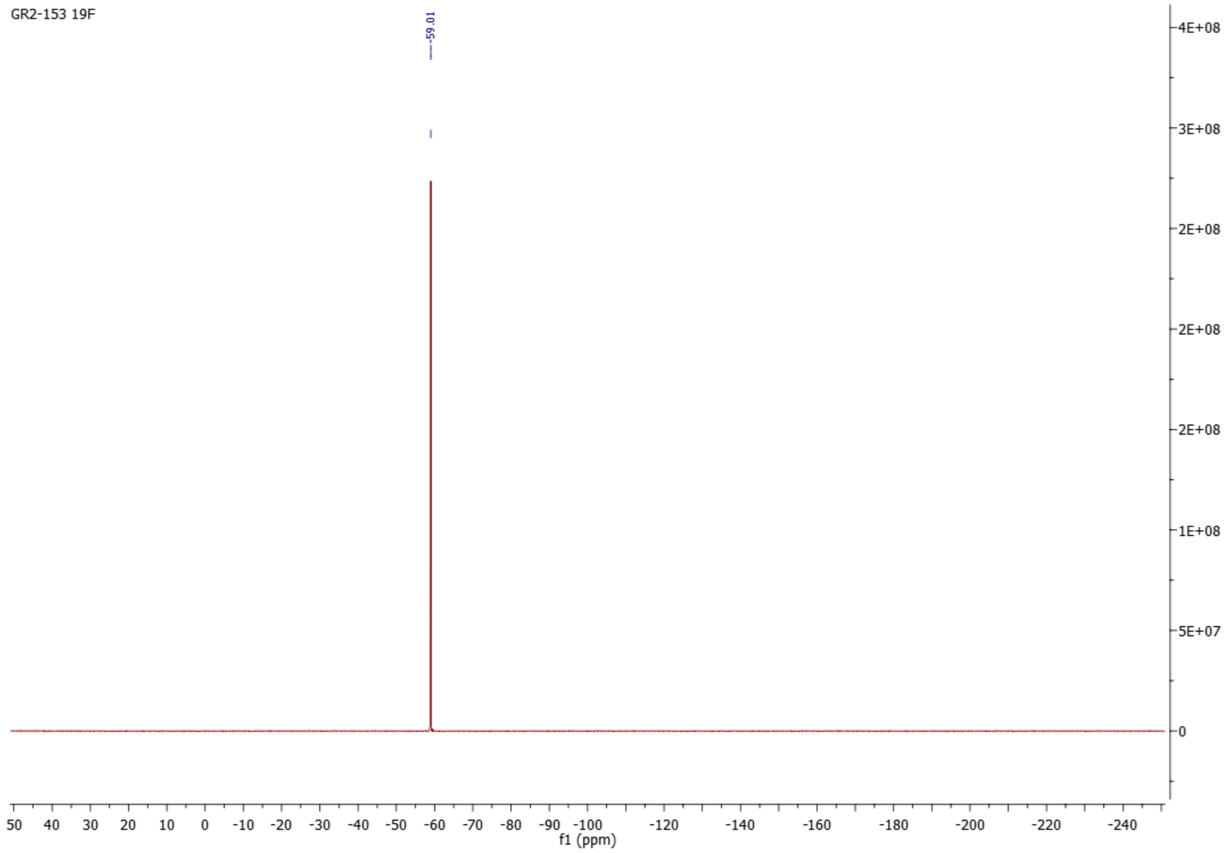

^{19}F NMR spectrum of compound **13c** (376 MHz, DMSO-d_6)

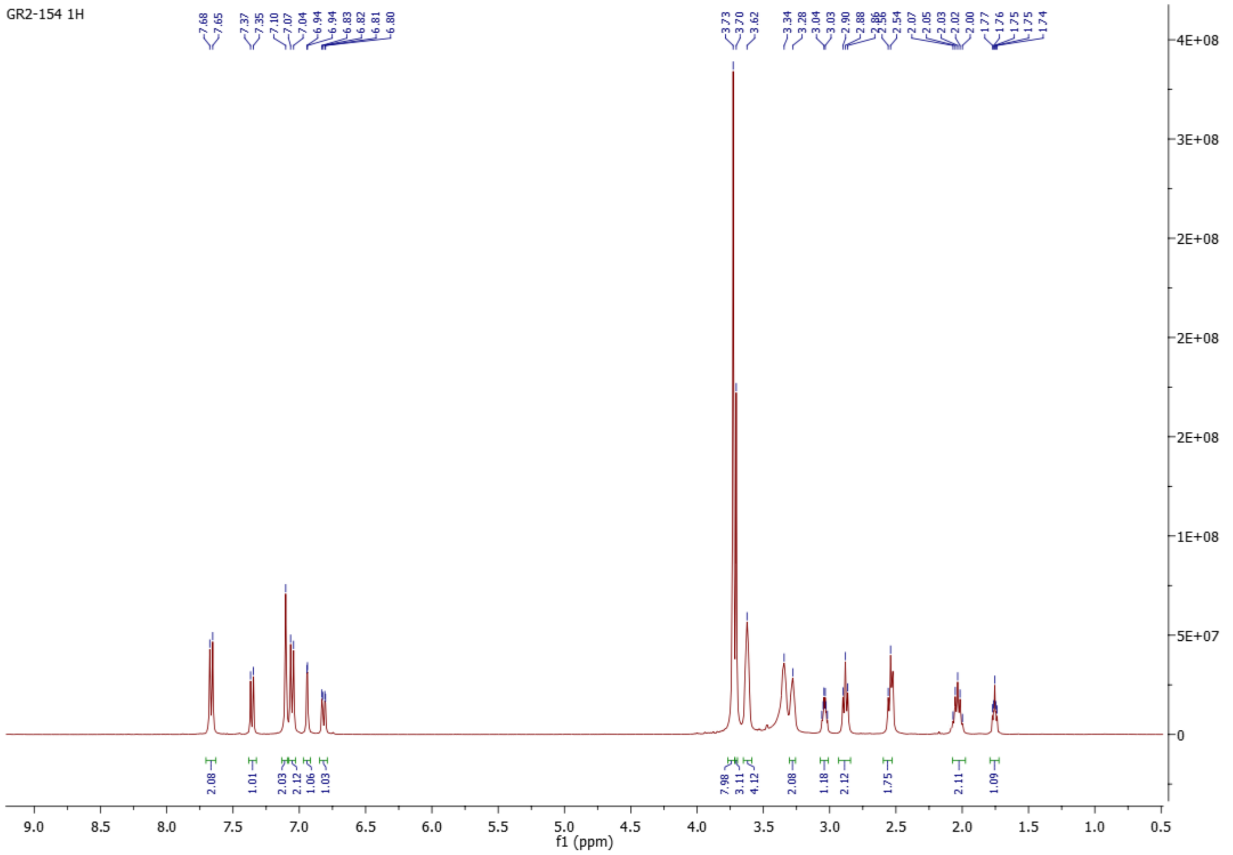

^{1}H NMR spectrum of compound **13d** (400 MHz, DMSO-d_6)

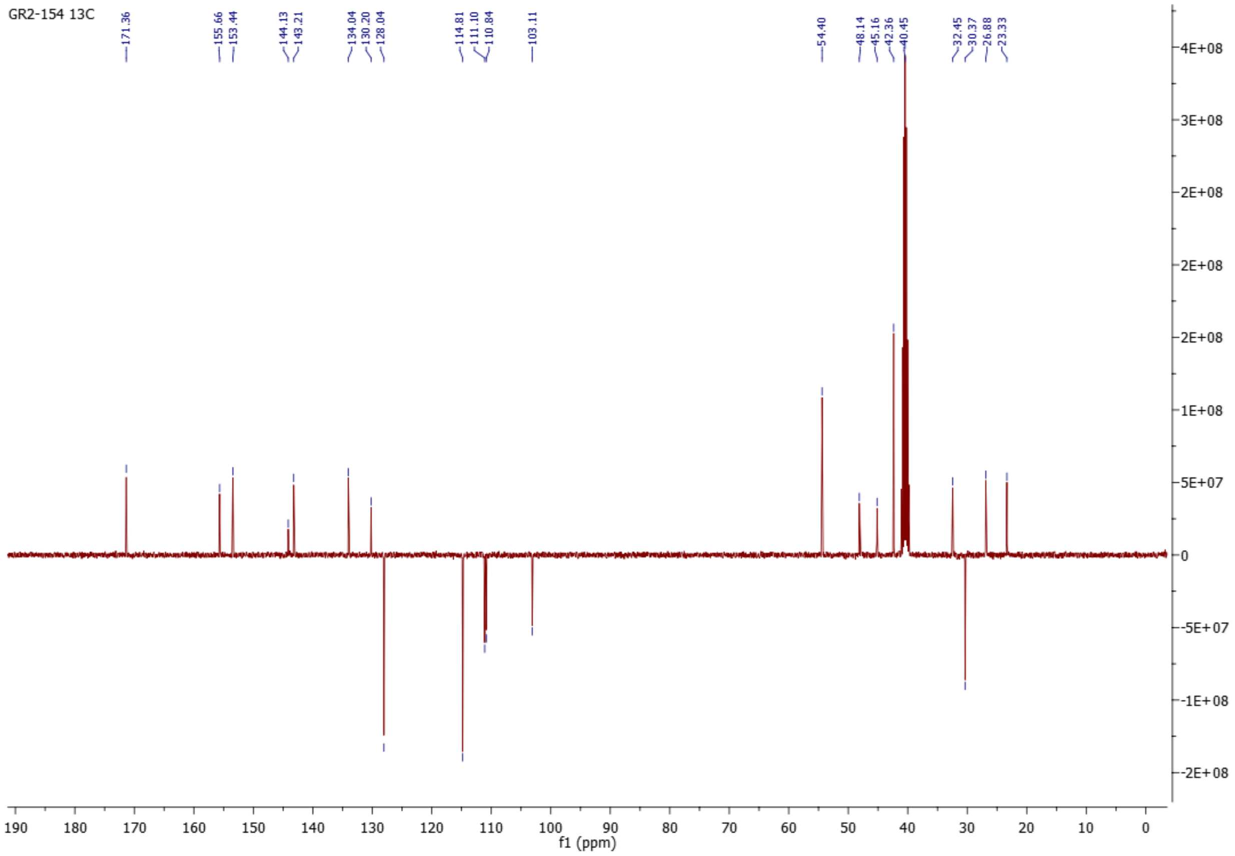

^{13}C NMR spectrum of compound **13d** (100 MHz, DMSO-d_6)

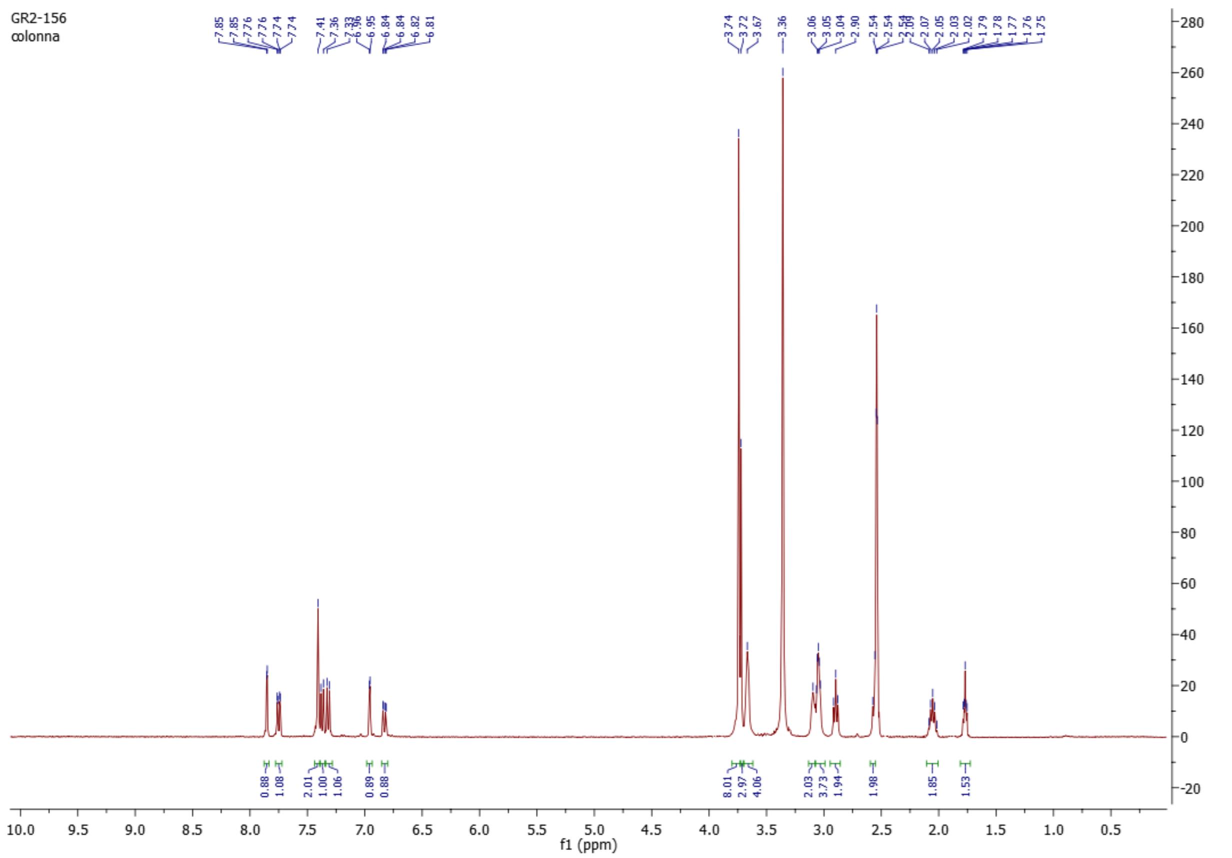

^{1}H NMR spectrum of compound **13e** (400 MHz, DMSO-d_6)

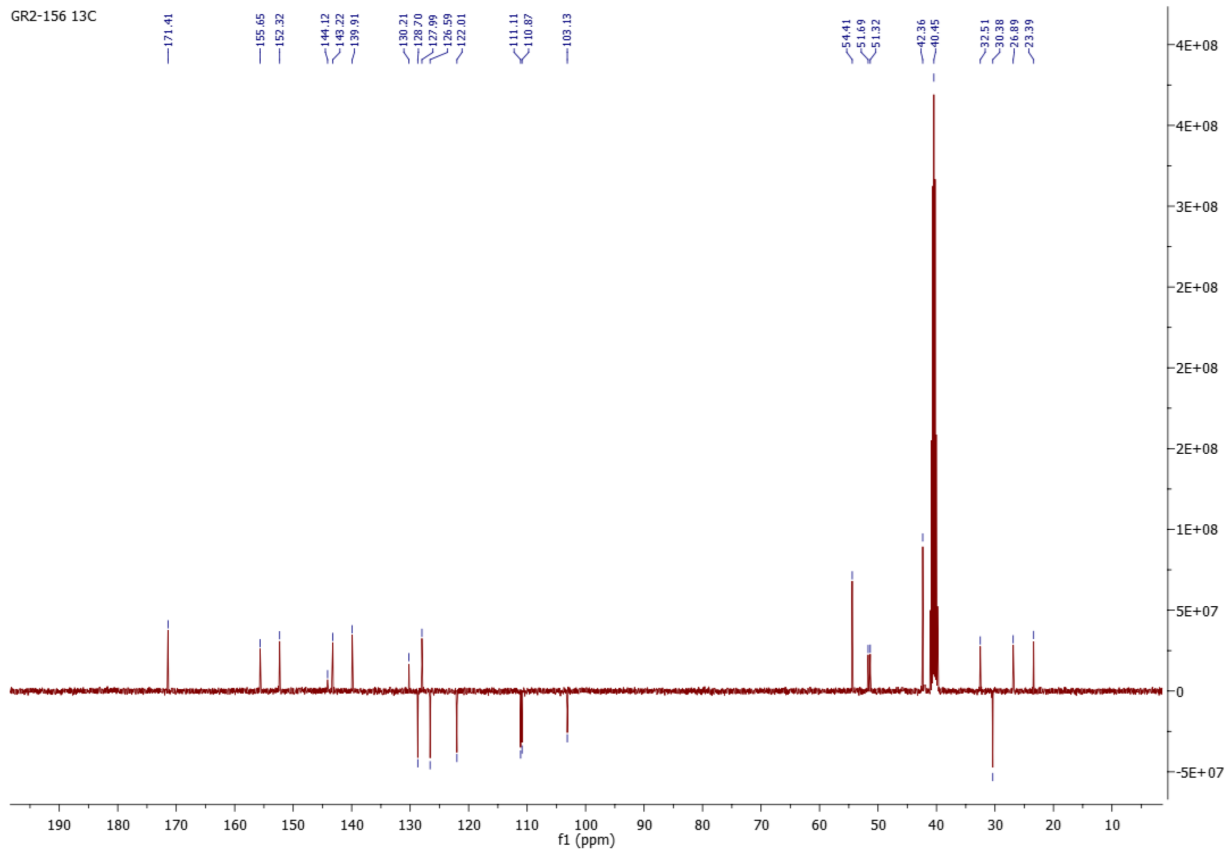

^{13}C NMR spectrum of compound **13e** (100 MHz, DMSO-d_6)

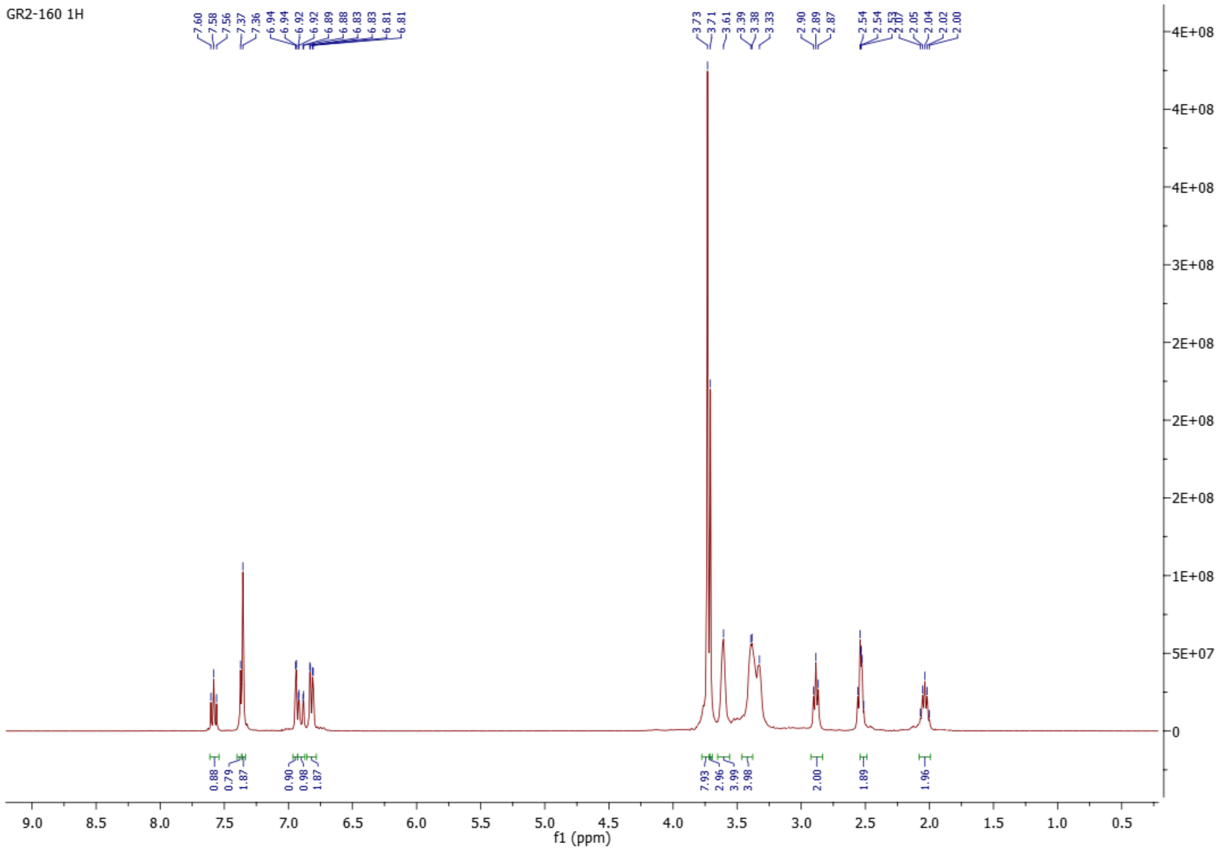

^{1}H NMR spectrum of compound **13f** (400 MHz, DMSO-d_6)

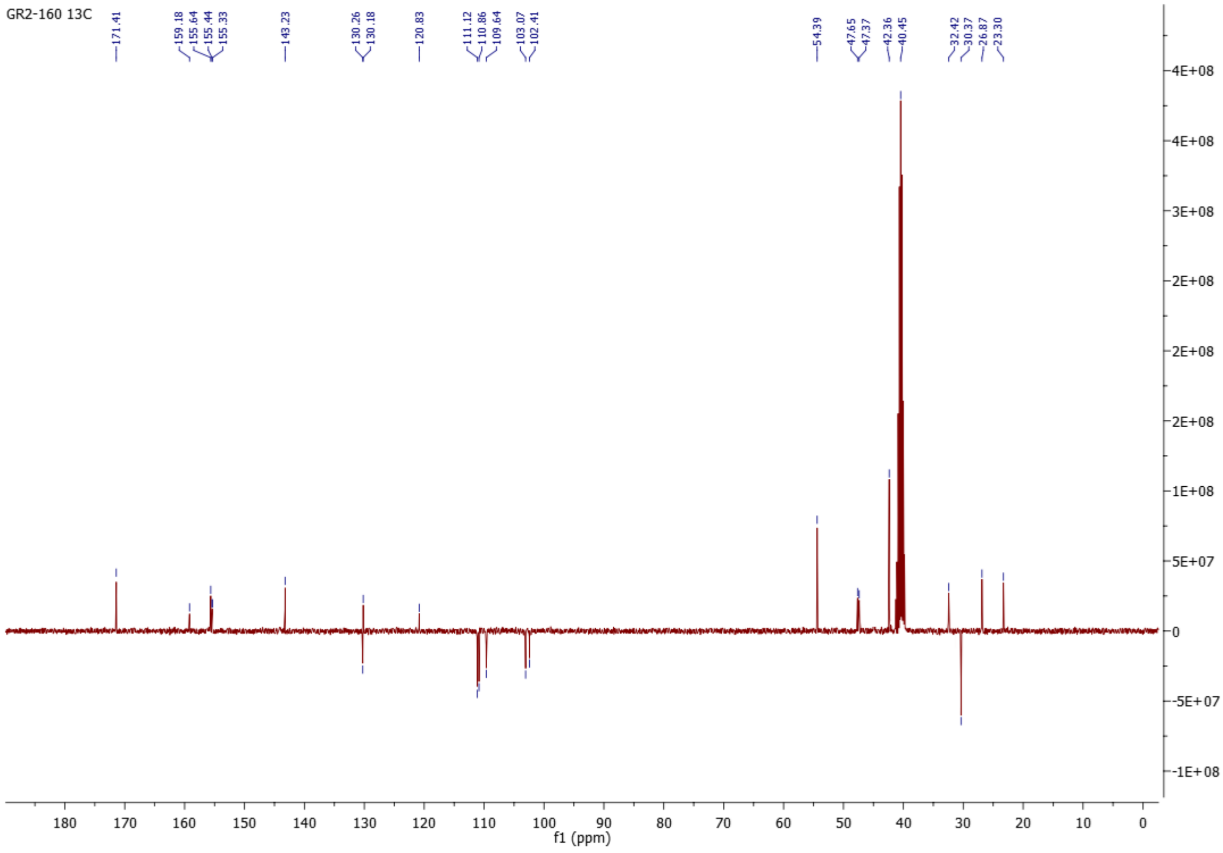

^{13}C NMR spectrum of compound **13f** (100 MHz, DMSO-d_6)

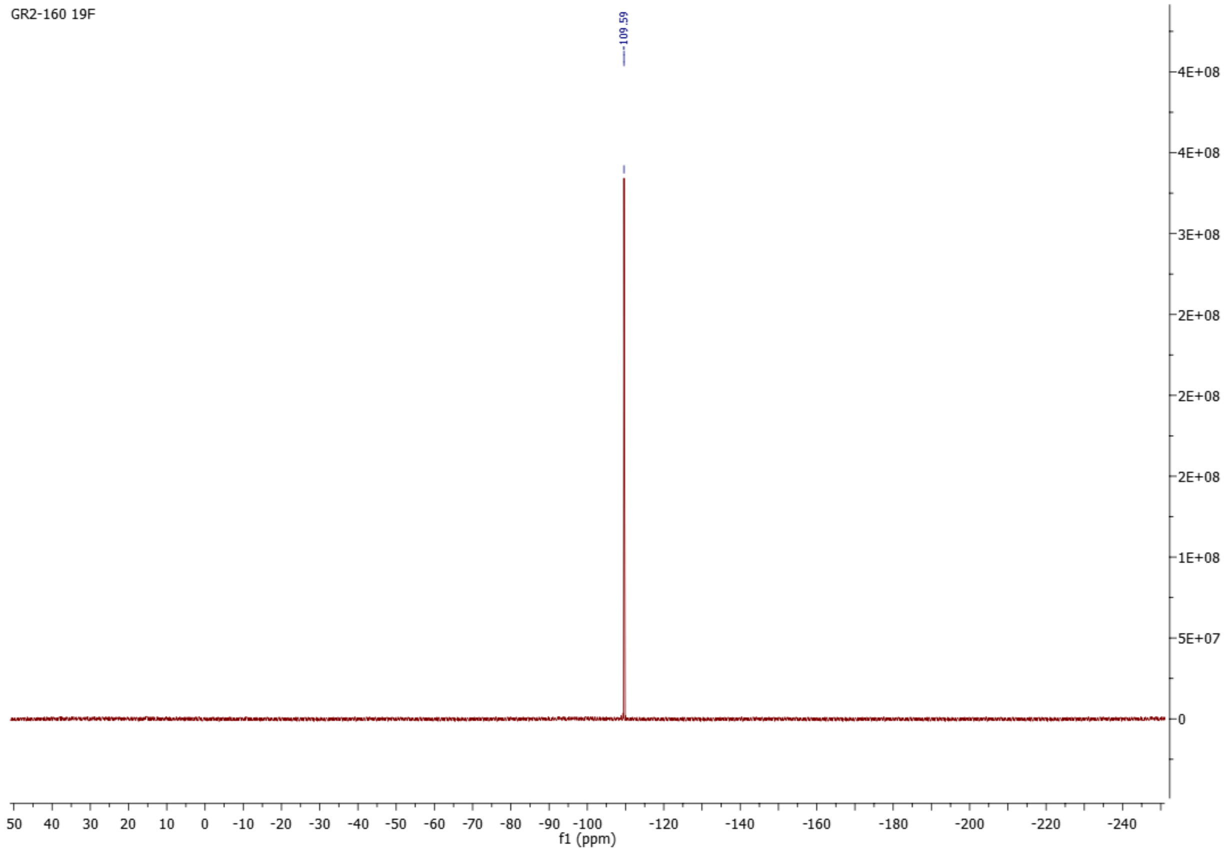

^{19}F NMR spectrum of compound **13f** (376 MHz, DMSO-d_6)

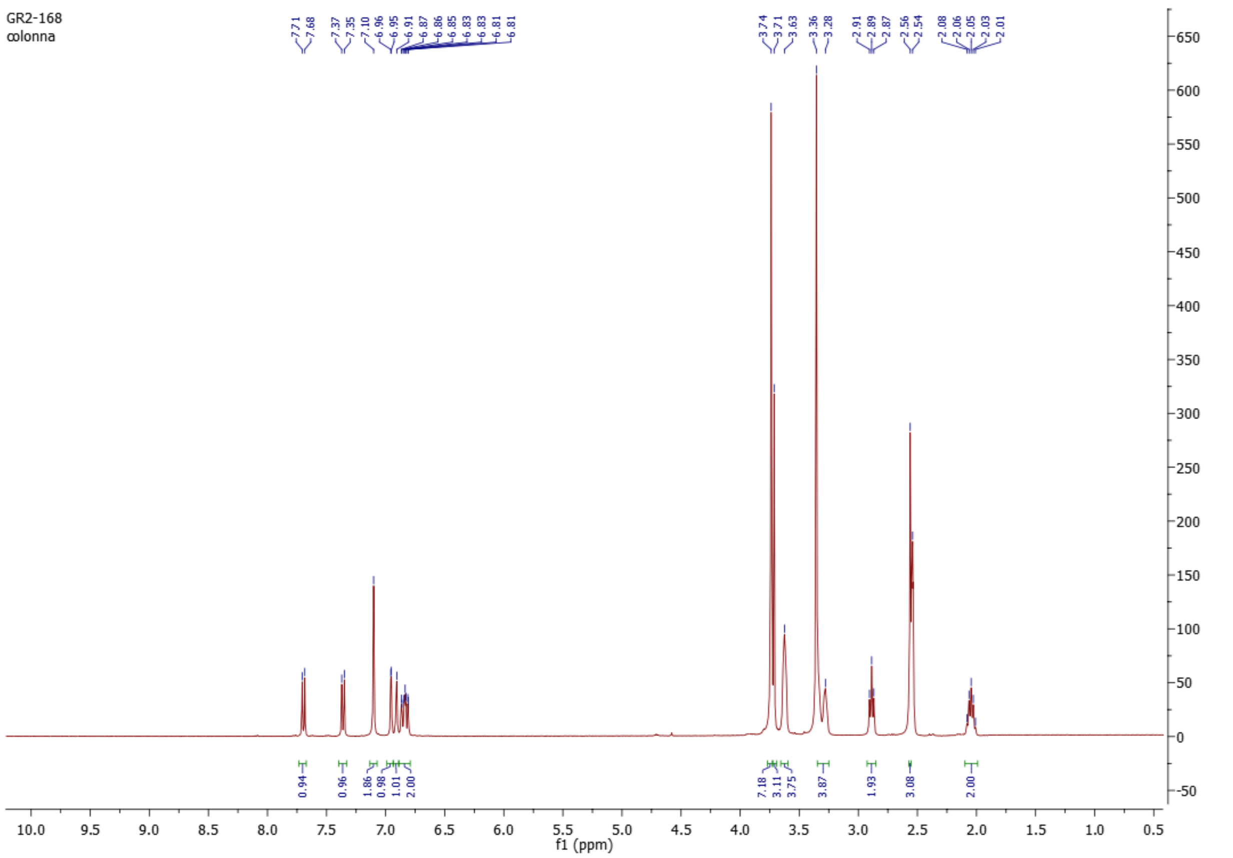

^{1}H NMR spectrum of compound **13g** (400 MHz, DMSO-d_6)

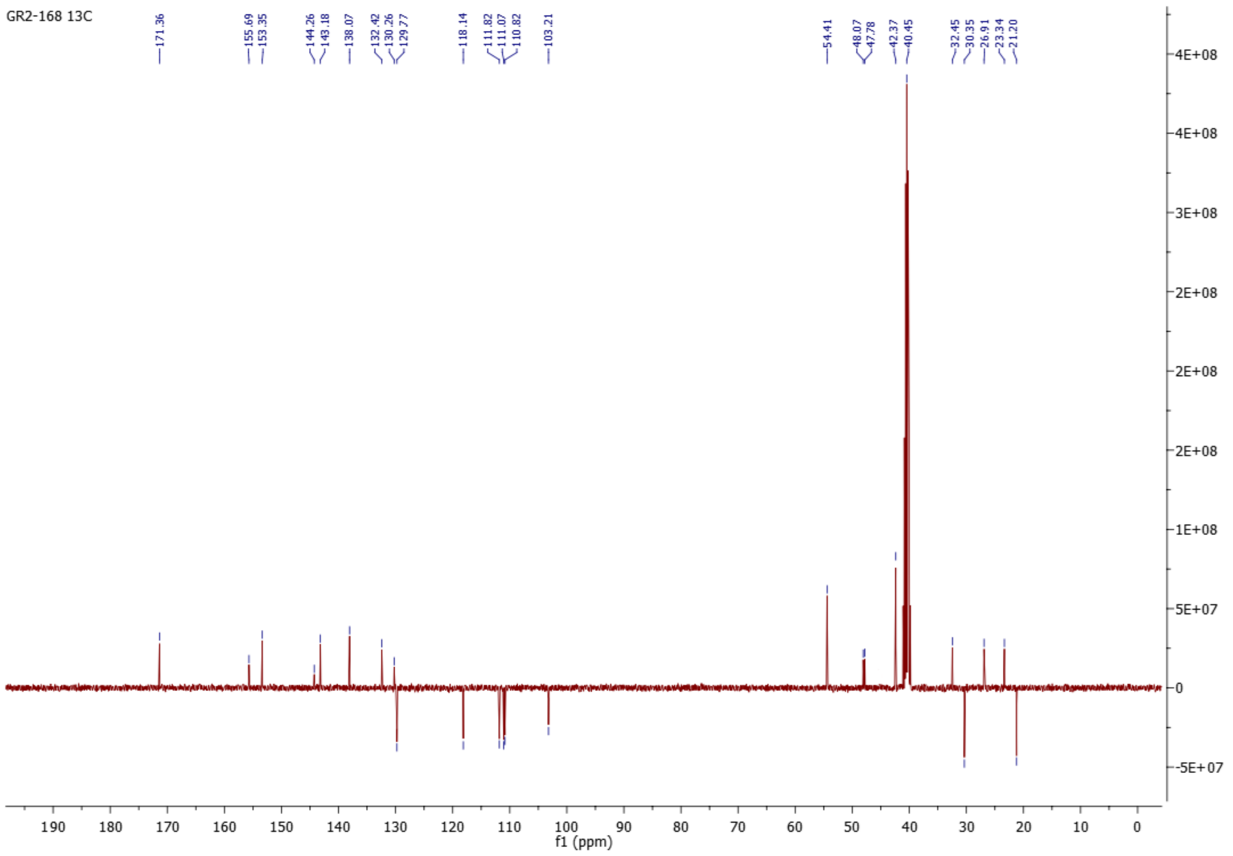

^{13}C NMR spectrum of compound **13g** (100 MHz, DMSO-d_6)

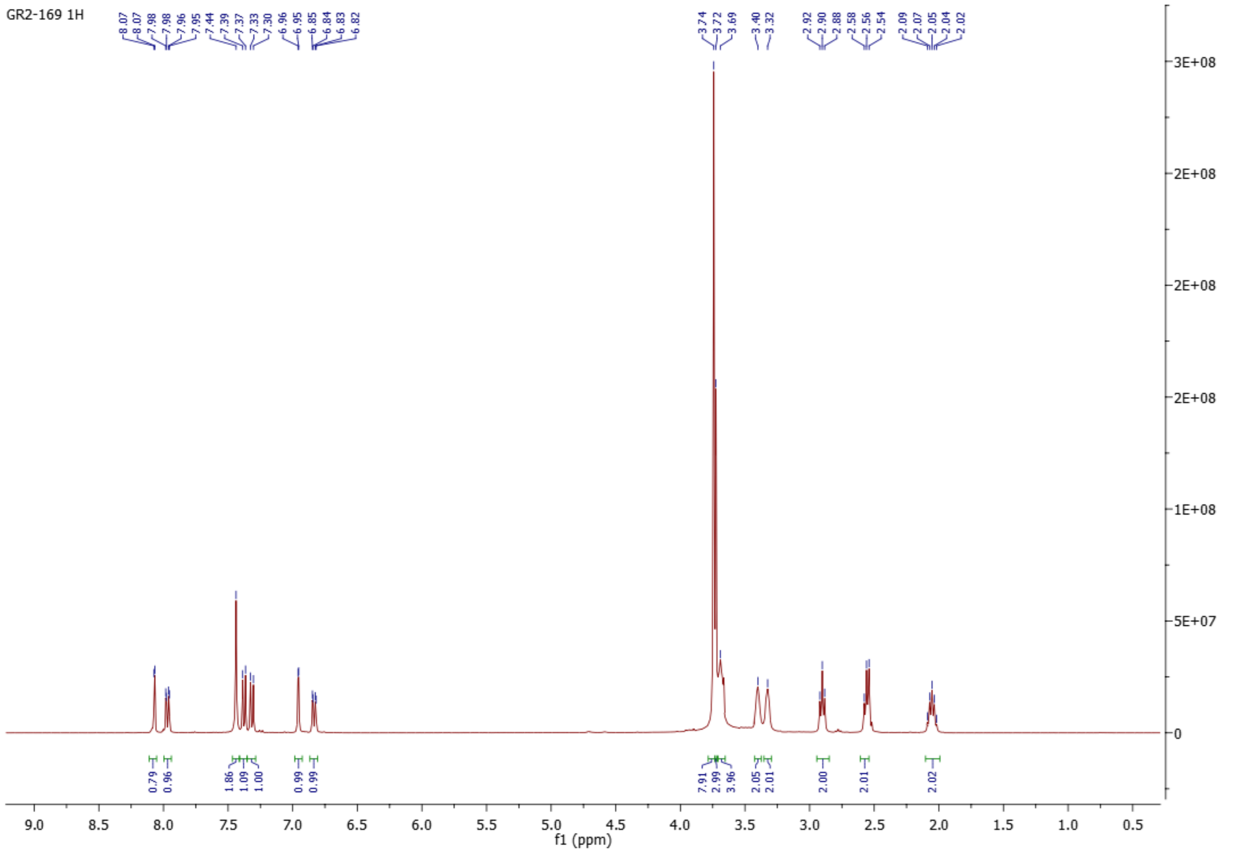

^{1}H NMR spectrum of compound **13h** (400 MHz, DMSO-d_6)

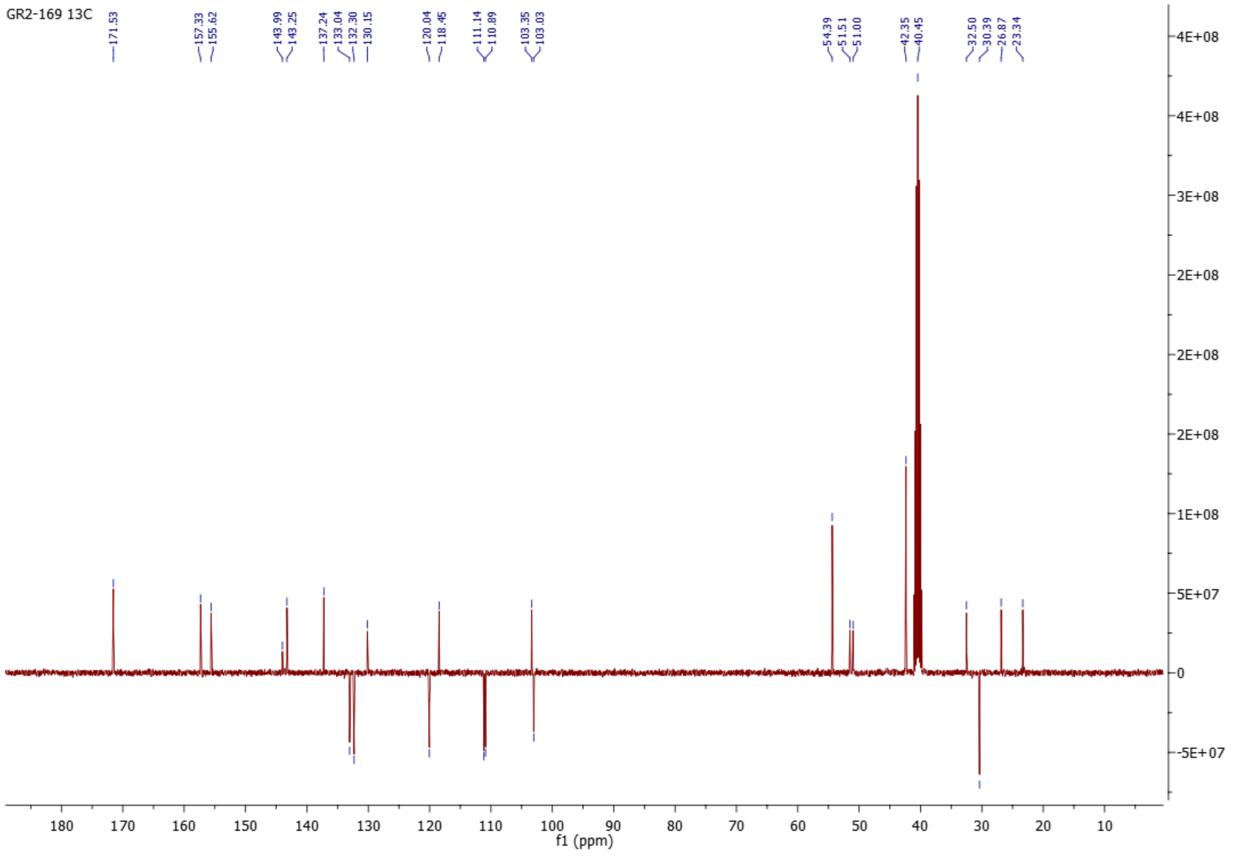

^{13}C NMR spectrum of compound **13h** (100 MHz, DMSO-d_6)

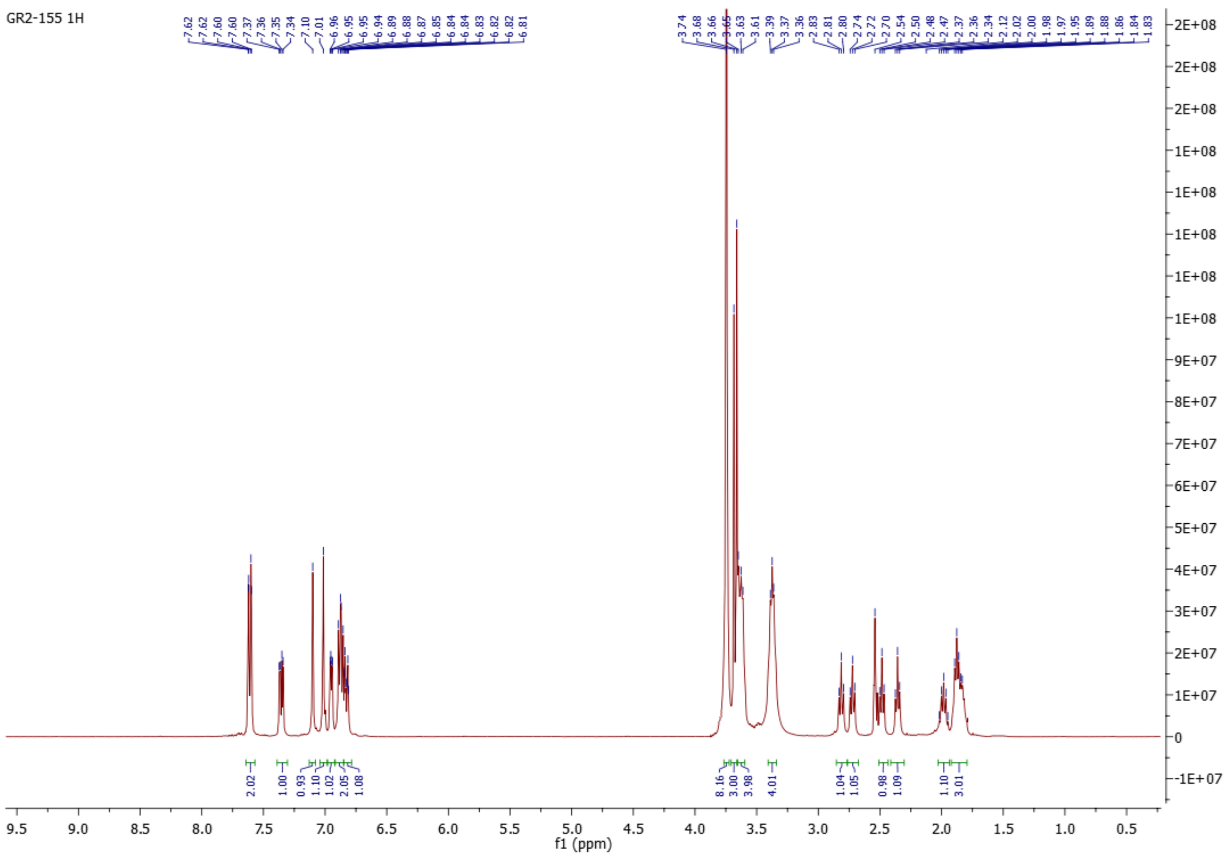

^{1}H NMR spectrum of compound **14a** (400 MHz, DMSO-d_6)

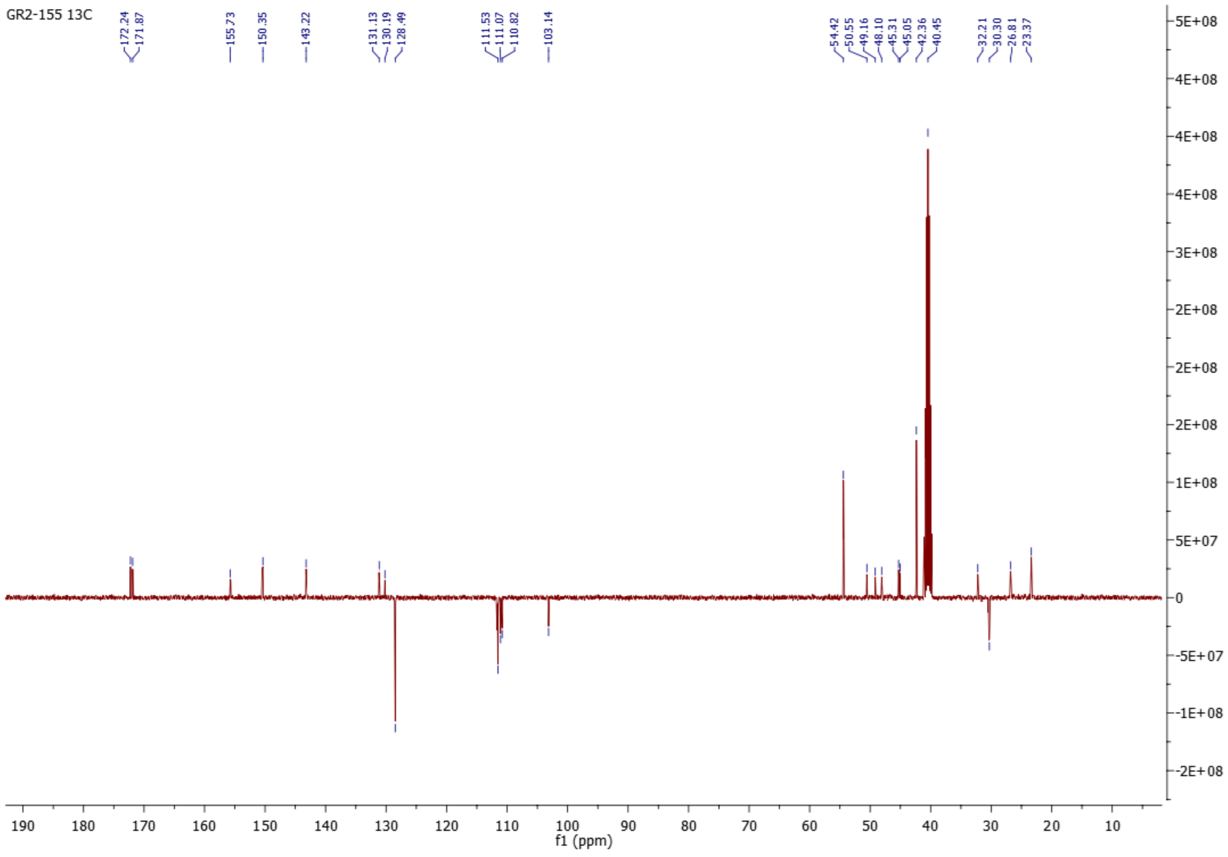

^{13}C NMR spectrum of compound **14a** (100 MHz, DMSO-d_6)

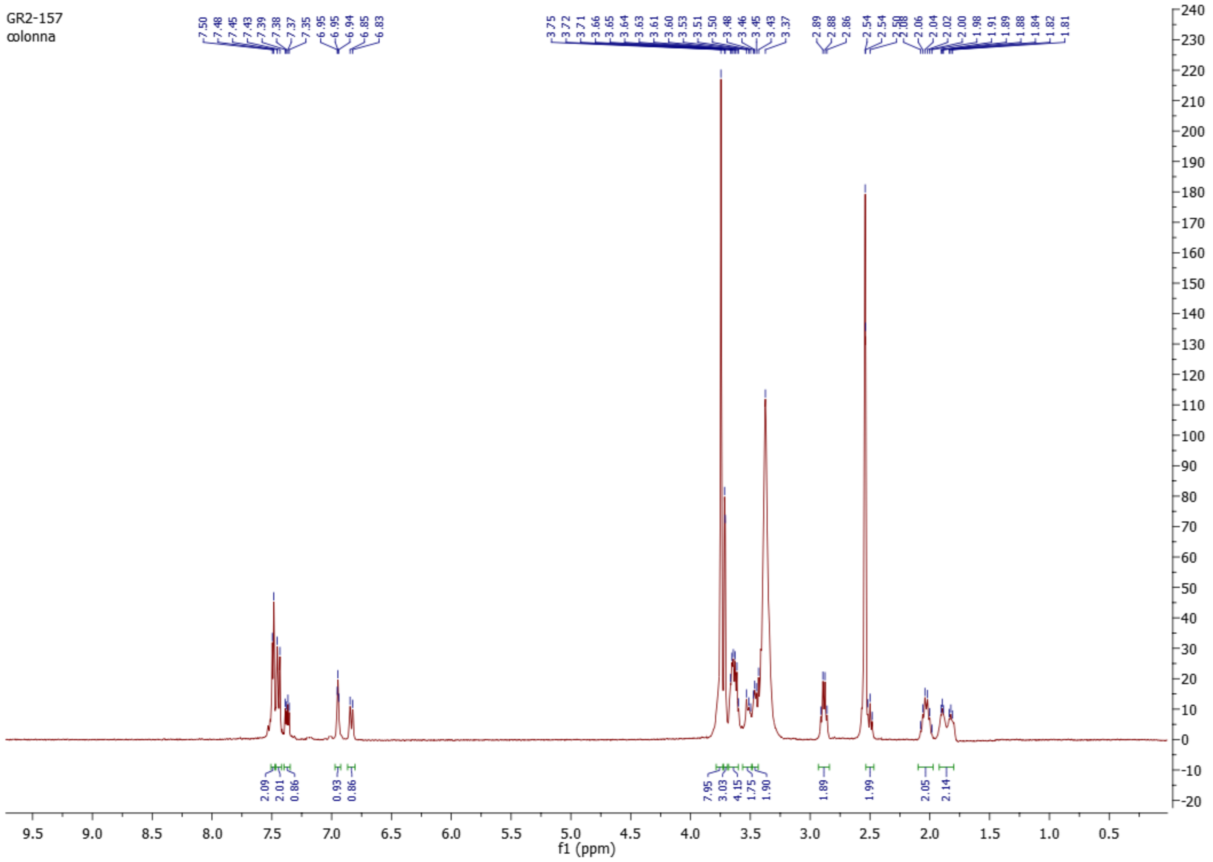

^{1}H NMR spectrum of compound **14b** (400 MHz, DMSO-d_6)

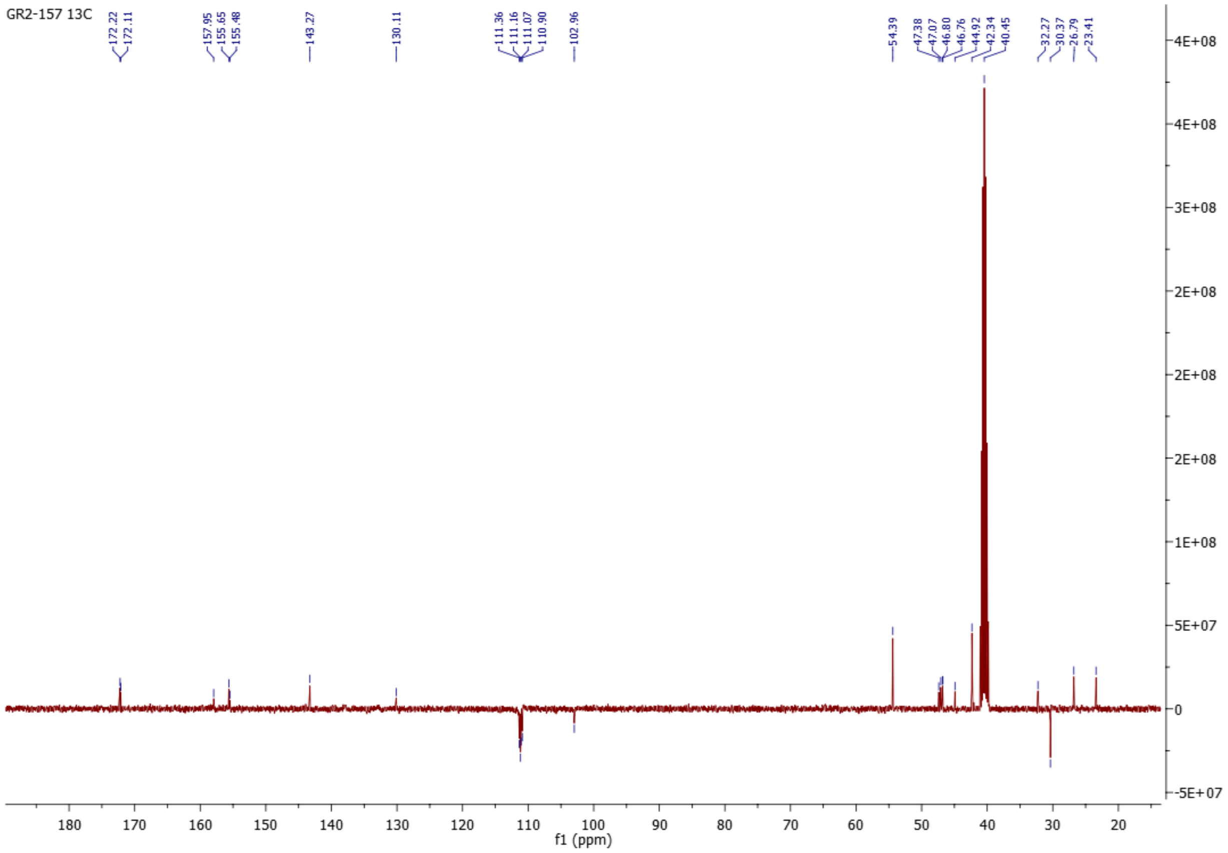

^{13}C NMR spectrum of compound **14b** (100 MHz, DMSO-d_6)

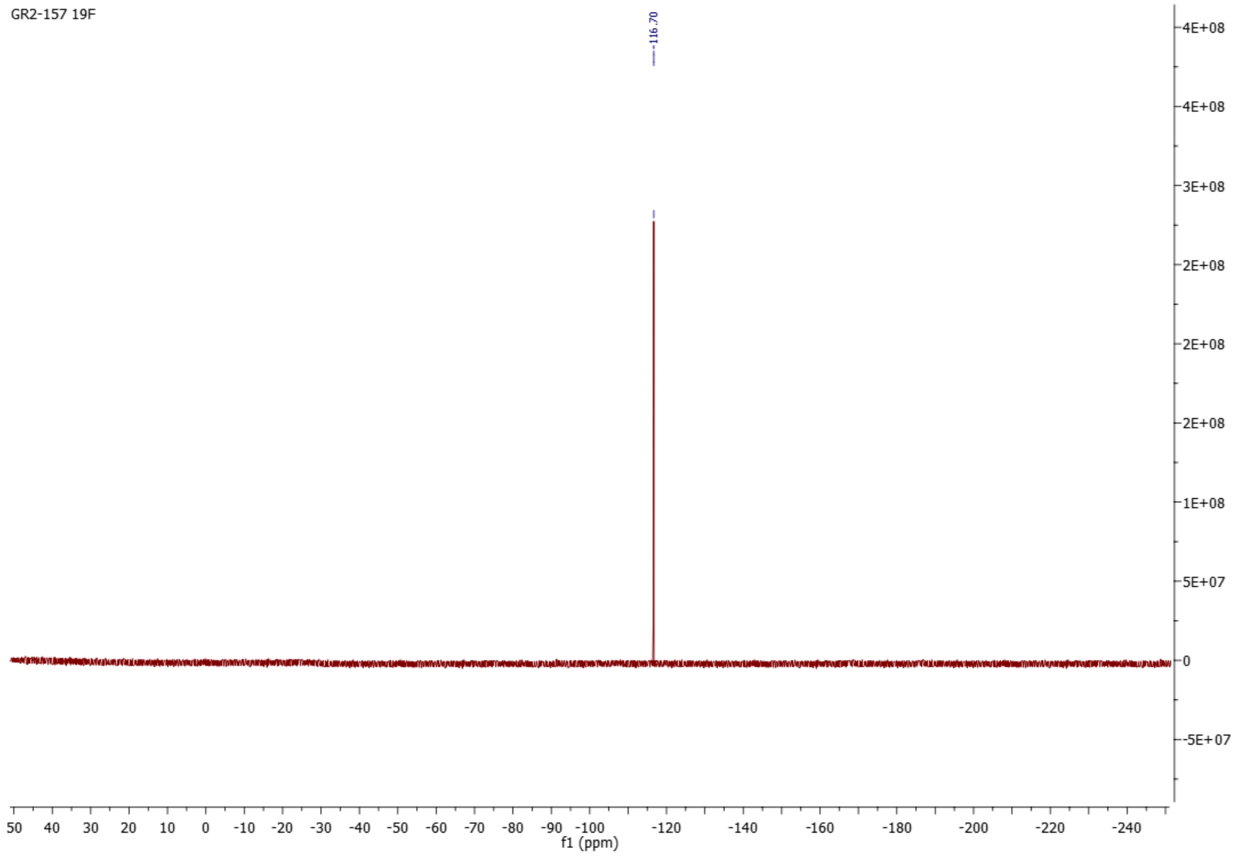

^{19}F NMR spectrum of compound **14b** (376 MHz, DMSO-d_6)

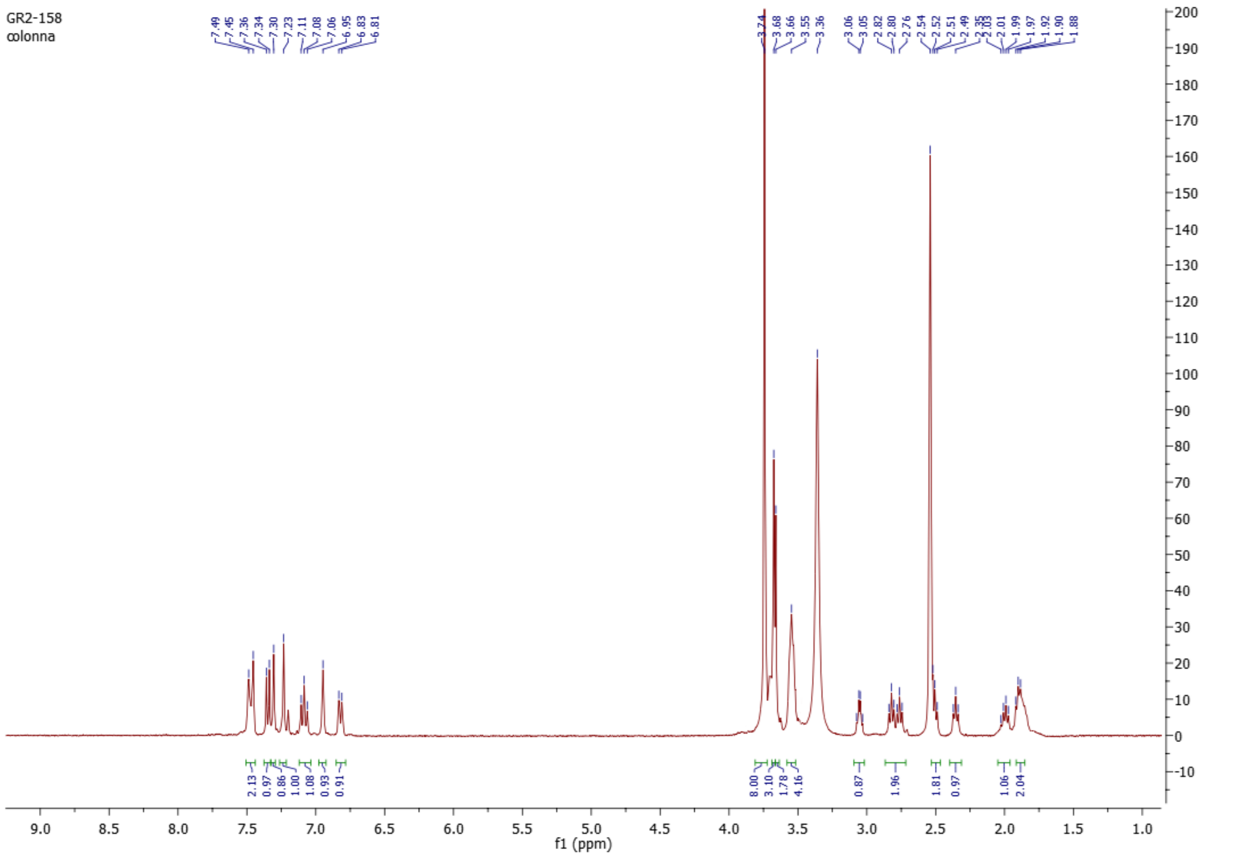

^{1}H NMR spectrum of compound **14c** (400 MHz, DMSO-d_6)

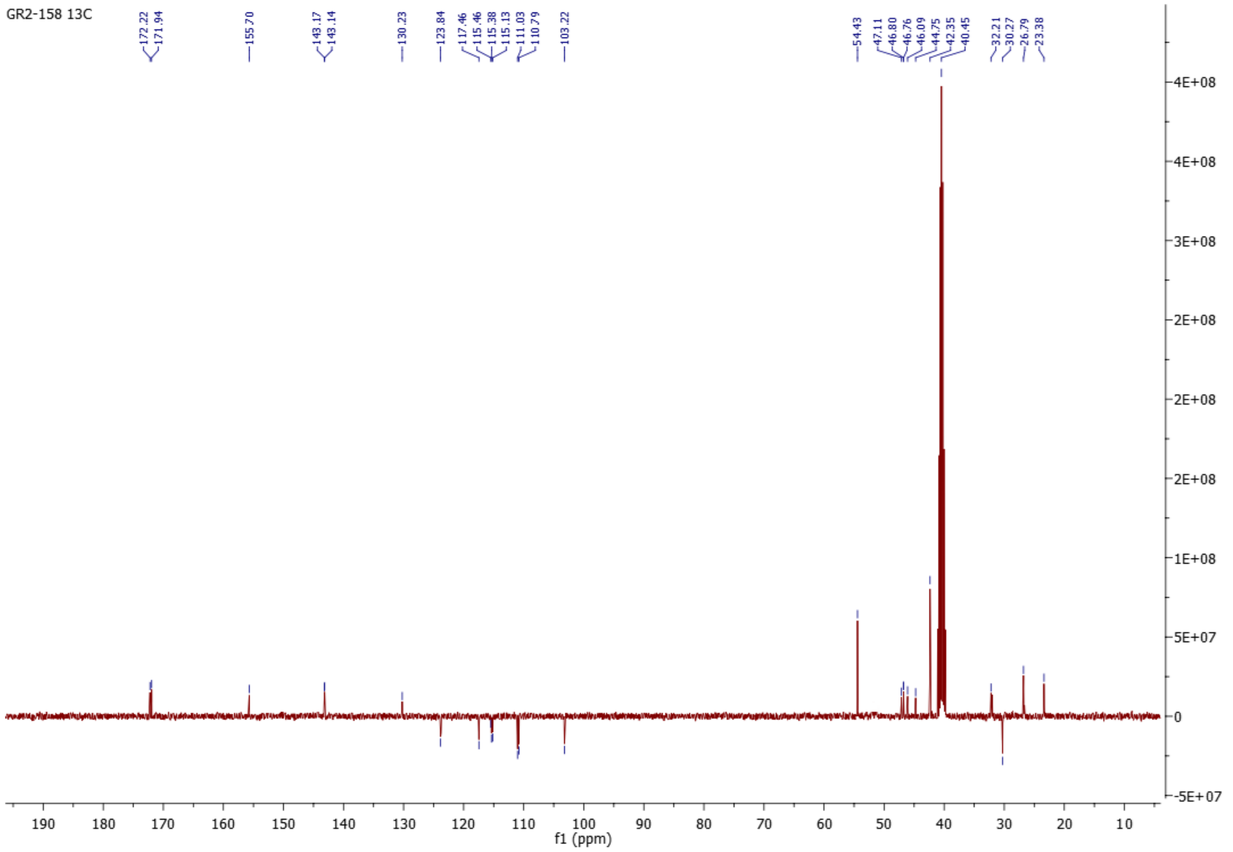

^{13}C NMR spectrum of compound **14c** (100 MHz, DMSO-d_6)

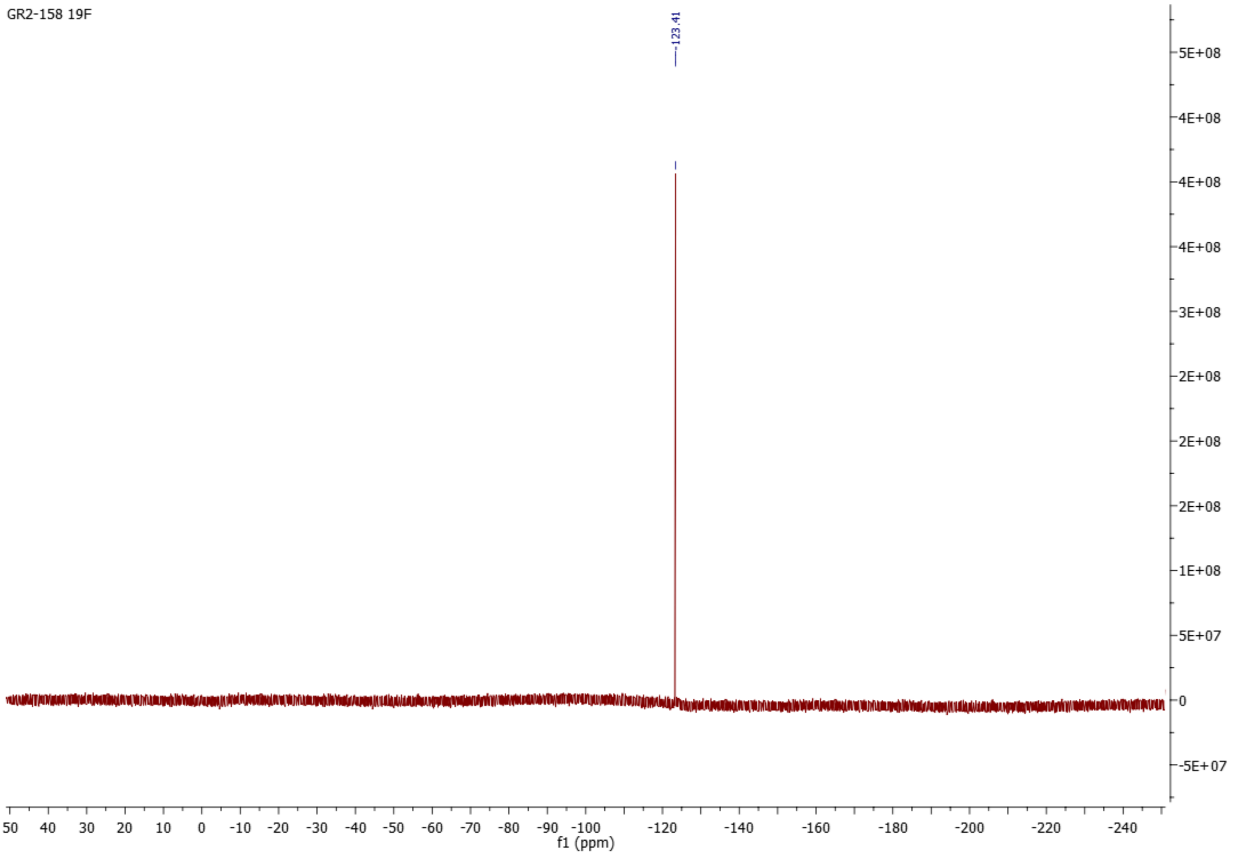

^{19}F NMR spectrum of compound **14c** (376 MHz, DMSO-d_6)

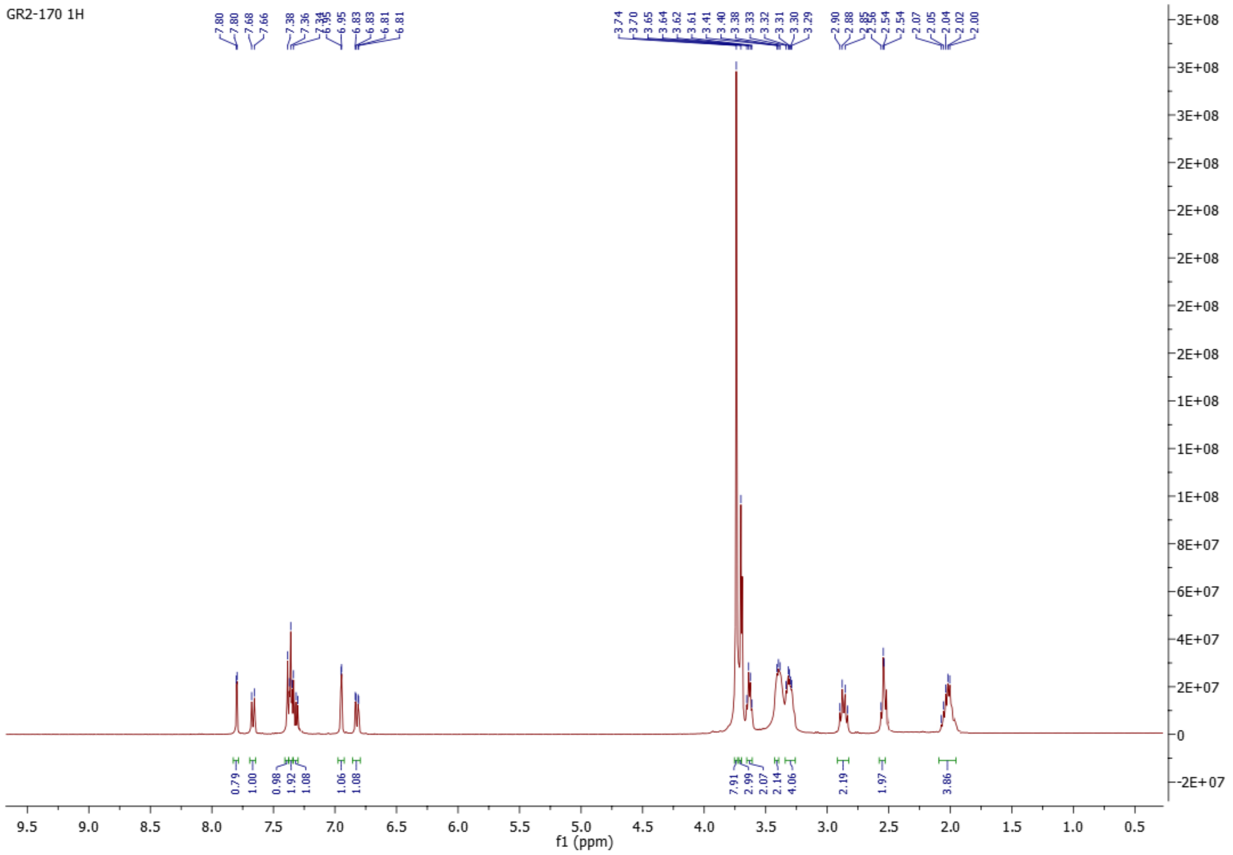

^{1}H NMR spectrum of compound **14d** (400 MHz, DMSO-d_6)

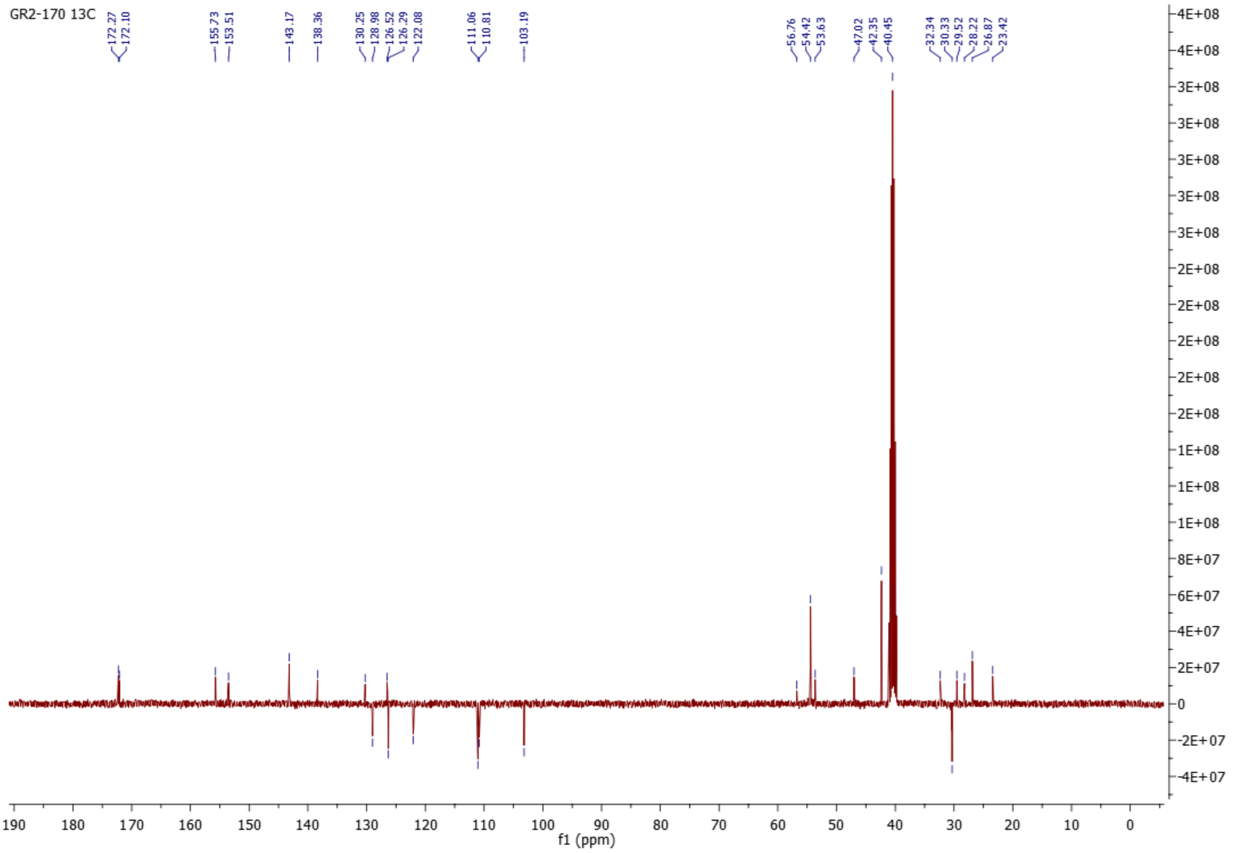

^{13}C NMR spectrum of compound **14d** (100 MHz, DMSO-d_6)

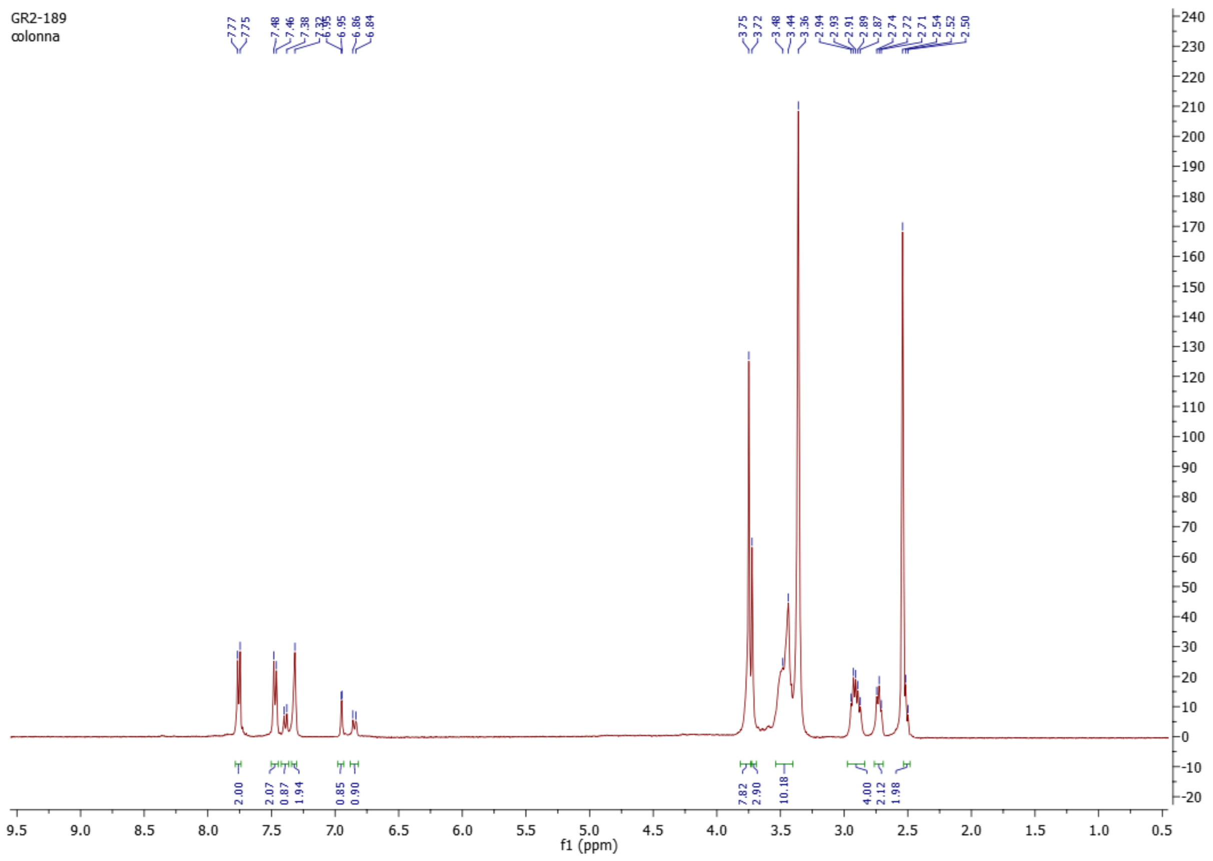

^{1}H NMR spectrum of compound **15** (400 MHz, DMSO-d_6)

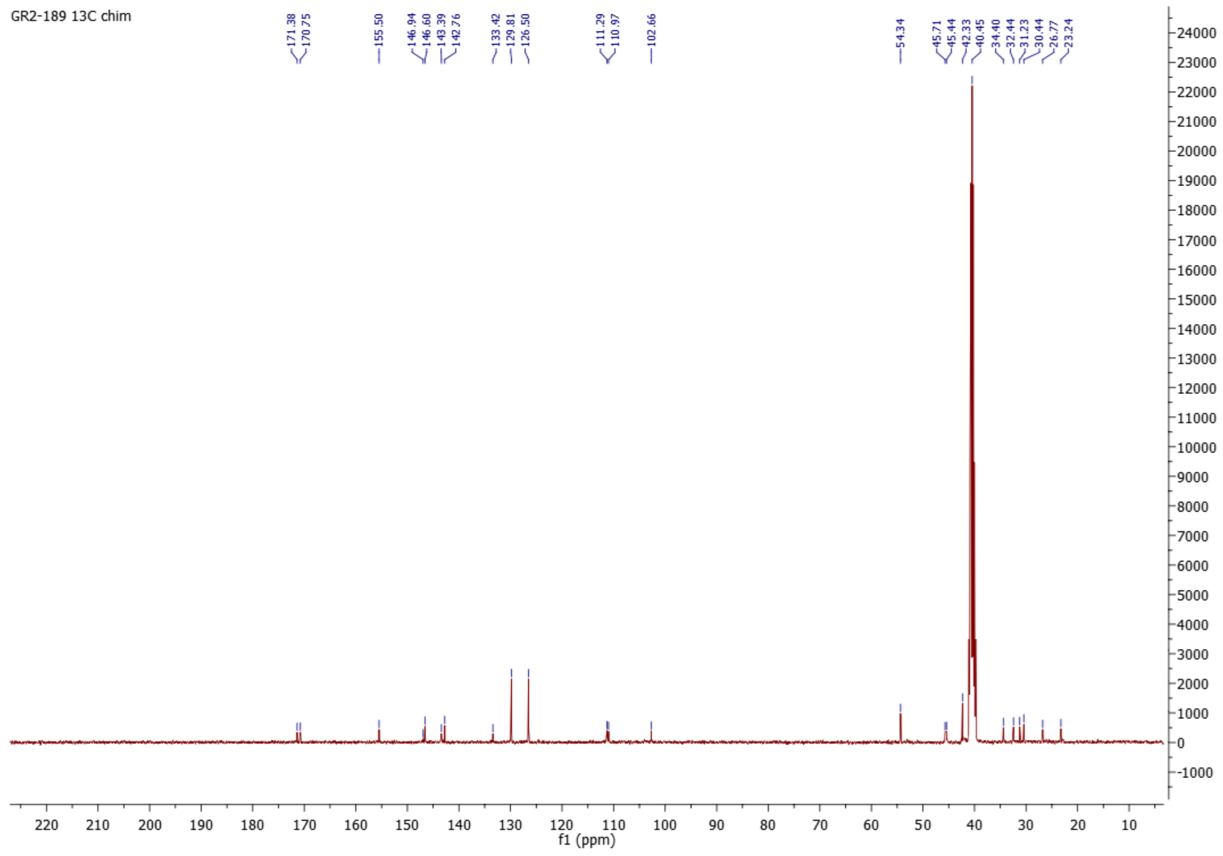

^{13}C NMR spectrum of compound **15** (100 MHz, DMSO-d_6)

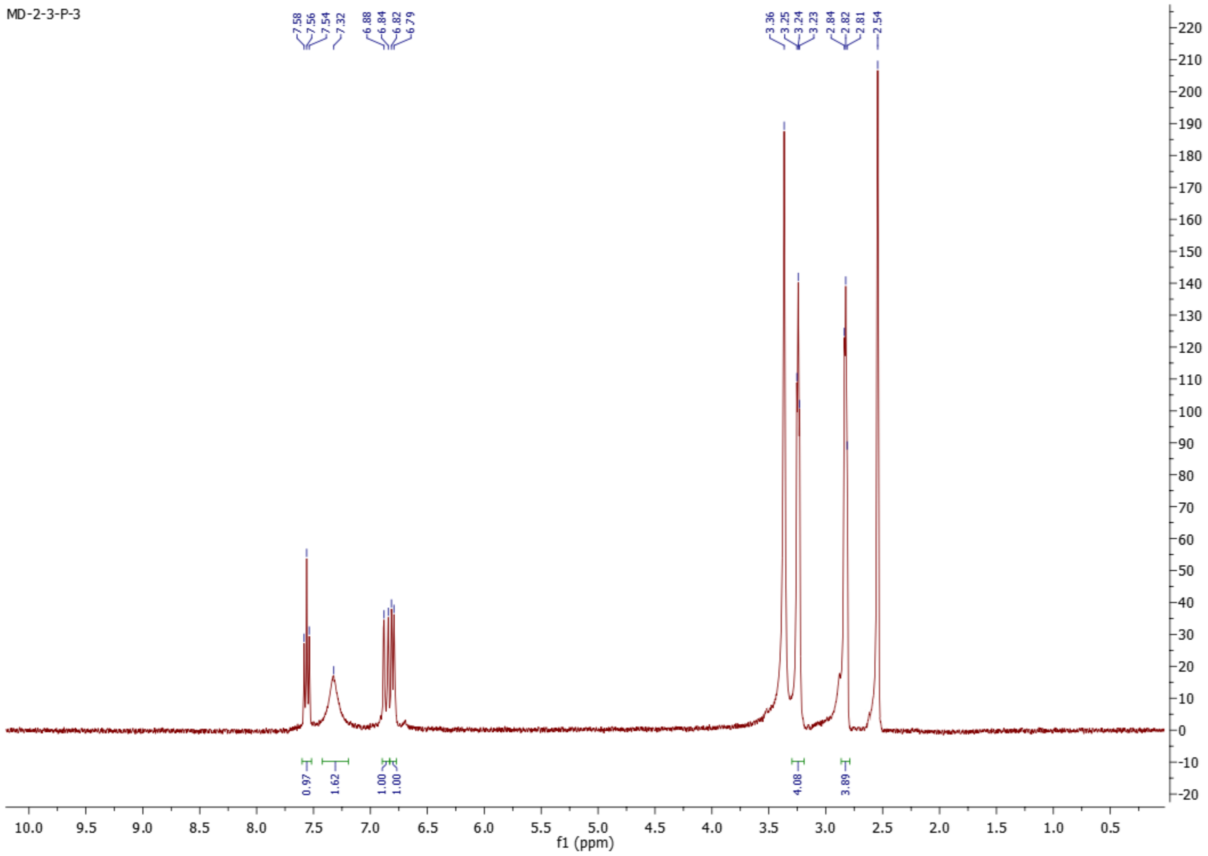

^{1}H NMR spectrum of compound **10f** (400 MHz, DMSO-d_6)

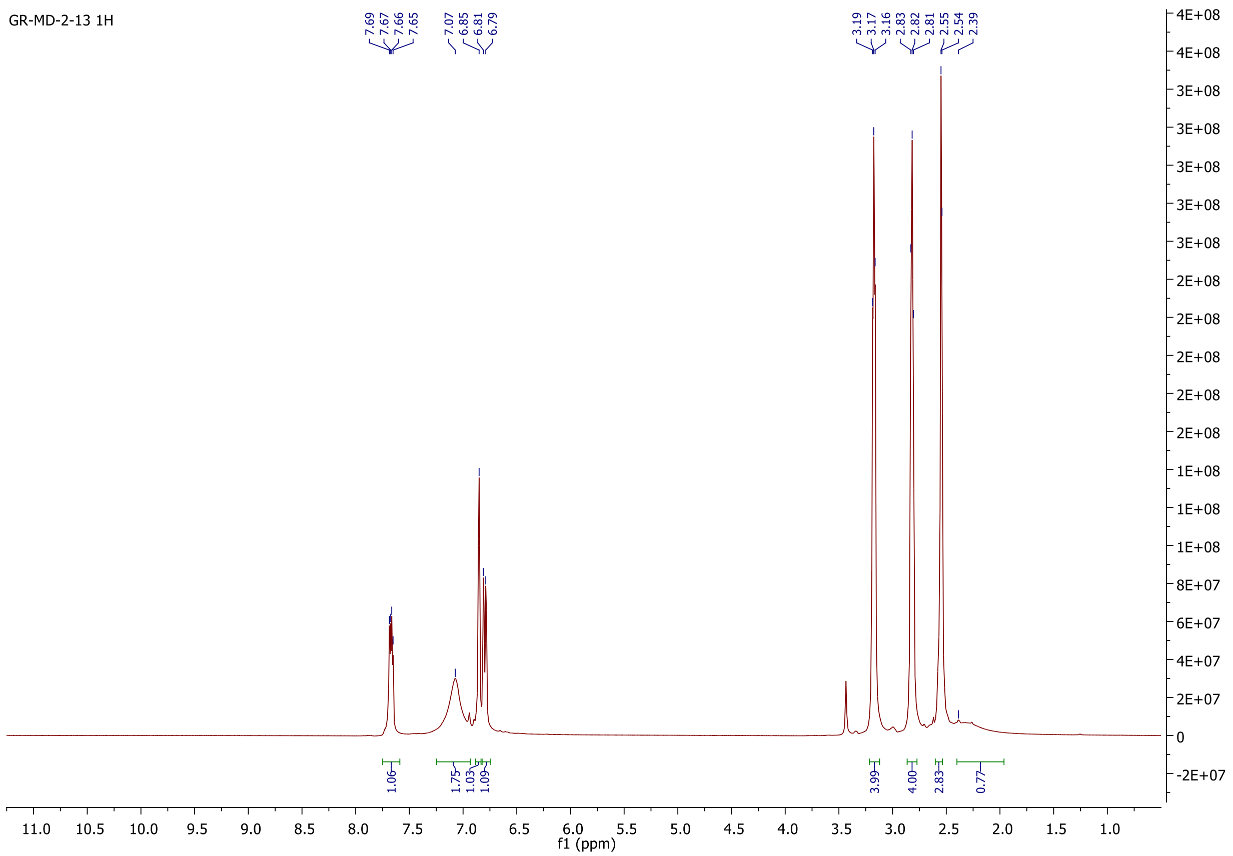

^{1}H NMR spectrum of compound **10g** (400 MHz, DMSO-d_6)

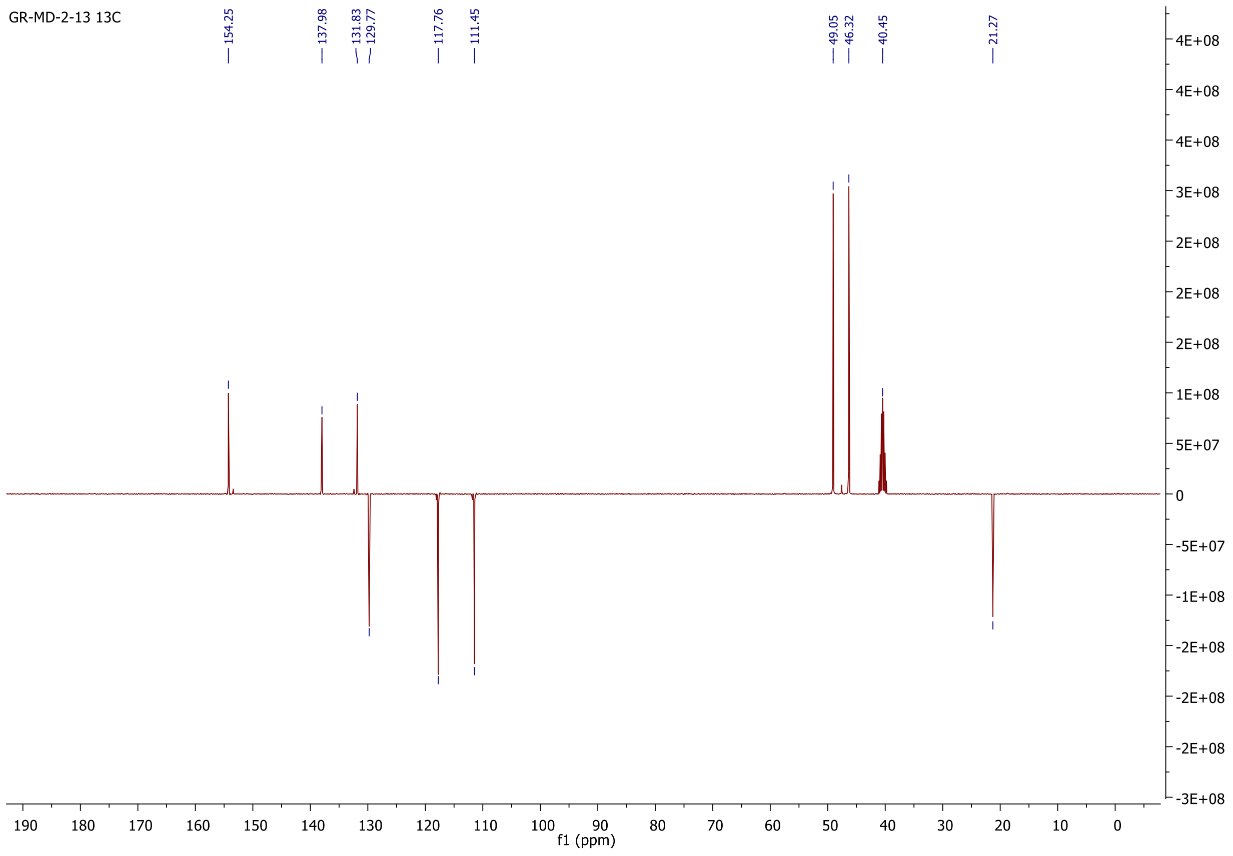

^{13}C NMR spectrum of compound **10g** (100 MHz, DMSO-d_6)

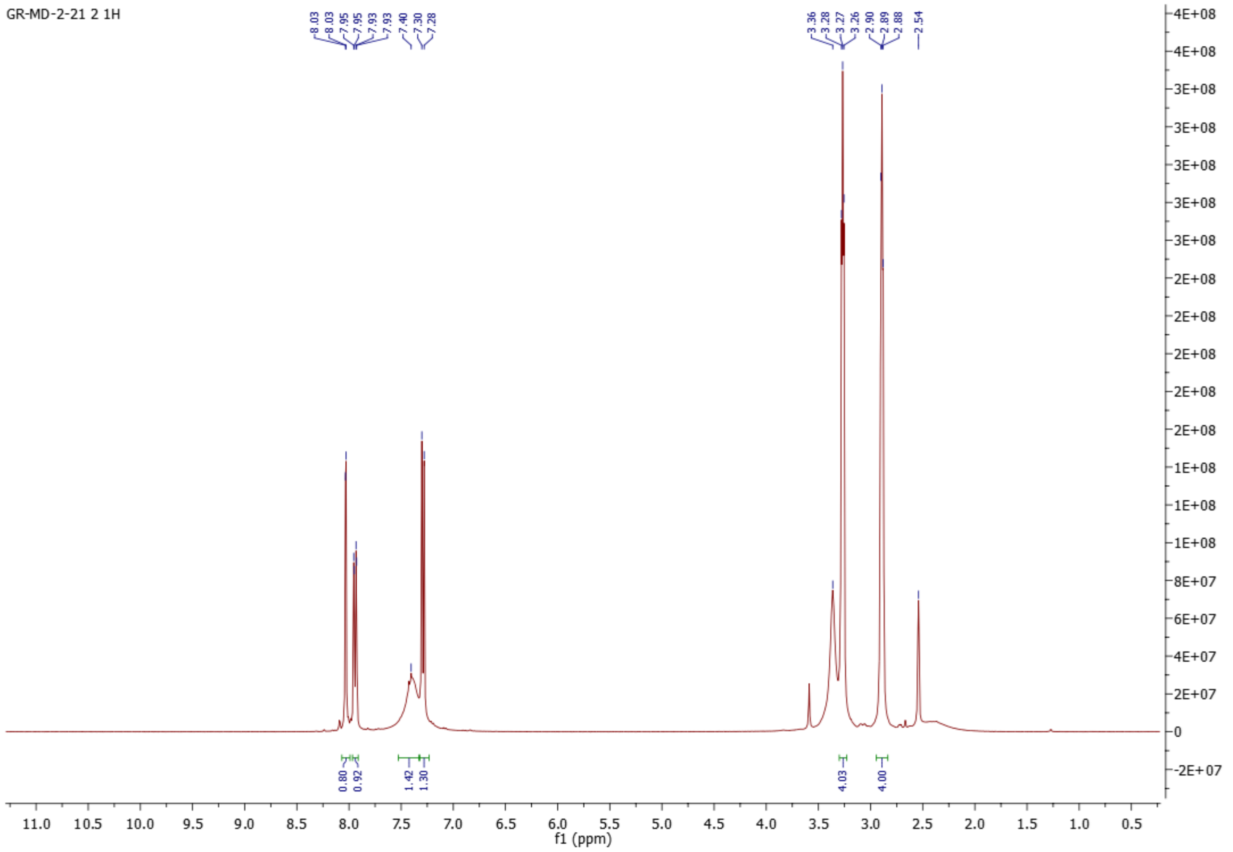

^{1}H NMR spectrum of compound **10h** (400 MHz, DMSO-d_6)

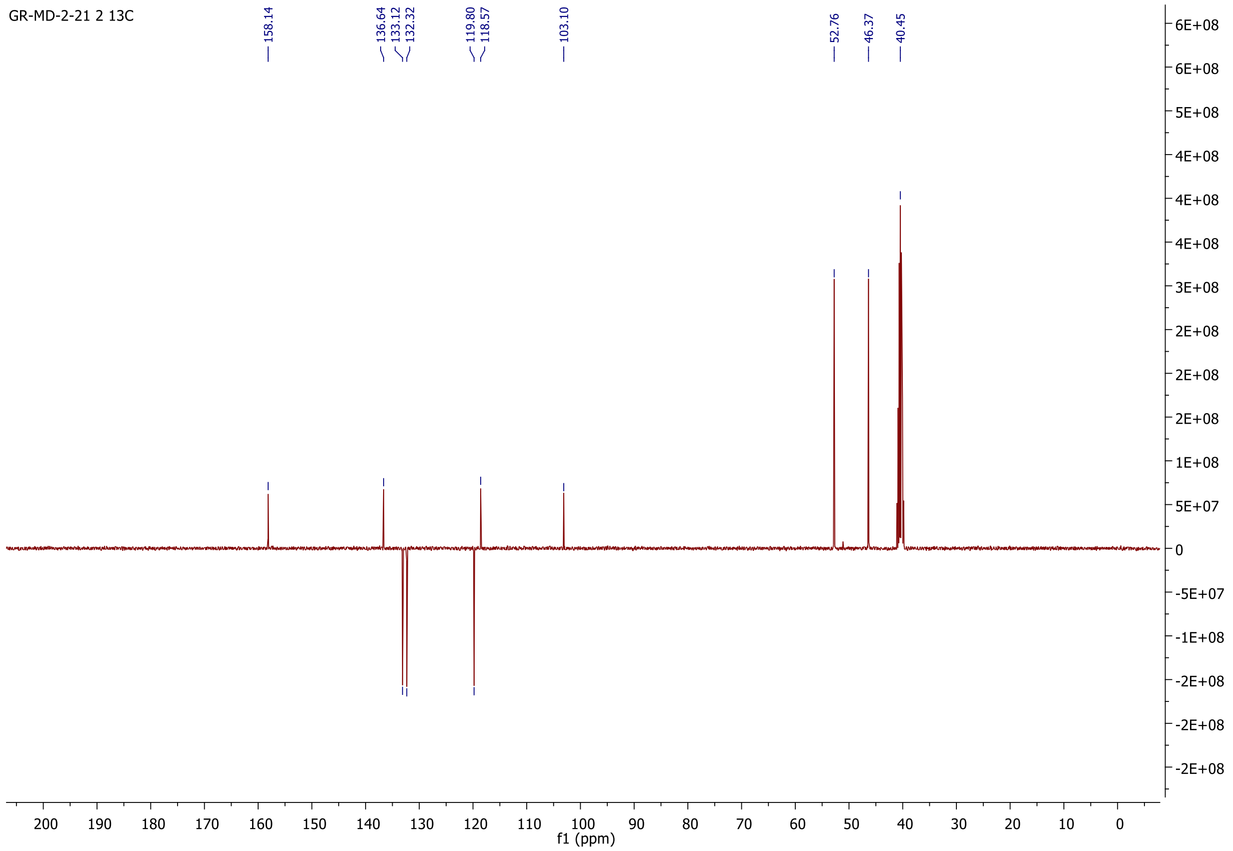

^{13}C NMR spectrum of compound **10h** (100 MHz, DMSO-d_6)

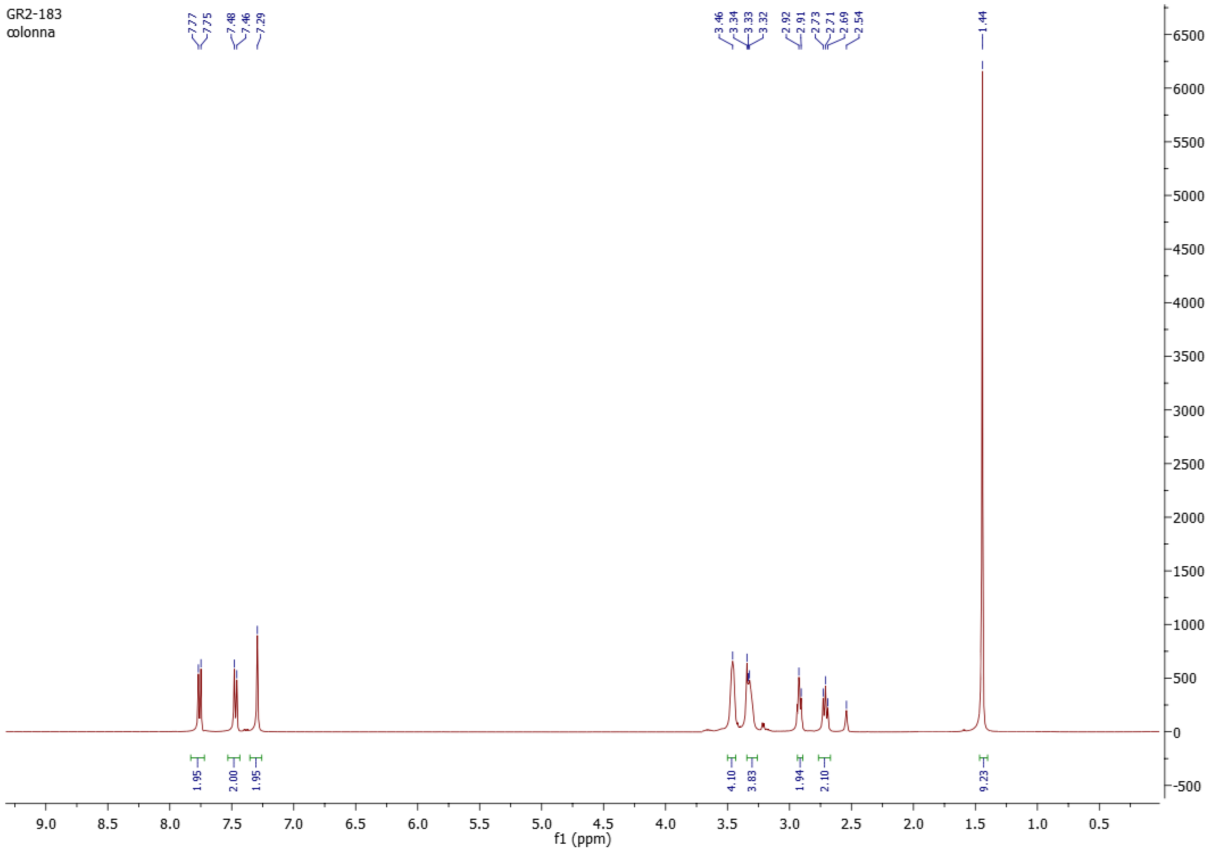

^{1}H NMR spectrum of compound **21** (400 MHz, DMSO-d_6)

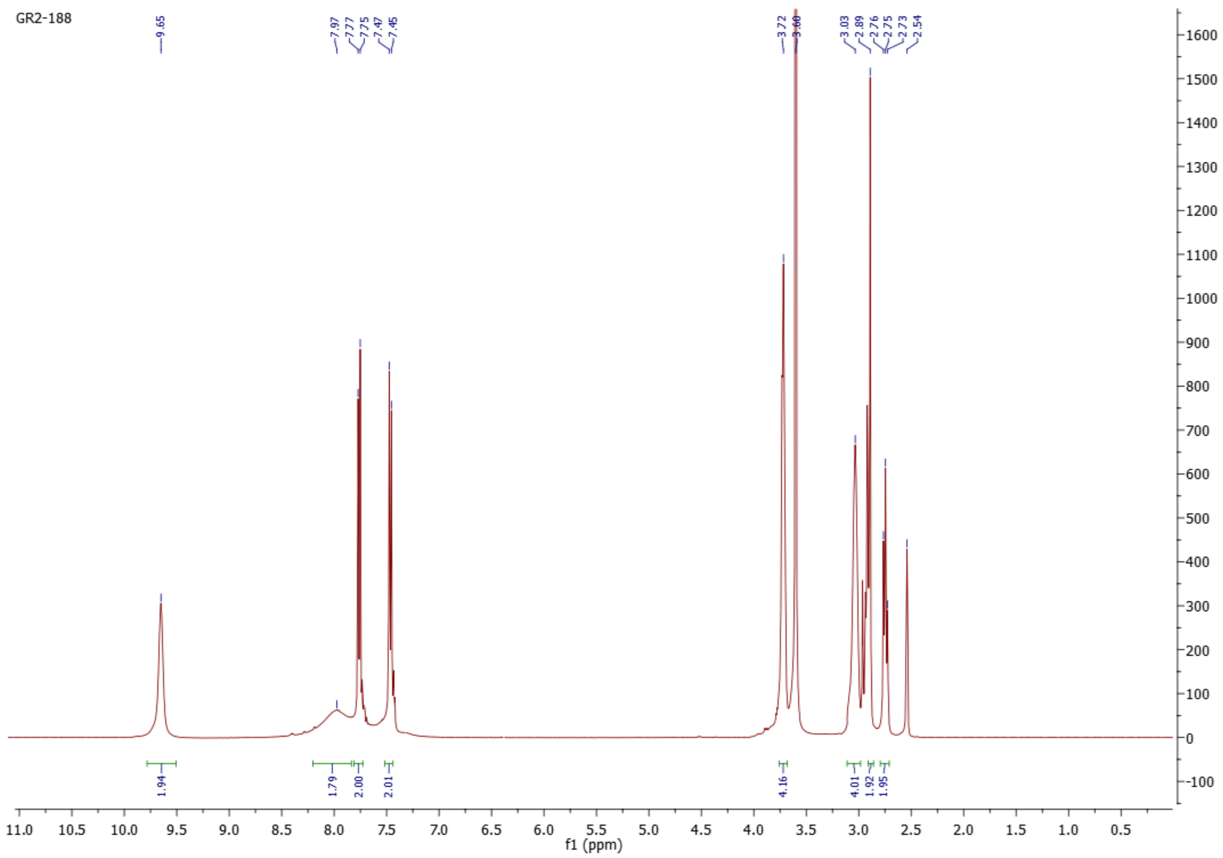

^{1}H NMR spectrum of compound **12** (400 MHz, DMSO-d_6)

Figure S1:

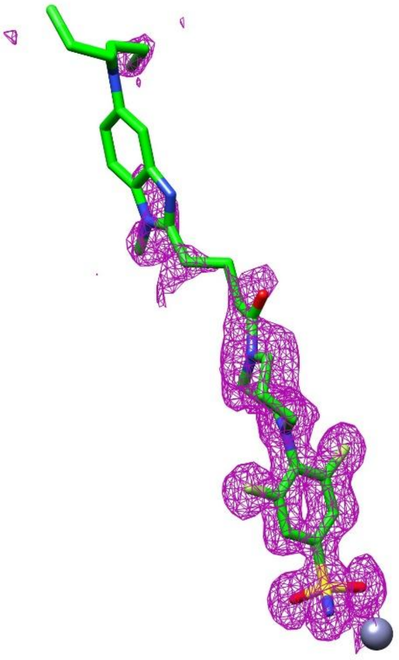

Electron density 2Fo-Fc map of **14b** bound to zinc in hCA II active site; contoured at the 1.0 σ level.

Figure S2:

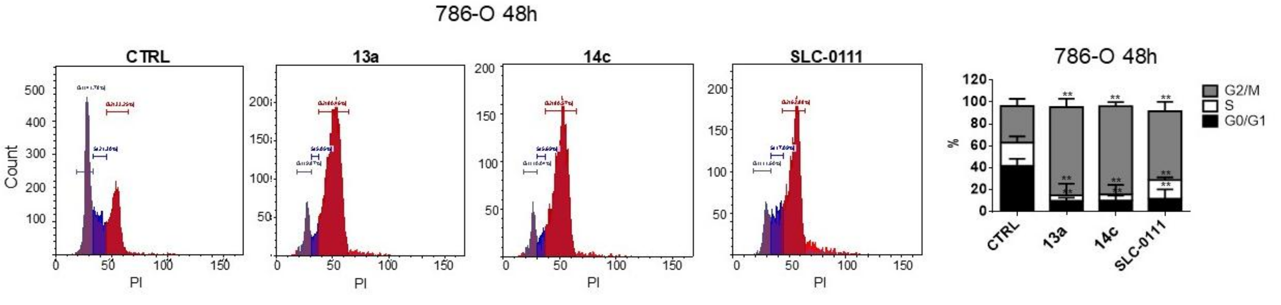

Cell cycle distribution in 786-O cells at 48h after treatment with **13a**, **14c** and **SLC-0111**.

Figure S3:

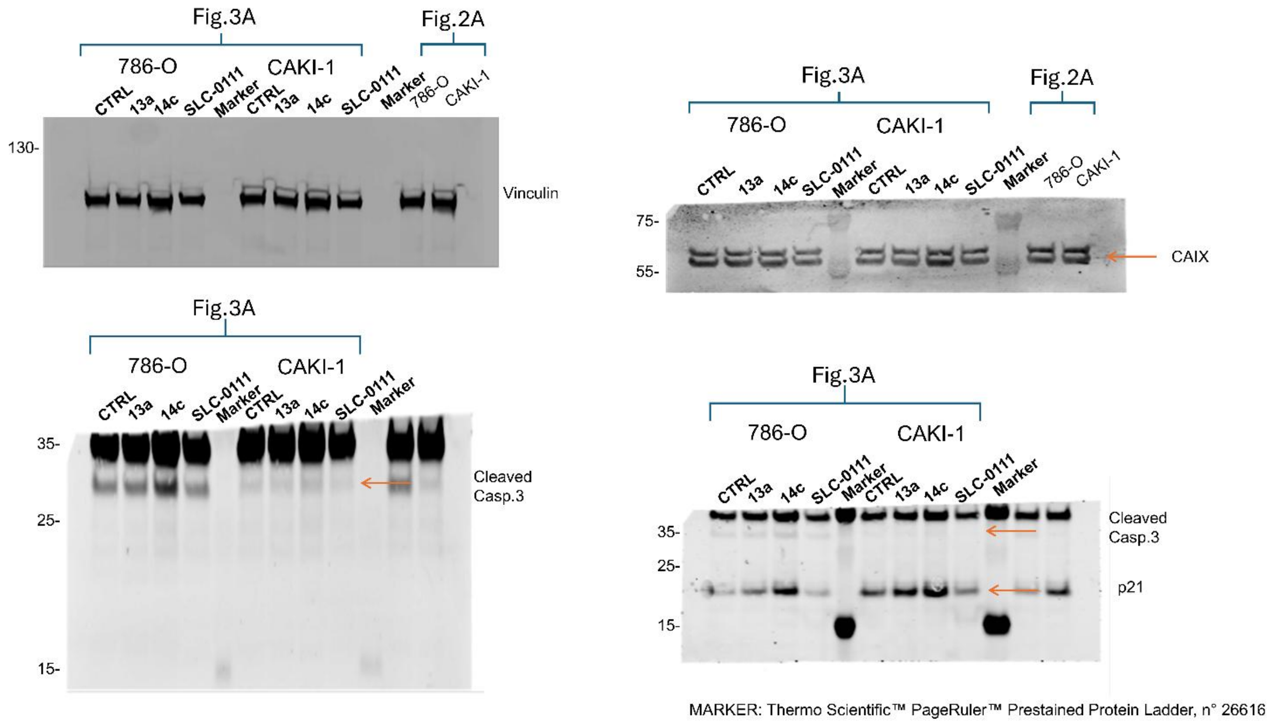

uncropped Western blots (WB) of Figure 2A and 3A

Table S1. Summary of Data Collection and Atomic Model Refinement Statistics for hCAII

	**hCAII + 14b**
PDB ID	30NN
Wavelength (Å)	1.000
Space Group	P21
Unit cell (a, b, c, α, β, γ)(Å,°)	42.39, 41.73, 72.03, 90.00, 104.47, 90.00
Limiting resolution (Å)	1.23-41.08 (1.23-1.26)
Unique reflections	65192 (4651)
Rmerge (%)	6.9 (132.0)
Rmeas (%)	7.5 (150.6)
Redundancy	5.9 (4.2)
Completeness overall(%)	91.0 (88.5)
<I/σ(I)>	12.57 (1.01)
CC (1/2)	0.999 (0.416)
**Refinement statistics**	
Resolution range(Å)	1.23-41.08
Rfactor (%)	13.60
Rfree(%)	16.93
r.m.s.d. bonds(Å)	0.0116
r.m.s.d. angles (°)	1.9233
**Ramachandran statistics** (%)	
Most favored	96.9
additionally allowed	3.1
outlier regions	0.0
**Average B factor (Å^2)**	
All atoms	17.921
Inhibitors	42.020
Solvent	29.628
